# An intertwined triple-bottom-line rating system for highway sustainability in developing countries

**DOI:** 10.1038/s41598-026-35183-4

**Published:** 2026-02-08

**Authors:** Mohamed O. Rageh, Emad E. Elbeltagi, Alaa R. Gabr, Sherif M. El-Badawy, Ibrahim A. Motawa

**Affiliations:** 1 Structural Engineering Department , Mansoura University , Mansoura, Egypt; 2 Department of Civil Engineering College of Engineering , Qassim University , Qassim, Saudi Arabia; 3 Public Works Engineering Department , Mansoura University , Mansoura, Egypt

**Keywords:** Pavement, Rating systems, SDGs, Sustainability, Sustainable highways, surveys, Engineering, Environmental sciences, Environmental social sciences, Environmental studies, Geography, Geography

## Abstract

Highway sustainability evaluation is a crucial step towards ensuring that transportation infrastructure meets the environmental, social, and economic needs of societies. While previous studies have developed frameworks for the sustainability performance of highway projects, they did not comprehensively address all sustainability aspects, nor stages of these projects. Furthermore, the Sustainable Development Goals (SDGs) related to highways were almost overlooked in most previous studies and infrastructure rating systems. Thus, this study introduces a rating system (RS) that encompasses the most significant factors influencing highway sustainability in Egypt across the three sustainability dimensions. These factors were identified through a comprehensive analysis of previous studies, SDGS, and existing infrastructure rating systems. A questionnaire survey was designed to assess the impact of various factors on highway sustainability. Statistical analyses were carried out to summarize data into a smaller set of factors, identifying 18 factors that were weighted using the analytic hierarchy process method. This model was then applied to real-life case studies in Egypt, and the results were compared with the Envision RS. The results’ convergence confirmed the validity of the developed RS, which features fewer factors and a more user-friendly interface. Finally, the developed RS was reviewed by highway experts and environmental consultants to assess its performance and gather their recommendations. The developed RS presents a summary report that identifies shortcomings in achieving the target score and offers guidelines to enhance the sustainability score. Also, it addresses the shortcomings found in other RSs, providing a more balanced approach across the investigated sustainability dimensions.

**Supplementary Information:**

The online version contains supplementary material available at 10.1038/s41598-026-35183-4.

## Introduction

Highway networks play a crucial role for communities, facilitating the movement of people and goods. Investing in infrastructure projects, such as highways, bridges, tunnels, and airports, is essential for economic growth. However, these projects may have negative impacts, potentially conflicting with sustainability goals, as transportation activities contribute significantly to Greenhouse Gas emissions (GHGs). Hence, highway projects may harm the surrounding environment causing nuisance, disrupting human life and ecosystems, polluting soil and water, and destroying biodiversity during their construction. Furthermore, the materials used for pavement construction emit GHGs during their extraction, processing, transportation, construction, and disposal at the end of their service life. To our knowledge, the highway industry is a major consumer of materials, requiring large quantities of raw and manufactured resources for both construction and maintenance. Carbon dioxide (CO_2_) emissions from the construction and maintenance of highway projects are estimated to be 10% higher than those of vehicles using the highways during the operation stage^1^. In addition, the fast economic development of China, Brazil, India, Mexico, Indonesia, and other overcrowded countries increases the need for highway transportation, which increases the consumption of natural resources and GHG emissions^2,3^. Conversely, significant economic losses occur due to the ongoing need for substantial capital and long-term investments required for highway construction projects.

Studies mentioned a high probability of injuries and fatalities on-site, especially in developing countries^4^, as construction sites frequently overlook the safety and health of workers. In 2016, Enshassi et al. illustrated that the outcomes of the accidents in the construction sites not only stop at causing pain and suffering, but also cause negative effects on the project aims in terms of productivity, quality, time, and cost^5^. Also, the constructed highway safety targets to increase mobility while maintaining an acceptable degree of safety, even though these projects are infamous for traffic accidents^6^. Traffic accidents are a dreadful and costly issue for both the social and economic sustainability aspects. Socially, more than 1.19 million people pass away every year in highway accidents, in addition to injuries for about 20 to 50 million people. Economically, the cost of traffic accidents accounts for almost 3% of countries’ national income^7^. On the other hand, enhancing the level of highway services and reducing travel time have become urgent needs that can be achieved by increasing the capacity of highways, improving the project employees’ skills by providing training programs, and solving the unemployment issue by presenting decent job opportunities from the project’ development.

Hence, there is an interrelationship between promoting sustainability in one dimension and its connection to other dimensions to conduct a highway project, considering a balance among the sustainability aspects. Therefore, the socio-economic and environmental aspects of highway construction and maintenance projects should be evaluated^8^.

Despite the support from the UN for SDGs and related factors such as water, energy, climate, transport, urbanization, and technology, these factors have not been clearly addressed in previous studies or infrastructure RSs. Moreover, international infrastructure RSs and previous studies primarily focus on environmental aspects, materials, and energy systems, leaving out other aspects and the comprehensive consideration along the project life cycle, including construction, maintenance, and operations stages^9^. These RSs were developed in the USA, Canada, the UK, and Australia, highlighting their limitations in addressing the specific needs and socio-economic conditions of developing countries similar to Egypt. These conditions are related to the high population growth rate, the high unemployment rate, debts, inflation, and lack of cash reserves. Therefore, this study aims to develop a comprehensive RS for evaluating HWS in Egypt, addressing the triple-bottom line of sustainability through the highway life cycle, and considering SDGs. In this study, the most significant factors affecting highway sustainability (HWS), social, economic, and environmental, are thoroughly evaluated and summarized to develop a rating system (RS) for HWS assessment. A questionnaire survey was carried out to assess the impact of these factors on HWS. Statistical analyses were applied to summarize the data into a smaller set of highly correlated factors. The Analytic Hierarchy Process (AHP) was then used to assign weights to these factors. The developed RS was applied to real case studies, and the results were compared with another international RS to ensure its validity. As such, this study resulted in developing a rating system for evaluating HWS in Egypt considering SDGs. Moreover, conducting such an RS will help evaluate the sustainability performance of highway projects. Furthermore, highway projects certified by the developed RS prioritize awarding contracts to contractors based on the technical evaluation of their bids.

The remainder of this paper is organized as follows: the third section presents the steps for the data collection and analysis. The fourth section includes the discussion of the HWS assessment model and model automation, in addition to the results of the applied case studies and the model feedback. The fifth section provides the implications of this research. Finally, section six provides the research conclusion and recommendations.

## Literature review

The notion of sustainability emerged during the United Nations (UN) Conference on the Human Environment held in Stockholm in 1972. This concept defines sustainable development as “it is a development that meets the present needs without discording future generations’ capacity to achieve their demands”^10,11^. Infrastructure sustainability has been widely known since the 1990s^12–14^, unlike green RSs for buildings that have been utilized for a long time. Sustainable infrastructure has been taken into consideration due to its environmental, economic, and social impacts, and should be incorporated through the planning, design, construction, operation, maintenance, rehabilitation, and demolition stages^15,16^.

On the other hand, sustainable development consists of a set of environmental, economic, and social issues originated from the UN Environment and Development Conference, as it showed that CO_2_ emissions should be curtailed to realize sustainable development^17^. In 2015, the member states of the UN developed a common plan for peace and growth for people and the planet, which determined seventeen SDGs as an urgent call for action by all developed and developing countries^18^. Each goal consists of a set of targets with pre-defined indicators (i.e., measures) that are related to all aspects of life.

In this context, many countries have started considering the principles of sustainability. For instance, Colombia has reported on its investments in fortifying infrastructure to support economic restructuring and economic growth to achieve greater prosperity^19^. Furthermore, the Russian Federation has provided investments of more than 225 million dollars to upgrade its national railways and highways. Ethiopia and Kuwait also have increased public transport sharing in urban areas to lower Co_2_ emissions^20^. For Tajikistan, it has invested 2.5 billion dollars in 59 transportation projects for more than 1,500 km of highways, aiming for more internal and external mobility. Moreover, Egypt has begun to construct highways and integrated infrastructure that connect remote areas, which will support the economy and achieve Vision 2030, although facing a lot of challenges, including population growth.

**Fig. 1:**
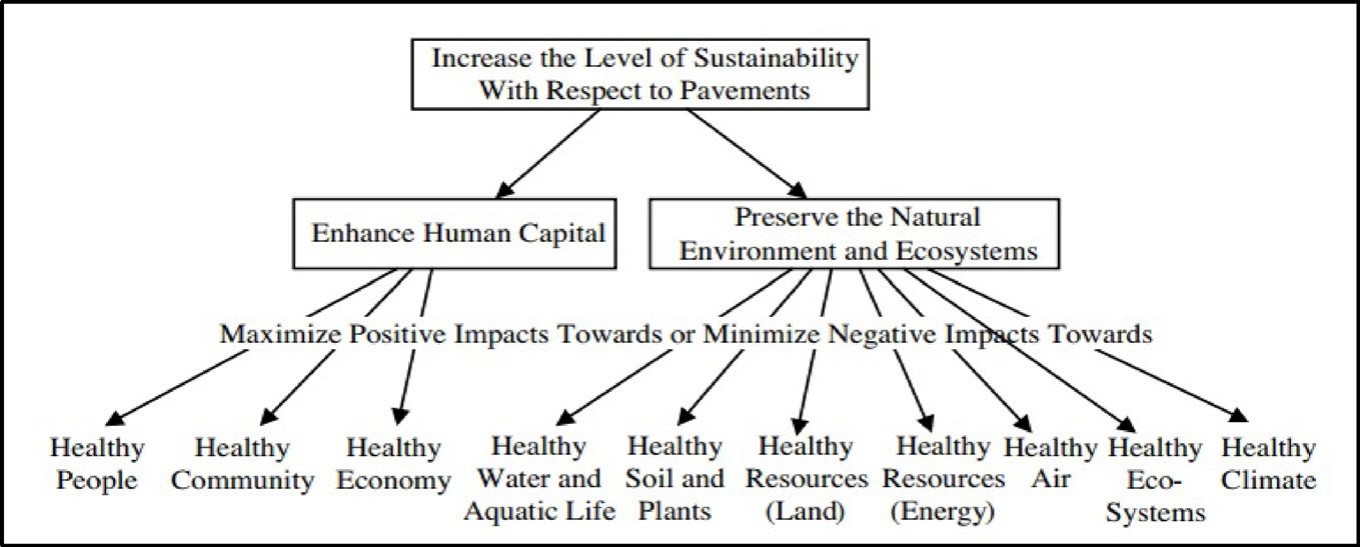
Indicators of sustainable enhancement in pavements **(Bryce et al., 2017).**

Nevertheless, various research initiatives have aimed to develop tools for assessing HWS. For example, a study was carried out in 2012 to develop a framework that contains 60 sustainable practices for highway design through reviewing sustainable requirements of the LEED, Global Reporting Initiative, relevant studies, and practitioner interviews, which was created and tested on four real-life projects to validate their applicability on highway designs^21^. The proposed framework is intended to narrow the gap between theoretical requirements and practical aspects to direct the construction projects toward sustainability. Moreover, another guidance tool has been developed to help decision-makers and technical specialists in the highways sector achieve more sustainable highway projects along their life cycle in developing countries^22^. In 2017, a model was presented for evaluating highway sustainability, which developed a hierarchy structure to link sustainability objectives with defined indicators^23^. As illustrated in Fig. 1, the base-level objectives of the structure were extracted from sustainability assessment tools, including BE^2^ST-in-Highways, Greenpave, Greenroads, Invest, Envision, and GreenLITES.

In another study, an index was created to assess the sustainability performance of highway projects named the Composite Highway Sustainability Index (CHSI). This study considered the environmental and economic aspects that only existed in previous studies, ignoring the comparison with other RSs, and considering SDGs^24^.

Furthermore, a study was carried out to evaluate transportation sustainability in the UAE named the Green Pavement Rating System (GPRS-UAE), which evaluates the structural design, asphalt pavement and materials, construction stage, rehabilitation, and management processes. While the developed RS is considered one of the first efforts in the UAE aiming to produce better sustainable asphalt pavements, this work did not include a case study to validate the developed RS^25^.

Another study suggested a rating system for evaluating HWS in Nigeria. The research identified 36 specific sustainability assessment indicators to support the implementation of highway design, as each indicator assigned a credit point. Additionally, it proposed a Smart Green Certification level to strengthen the systematic validation of developed guidelines for highway design. However, the study subserved short-term development schemes that create a gap between theory and practice in achieving highway sustainability. Moreover, the design stage missed incorporating quantified sustainability concepts^26^.

Moreover, an initiative was carried out to construct a rating system for developing countries and the considered indicators were derived from Agenda 21 and the SDGs. Information collected from UN agencies, public development institutions, and development banks. Although the RS primarily focused on health and education, it has only a minority of indicators that address other sustainability aspects. This study relied on the judgments of international experts to determine the indicators’ weights through an AHP survey and emphasized the high importance of considering SDGs through the HWS evaluation^9,27^.

In another approach, a framework was developed for selecting the most sustainable road design, considering the life-cycle cost analysis and social life-cycle assessment to assess each design systematically and comprehensively^28^. Twelve measurable indicators were determined and weighted using AHP analysis, which evaluates the impacts of a certain highway design during its service life. Furthermore, another integrated approach for sustainability assessment and reporting for civil infrastructure projects has been carried out, considering the UN-SDGs through the triple sustainability dimensions^29^. The study resulted in determining 17 factors affecting the sustainability performance of infrastructure projects (i.e., 9 social, 6 economic, and 7 environmental factors), in addition to applying the proposed framework on civil infrastructure projects from Europe and Asia.

Although many efforts and initiatives have been carried out to develop sustainability evaluation RSs that promote the design and management of sustainable infrastructure projects, they have several shortcomings^23^. One of these shortcomings is that many infrastructure RSs do not focus on pavement materials or paving activities, which should be studied to quantify their impact on sustainability objectives. Furthermore, some infrastructure RSs do not consider all sustainability dimensions. For instance, although the Civil Engineering Environmental Quality Assessment and Award Scheme (CEEQUAL) supports clients, designers, and contractors in dealing positively with environmental quality issues relevant to the project, it greatly ignores the economic aspect, unlike other infrastructure RSs. On the other hand, most of the previous studies and infrastructure RSs have not assessed HWS in developing countries, such as Egypt. In addition, they have overlooked various stages of highway projects and the social sustainability aspect, focusing primarily on the environmental aspect^23,30^. In conformity with the highway industry’s role in supporting SDGs, it is essential to address all relevant factors to the triple-bottom line of sustainability, which significantly impact HWS. To this end, the most significant factors affecting HWS, social, economic, and environmental, are thoroughly summarized and evaluated to develop a comprehensive RS for HWS assessment in Egypt through the project lifecycle.

## Materials and methods

This study aims to develop a comprehensive RS to evaluate HWS in Egypt, considering the three dimensions of sustainability through the project life cycle. Furthermore, the socio-economic aspects and SDGs are considered. Hence, the proposed RS incorporates significant factors from previous studies, the SDGs, and existing infrastructure RSs shown in Table S1 (in the supplementary).

### The research methodology

**Fig. 2:**
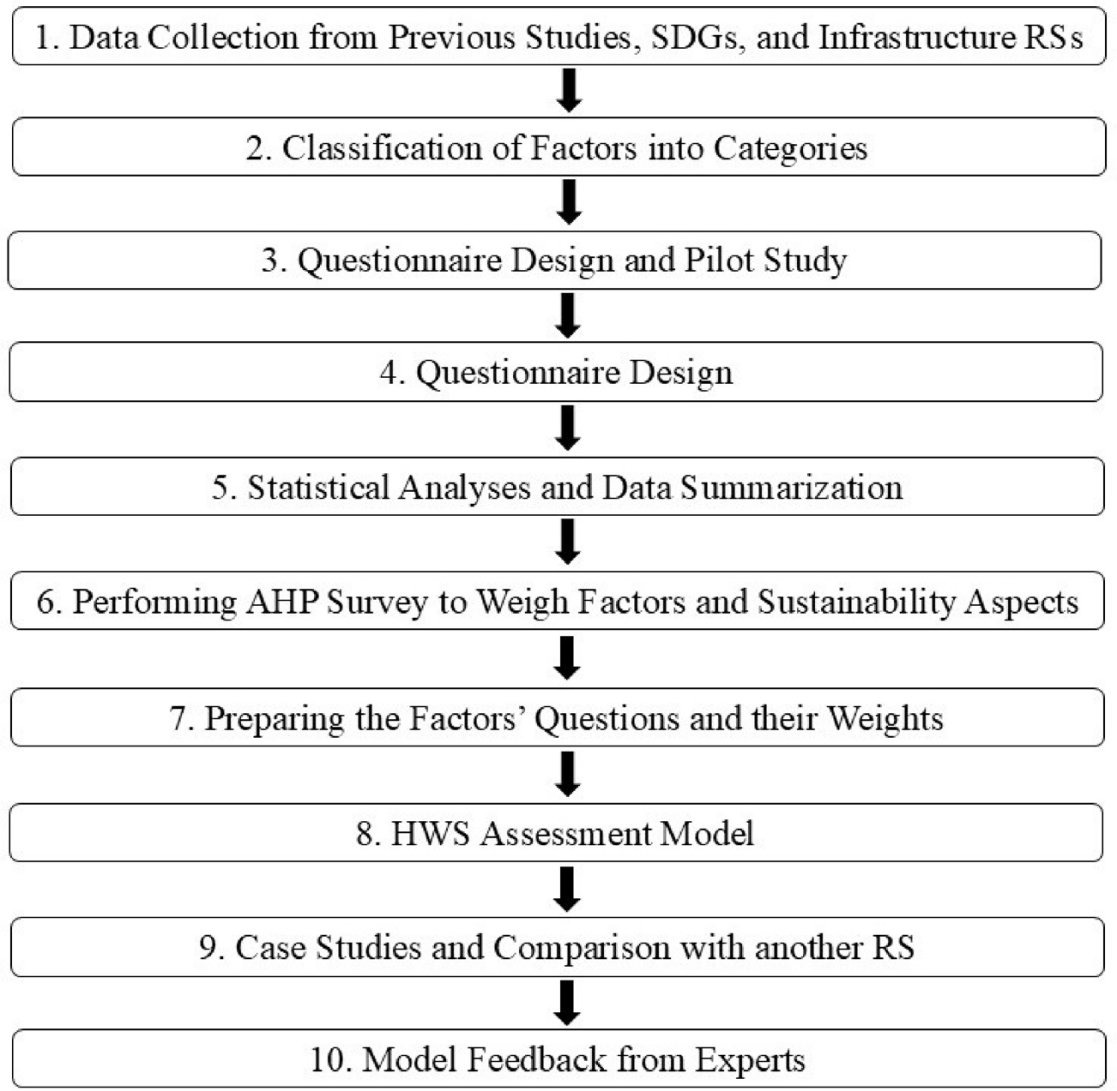
Summary of the research methodology.

As illustrated in Fig. 2, the most significant factors affecting HWS were determined. The factors were classified into six categories. A questionnaire survey was designed and piloted to test the effectiveness and clarity of the selected factors and the questionnaire design. The questionnaire was then finally developed to assess the impact of different factors on HWS using a five-point scale, along with all necessary statistical analyses needed to validate its reliability.

The RII, PCA, and Pareto principle were used successively to condense the factors into a smaller set of highly correlated factors, which were then weighted using the AHP by developing four pair-wise comparison matrices. A scale and a set of questions were suggested and weighted for each factor across the three sustainability pillars by developing a questionnaire survey with experienced highway engineers. Then, this model was applied to real-life case studies, and the results were compared with another infrastructure RS to ensure its validity. Finally, the model was reviewed by highway experts and environmental consultants to assess its overall performance and gather their recommendations for presenting the model in its final enhanced form.

### Data collection

In a previous study, the most significant factors affecting HWS have been identified from a list of factors that were collected from published articles, SDGs, and infrastructure RSs (Text S1, Table S2 in the supplementary)^31^. This comprehensive analysis identified 78 factors that significantly influence HWS (Table S3 in the supplementary).

Infrastructure RSs did not explicitly consider the announced 17 SDGs, especially those that were developed prior to the adoption of the SDGs in 2015. Though, infrastructure RSs were designed to promote the concepts of sustainability, resilience, and efficiency in infrastructure projects, as many of their criteria align with the objectives of the SDGs and support similar principles. For instance, Greenpave RS focuses on sustainable pavement practices and incorporates principles that support SDGs related to sustainable cities and communities (SDG 11) and climate action (SDG 13), emphasizing the importance of environmentally friendly materials and practices. Furthermore, Greenroads RS assesses roadway projects on various sustainability criteria, encouraging practices that contribute to environmental sustainability and community well-being, which supports multiple SDGs.

**Fig. 3:**
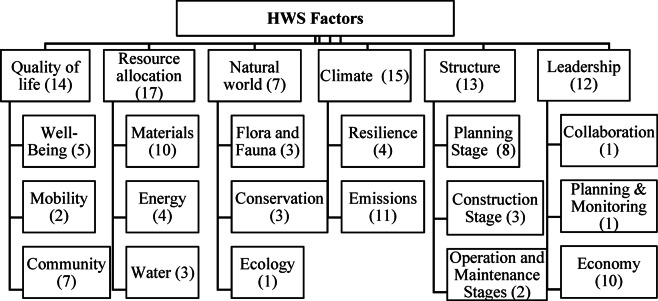
Factor breakdown structure.

As “Envision” is one of the most shared RS with 67 factors out of the total 224, listed in Table S2 (i.e., 21 out of the most significant 78 selected factors that affect the HWS, listed in Table S3)^31^, it was selected as a guide for the factors’ categorization. Envision RS evaluates sustainable infrastructure across various categories, including quality of life, leadership, and resource allocation. Its categorization aligns with the objectives of the SDGs by promoting responsible use of resources, sustainable community development, and climate resilience, focusing on the social and environmental aspects (Table 1). Hence, all categories and sub-categories of “Envision” were analyzed to assign factors to the proper categories. A sixth category named “Structure” was added to include all factors related to the highway life cycle stages. The factors were classified to determine the assigned relative weights for each category as a result of the experts’ opinion. Categories and sub-categories with related factors are shown in Fig. 3. Table S4 (in the supplementary) presents the distribution of the selected factors in each sub-category.

**Table 1:** Mapping of analyzed social and environmental factors existed in the SDGs and envision RS.

Factor	SDGs	Envision	Factor	SDGs	Envision
FE1			FE53	√	
FE2	√	√	FE56		√
FE5	√	√	FE57		
FE7			FE60		√
FE11	√	√	FE62		√
FE12	√	√	FE64		√
FE13		√	FS1		√
FE14	√	√	FS2	√	√
FE15		√	FS3	√	√
FE18			FS4	√	√
FE20			FS6	√	
FE21		√	FS7	√	
FE27		√	FS8	√	√
FE28			FS10	√	√
FE31		√	FS13	√	√
FE33	√	√	FS19		√
FE37		√	FS20		√
FE39	√	√	FS34		√
FE43	√	√	FS37	√	√
FE44	√	√	FS38	√	√
FE47			FS44	√	√
FE48			FS45		√
FE51		√	FS47		

### Questionnaire design and formulation

#### Design of the questionnaire

The questionnaire is designed to measure the effect of each factor on HWS. The questions were created using simple and understandable language. The questionnaire is organized into two sections. The first section provides details about the questionnaire’s objectives, HWS definition, and the method of response. Then, it includes questions about the participant’s name (optional), job description, and years of experience. The second one presents the 78 factors affecting HWS classified into six categories, as presented before. Participants are asked, based on their experience, to identify the impact of each factor on HWS using a 5-point scale, where one means that the factor has no impact, and five means that the factor has a very strong impact (Table 2).

**Table 2:** Likert scale for determining the impact of factors.

Score	The factor impact
1	No impact
2	Minor impact
3	Moderate impact
4	Strong impact
5	Very strong impact

#### Pilot study

After designing the questionnaire, it was piloted to test the effectiveness and clarity of the selected factors and the questionnaire design. Moreover, the pilot study tests the duration needed for each participant to answer the questions. Bell claimed that the pilot study helps in “getting the bugs out of the instrument”, which aims to resolve issues and avoid difficulties the respondents may confront, in addition to checking the questions’ format^32^.

The pilot study involved 7 experts from the fields of Highway Engineering and Construction Management in Egypt, each with over 15 years of experience. Interviews took place with these experts who provided worthy recommendations related to factors’ classification and reformulation. Their recommendations were incorporated to eliminate any ambiguity and ensure respondents could clearly understand the questionnaire.

#### Launch of the questionnaire

In this study, the population size is undefined as it is difficult to define all the specialists in highway projects accurately. Thus, the sample size of the undefined population can be calculated using Eq. (7)^33^:

1 $$\:n=\:\frac{{Z}^{2}\:x\:P\:(1-P)}{{E}^{2}}\:\:\:\:\:\:\:\:\:\:$$

Where *n* = the minimum sample size; *P* = standard deviation [0.5], *E* = acceptable margin of error [10%], and the confidence level 95% [*Z* = 1.96], and the sample size is determined as:

$$n = \;\frac{{{{1.96}^2}\;x\;0.5\;\left( {1 - 0.5} \right)}}{{{{0.1}^2}}} = 96.04 = 96\;sample$$

Since the developed RS evaluates HWS in Egypt, it was selected for data collection. This was based on Egypt’s status as a leading country in the Middle East for sustainability achievement, as highlighted in the latest UN 2023 report on the overall performance of all 193 UN Member States^34^. Hence, responses were gathered from experts residing in 18 different cities across Egypt. The questionnaire was prepared both electronically (Google Forms) and in hard copies. The Google Forms were sent by e-mail and “LinkedIn”, and interviews were held with Egyptian experts from universities and construction practitioners (owners, contractors, and consultants). Such a survey required significant effort in carefully selecting and communicating with highly experienced practitioners in the study scope to ensure the accuracy of the results obtained. The primary purpose of conducting these interviews was to ensure the reliability of responses and help ensure the interviewee remains focused and keeps on-track to complete the survey. About 240 surveys were distributed, and 135 responses were collected with a response percentage of 56% within a period of 3 months. After reviewing the responses, only 100 surveys were accepted (satisfying the required sample size of 96).

### Data analysis

#### Demographic data analysis

**Fig. 4:**
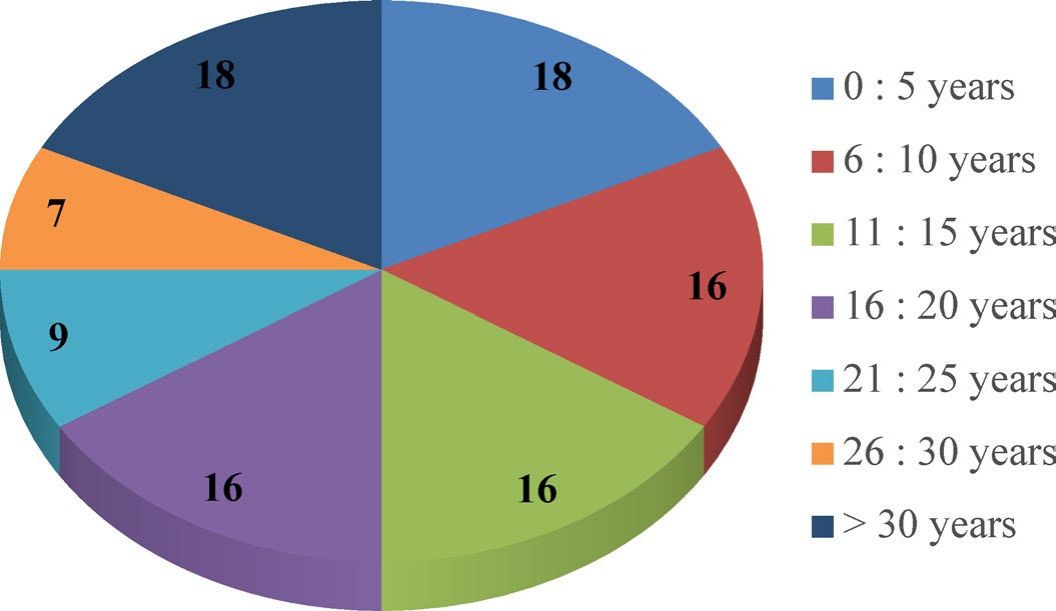
Respondents’ years of experience.

Among the 100 responses received, 43% are academics, from 18 research and educational institutions, and 57% are highway professionals working for contracting, consulting companies, and governmental agencies. These professionals were classified as: 29 site engineers, 15 managers, 7 technical office engineers, and 6 design engineers. The diversity ensures that various perspectives are captured. As shown in Figs. 4 and 66% of the participants have over 10 years of work experience, enriching the reliability of the questionnaire results. The sample selection aimed to represent parties in the highway industry in a developing country like Egypt, including governmental agencies, contractors, consultants, and academics. Table S5 (in the supplementary) shows samples of the survey responses.

#### Reliability and normality testing

In this study, Cronbach’s alpha coefficient, which ranges from 0.0 to 1.0, was used to test the reliability of the selected factors. The closer Cronbach’s coefficient to 1.0 indicates greater the internal consistency. Typically, a Cronbach’s alpha between 0.7 and 1.0 signifies reliable results^35^. The statistical analysis conducted using Minitab reveals a Cronbach’s alpha of 0.954 for the 78 factors, indicating high reliability of the collected data. The reliability testing by calculating Cronbach’s alpha value ensures that responses of the questionnaire are reliable and not provided randomly, indicating that the participants comprehended the questions and responded thoughtfully.

**Fig. 5:**
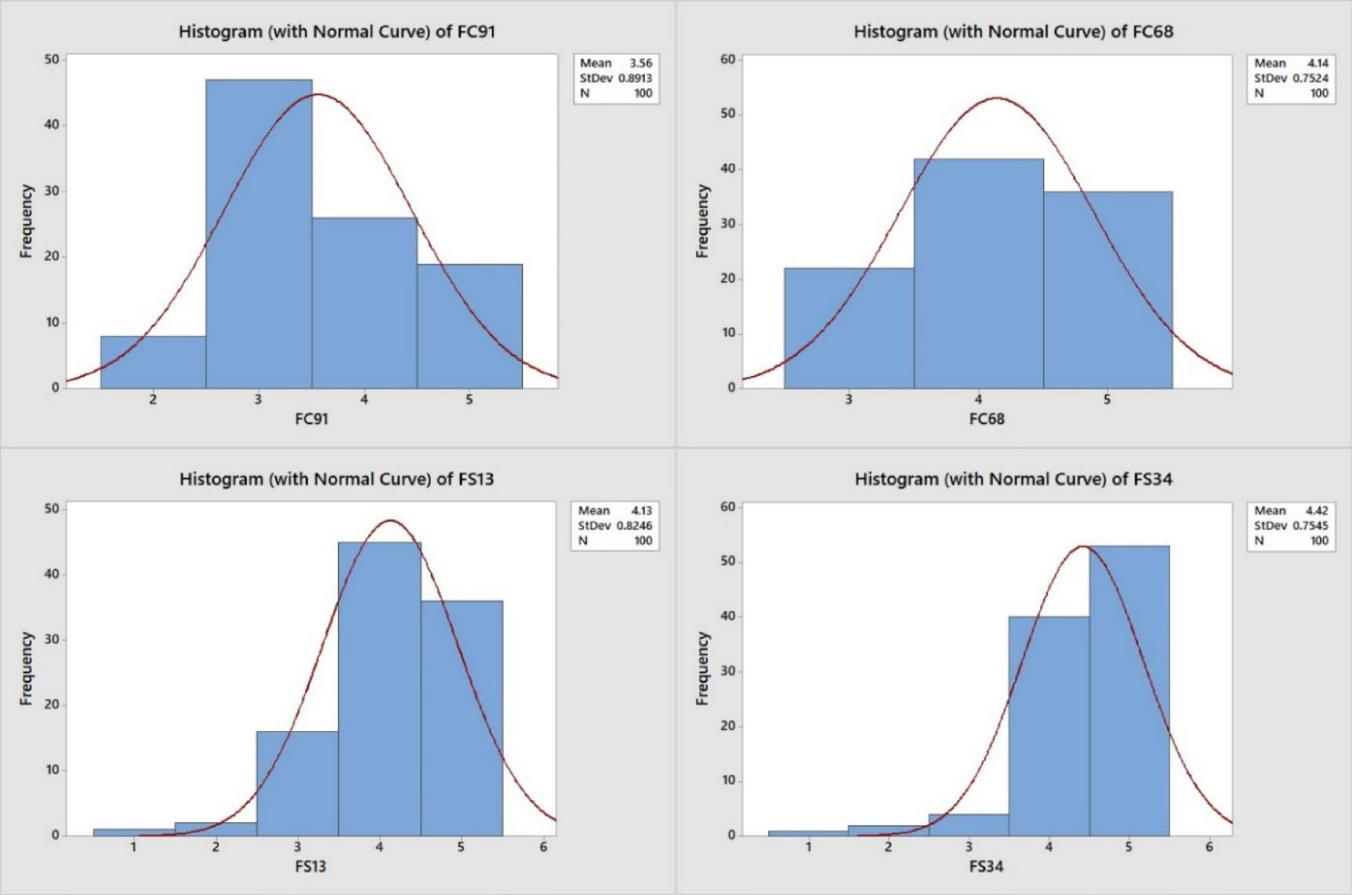
Examples of the normality testing results.

Furthermore, descriptive statistics were performed to calculate (Minimum, Median, Maximum, Mean, Variance, Standard Deviation, Kurtosis, and Skewness) and the test of normality. These statistics refer to the extent to which respondents agree on one answer or the distribution of answers in a way that follows a normal distribution curve. Table S6 (in the supplementary) summarizes the descriptive statistics results. The kurtosis of some factors shows variance among respondents’ answers (e.g., FC91 and FC68), while others have skewed towards the high impact of factors (e.g., FS13 and FS34) (Fig. 5).

#### Relative importance index (RII)

The first step to rank and summarize the collected factors is to determine their relative importance Index (RII), which ranks factors based on the response rate received and the number of responses at that rate, Eq. 2^36^.

2 $$\:RII=\:\frac{\sum\:_{i=1}^{n}{W}_{i}\:.\:{X}_{i}\:}{A\:x\:n}\:x\:100\:\:\:\:\:\:$$

Where *n* = the number of respondents; *W*_*i*_ = the Likert scale levels from 1 to 5, and *X*_*i*_ = the frequency of a selected level; *A* = the highest Likert scale range (i.e., 5). RII value classifies factors into 5 categories as shown in Table 3. Factors ranked as “Medium–Low” and “Low” are omitted, while factors ranked “High”, “High–Medium”, and “Medium” are considered^37,38^.

**Table 3:** Categories of relative importance index (RII).

RII	Category
0.0–0.2	Low (L)
0.2–0.4	Medium-Low (L-M)
0.4–0.6	Medium (M)
0.6–0.8	Medium-High (H-M)
0.8–1.0	High (H)

Results showed that all factors were ultimately considered, as their RII values exceeded 0.6 (Table S7 in the supplementary). Forty-three factors (16 economic, 15 environmental, and 12 social) have RII values ranging from 0.800 to 0.906 and are ranked “High” important factors. Factor FC63 “Design long-life pavement” was ranked first with RII = 0.906. The highest-ranked factor belongs to the economic dimension. On the other hand, a total of 35 factors (16 economic, 14 environmental, and 5 social) are ranked “H–M”. Factor FC1 “Determine the project profitability during the project operation” was ranked the lowest with RII = 0.692. The findings show that respondents did not highly prioritize environmental and social issues. As a result, this approach could not condense the number of factors into a smaller set but only ranks the considered factors and highlights the findings.

#### Principal component analysis (PCA)

The second stage used in this study to identify the most significant factors affecting HWS is to develop a Principal Component Analysis (PCA), which is a statistically popular data analysis technique used to reduce data to a smaller set of summary parameters and to identify the relationship among variables. This technique depends on creating the correlation matrix of survey results and summarizing data based on determining the number of factors that are highly important to be prioritized from a list of factors^38,39^. This analysis was carried out using Minitab, starting by calculating Pearson correlation coefficients between all pairs of factors (correlation matrix) (Eq. 3), neglecting the grouping presented in Fig. 3.

3 $$\:r=\:\frac{\sum\:\left(\right(X-\:{M}_{X}\left)\right((Y-\:{M}_{Y}))}{\sqrt{{(\sum\:(X-\:{M}_{X})}^{2}\left){(\sum\:(Y-\:{M}_{Y})}^{2}\right)}}\:\:\:\:$$

As *X* = values of factor *i*; *Y* = values of factor *j*, and *M*_*x*_ = mean of factor *i* values; *M*_*y*_ = mean of factor *j* values. The correlation coefficients range between − 1 and + 1, as + 1 means an ideal positive linear correlation, and − 1 means a perfect negative correlation. A threshold was set to select only correlations greater than 0.3 and less than 0.9^37^. Factors with correlation coefficients more than 0.9 indicate that they are very highly correlated, while those less than 0.3 have little correlation^40^. The aim is to determine factors with moderate and high correlations as a base for prediction according to international practices^41^. Subsequently, factors that had a correlation coefficient of more than 0.9 or less than 0.3 were omitted.

**Fig. 6:**
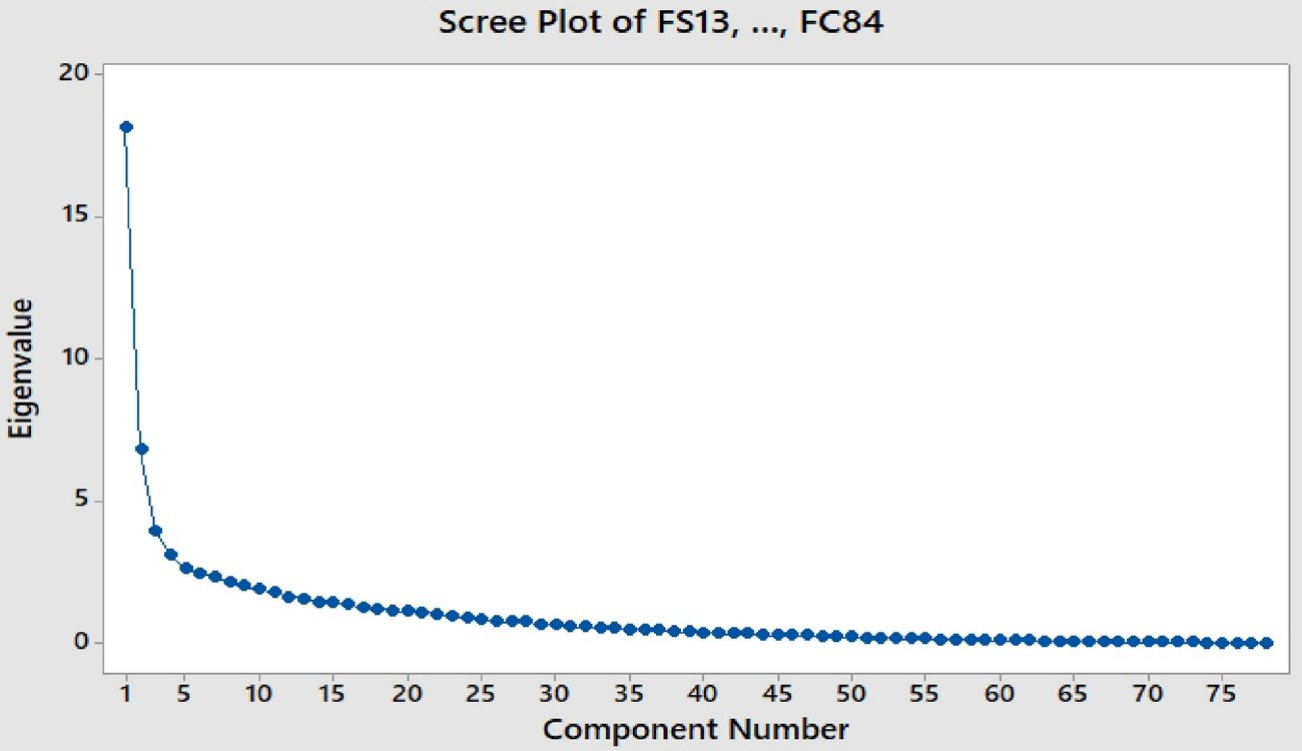
The Scree plot of the analyzed factors.

After developing the factor analysis, the Scree test clarifies the cutoff point determined by analyzing the shape of the resulting curve. This point is determined by plotting the latent roots against the number of factors in their order of extraction. Figure 6 shows the Scree plot of the factors. Starting with the first factor, the plot slopes steeply downward at first, becoming gradually almost a horizontal line. The maximum number of components to extract is indicated by where the curve first starts to flatten out. As shown, the first 17 factors would be qualified as they have a threshold of eigenvalues to be more than 1.0, which represents the amount of variance accounted for the factor and determines the component number of the most significant factors resulting from the correlation^42^. Beyond 17, these factors would be excluded.

For the 1 st correlation iteration, a correlation matrix was developed with all the 78 factors and 46 factors were omitted (i.e., FS13, FS19, FS47, FC3, FE56, FC89, FC91, FS1, FS4, FS7, FS37, FS38, FS45, FC8, FC12, FC57, FE20, FE21, FE47, FE48, FC68, FE15, FC15, FE14, FE64, FS3, FE1, FC58, FE7, FE12, FE51, FC1, FC4, FC38, FC42, FC63, FC64, FC18, FC66, FC67, FC71, FC32, FC2, FC5, FC56, and FC84), as the factor can be neglected when the most correlation coefficients between it and other factors are out of range (i.e., less than 0.3 or more than 0.9). Table 4 shows a part of the correlation matrix between factors for the 1 st correlation iteration.

To ensure that the remaining factors meet the threshold, another iteration was conducted to make sure that the remaining factors have sufficient relationships and high effects on the HWS. After conducting the 2nd iteration with the remaining 32 factors, 20 factors were omitted (i.e., FS2, FC70, FE31, FC14, FE43, FE27, FC78, FE2, FE5, FE13, FE33, FS34, FC37, FS10, FS44, FS6, FS20, FC6, FC11, and FC16). For more accuracy, a third iteration was carried out, and factor FE37 (i.e., minimize aggregate transportation to reduce congestion and emissions) was omitted.

**Table 4:** A part of the correlation matrix between factors for the 1 st iteration.

	FS13	FS19	FS47	FC3	FE56	FC89	FC91	FS1	FS2	FS4	FS7	FS37	FS38	FS45
FS13	1.00													
FS19	0.50	1.00												
FS47	0.14	0.35	1.00											
FC3	0.31	0.24	0.22	1.00										
FE56	0.21	0.23	0.19	0.19	1.00									
FC89	0.13	0.06	0.28	0.04	0.13	1.00								
FC91	0.11	0.21	0.19	0.24	0.14	−0.04	1.00							
FS1	0.29	0.12	0.18	0.35	−0.04	0.25	0.14	1.00						
FS2	0.29	0.07	0.23	0.21	0.11	0.25	0.16	0.33	1.00					
FS4	0.39	0.24	0.13	0.44	0.26	0.04	0.26	0.27	0.34	1.00				
FS7	0.07	0.19	0.20	0.18	−0.06	0.06	0.30	0.31	0.32	0.28	1.00			
FS37	0.22	0.15	0.18	0.00	−0.12	0.22	0.21	0.23	0.37	0.20	0.20	1.00		
FS38	0.10	0.08	0.29	0.21	0.09	0.07	0.39	0.24	0.37	0.16	0.25	0.13	1.00	
FS45	0.23	0.09	0.40	0.30	0.16	0.19	0.31	0.30	0.51	0.32	0.39	0.35	0.51	1.00

To get more accurate results, a fourth iteration was carried out to ensure that no more data exceeded the thresholds. After eliminating the excluded factors, a list of 11 factors remains with 9 environmental factors (i.e., FE11, FE18, FE28, FE39, FE44, FE53, FE57, FE60, and FE62), one social factor (i.e., FS8), and one economic factor (FC10) (Table S8 in the supplementary).

After conducting PCA and assigning the selected factors to their related categories and sub-categories, it was noticed that some categories and sub-categories were excluded due to the elimination of their corresponding factors (61 factors were eliminated). For instance, the categories of “Quality of life” and “Structure” were completely omitted, and all factors related to energy and water sub-categories were, unexpectedly, eliminated. This exclusion affected the reliability of results and limited the representation of factors affecting HWS. To address this issue, it was decided to apply other statistical techniques to reduce the number of factors further and develop a rating system that included the most significant factors affecting HWS.

As shown, the results of the PCA refer to the necessity of providing educational and awareness programs to highway professionals to broaden their knowledge in HWS and enable them to apply sustainability through the highway project life cycle. On the other hand, the clustering performed in the PCA enabled the authors to identify the gap between the questionnaire’s objective and the respondents’ awareness of HWS. This gap limited the concept of sustainability to the environmental aspect only (i.e., 9 factors out of 11). Moreover, the reason behind the disappearances of some categories may be in part due to the attitude of the respondents in limiting the concept of sustainability to the environmental factors only, while ignoring other categories. Identifying this limitation was a crucial step to assess the credibility of the respondents’ answers independently of the Cronbach’s alpha value. Regarding the use of sustainability dimensions in the model categorization, it was found to be the simplest method for assigning factors to their most relevant aspects. This approach also empowers decision-makers to evaluate the sustainability performance of each dimension separately, allowing them to pinpoint the underperforming aspect that negatively affects overall sustainability performance. Additionally, it was necessary to have all categories represented in the proposed evaluation system.

#### The Pareto principle (20/80 Rule)

In general, it is difficult to develop an applicable model to evaluate HWS with 78 measuring factors. As demonstrated, the sequential application of statistical techniques was essential for summarizing the data, as no single step alone could adequately reduce the number of factors. Conversely, if any statistical technique had been able to condense the data into a manageable number of factors, additional analyses would not have been necessary. Furthermore, the correlations eliminated essential factors necessary for assessing HWS comprehensively. Hence, it is decided to use the Pareto principle, stating that only 20% of a phenomenon’s causes may express 80% of its effects^43^. Depending on RII values, all factors were arranged in descending order in each category.

**Fig. 7:**
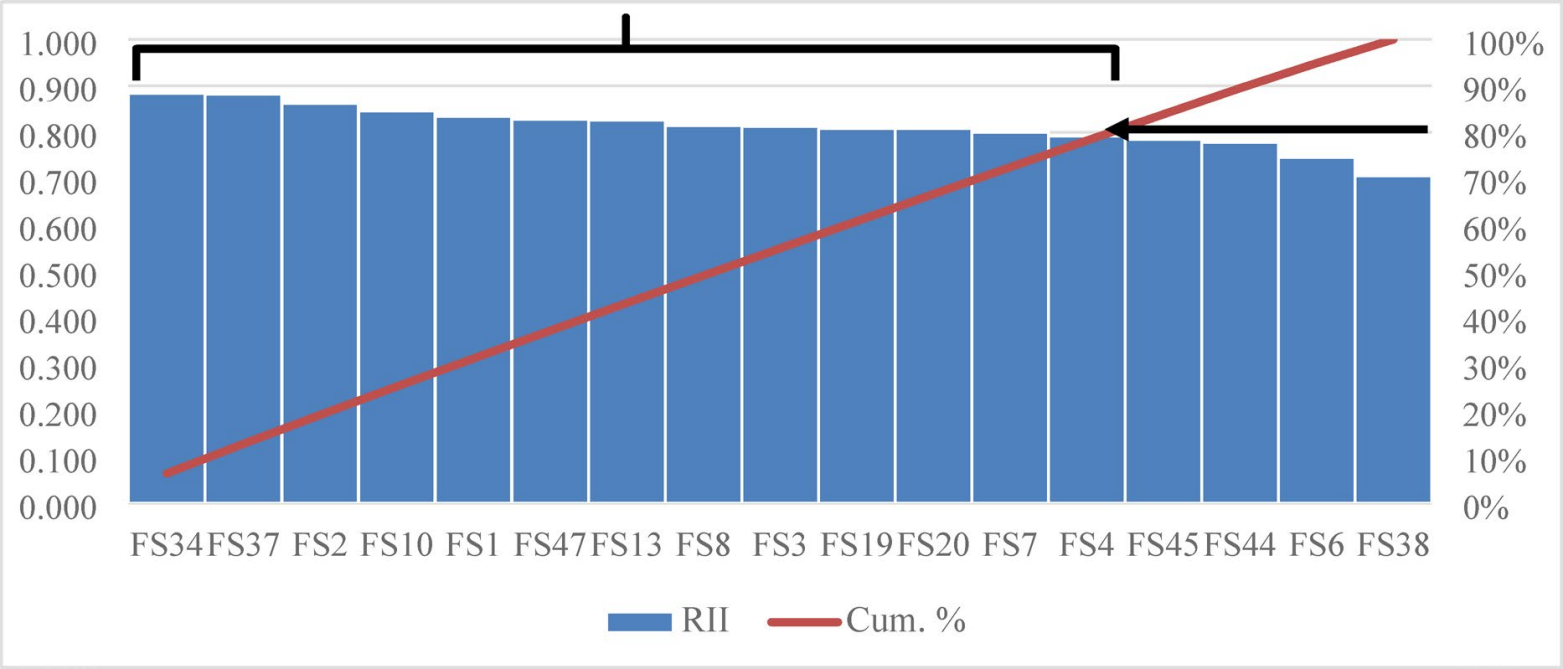
Pareto chart for social factors.

**Fig. 8:**
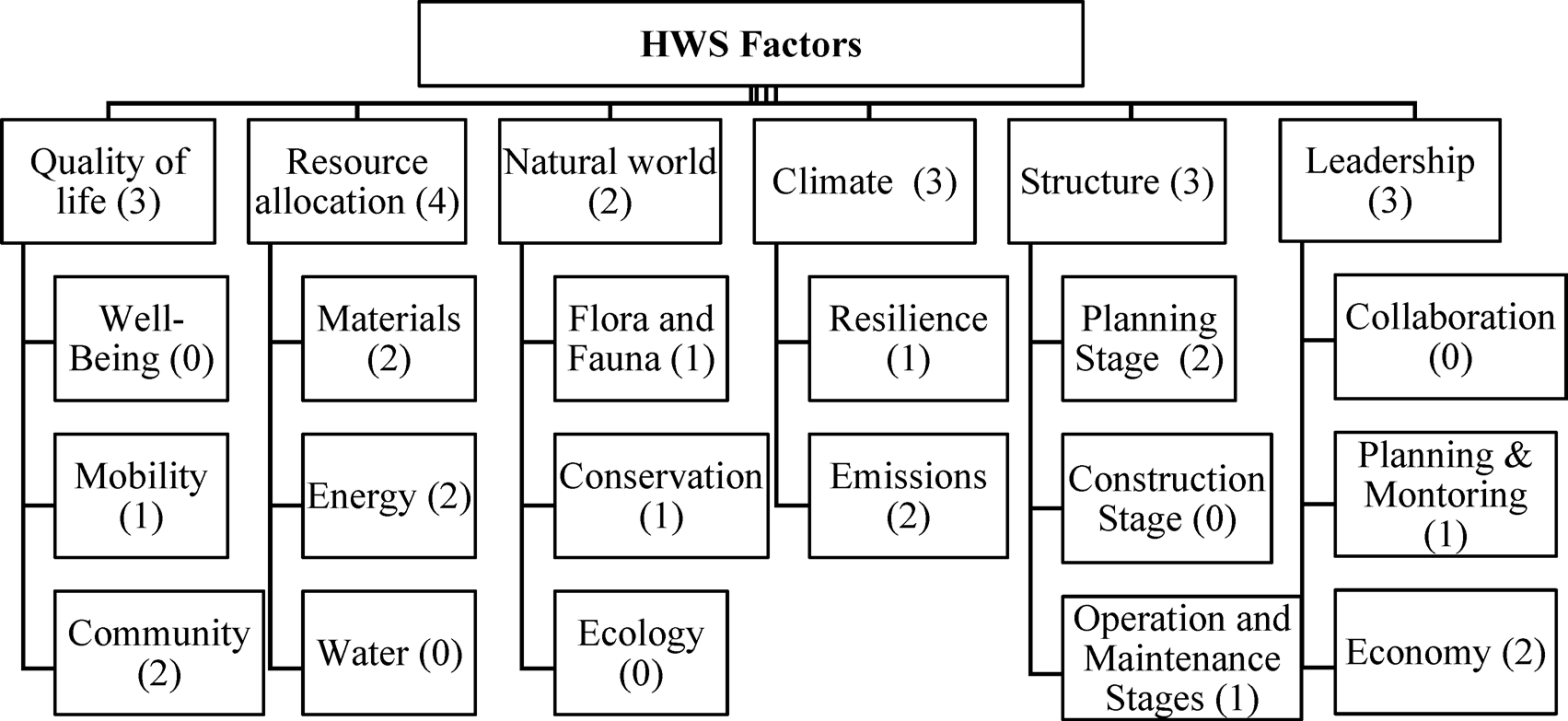
The final breakdown structure of the selected factors.

Applying such a principle, 62 factors (28 environmental, 14 social, and 25 economic) out of 78 were selected based on the cumulative percentage of more than 80% due to the convergence of RII values (Fig. 7, Table S9 in the supplementary). These 62 factors make the development of an AHP survey a complex process. Therefore, it was decided to select only the top 20% of factors in each category, which resulted in 18 factors (5 environmental, 3 social, and 10 economic). Figure 8 shows the new distribution of factors in each category and sub-category, which clarifies the fair representation for all categories and most of the sub-categories of the suggested clustering criteria. Table 5 lists the final factors that would be used to develop the HWS RS through the sustainability aspects, as the resulted factors are clustered through the triple-bottom line of sustainability. This clustering helps evaluate the sustainability performance within each aspect and determines the shortcomings in a certain aspect that need to be avoided. Moreover, the resulting factors would then be evaluated by experts to ensure their ability to effectively and comprehensively evaluate the sustainability performance of a given highway project.

**Table 5:** Final factors due to selecting the top 20% factors.

Category	ID	Factor
Social	FS2	Improve local infrastructure capacity (roads) and ensure proper services and infrastructure for all
	FS3	Select a suitable/undeveloped site for the project, which is utilized effectively
	FS37	Provide access to safe and sustainable transportation systems for all, including enhancement of road and drivers’ safety, and encourage carpooling and bicycle
Environmental	FE1	Apply life cycle assessment
	FE5	Examining and reducing potential air and water pollution from the project and its impact on the local climate
	FE15	Saving energy and resources consumption through the project life cycle
	FE33	Use recycled materials to reduce wastes
	FE39	Provide and increase access to safe, green and public spaces for all, and confront desertification, in addition to restoring degraded land and soil
Economic	FC5	Develop a feasibility study to define the capital budget needed for the project
	FC8	Selecting economic, durable, and available materials
	FC14	Reduce the consumption of various types of energy, especially fossil fuel, and encourage using renewable energy and define their costs
	FC32	Develop a clear and detailed program for quality management including developing a quality control plan (QCP)
	FC42	Design pavement according to the regional conditions according to the soil type and traffic volume
	FC63	Design long-life pavement
	FC70	Create, modify, and use specifications that allow for sustainability best practices and achieve the efficient use of materials
	FC71	Plan for long-term monitoring and maintenance
	FC84	Develop life-Cycle Cost Analysis (LCCA) and Benefit‐Cost Analysis (BCA) to apply the best alternatives
	FC89	Accommodate multi-modal transportation uses (freight vehicles, pedestrians, ridesharing, and bicyclists), including providing new intermodal connections

### Analytical hierarchy process (AHP)

Unlike other multi-criteria decision-making strategies, the AHP technique can measure intangible criteria, as it does not always require a perceptible numerical scale or ratio^44^. Additionally, it features a consistency check to ensure the transitivity of judgment enforcement. The first step in the AHP’s decision-making process involves breaking down the problem into a hierarchy of issues to be considered. These hierarchical levels help streamline the problem’s illustration, making it more understandable. The elements’ weights at each hierarchical level are then determined^45^.

**Fig. 9:**
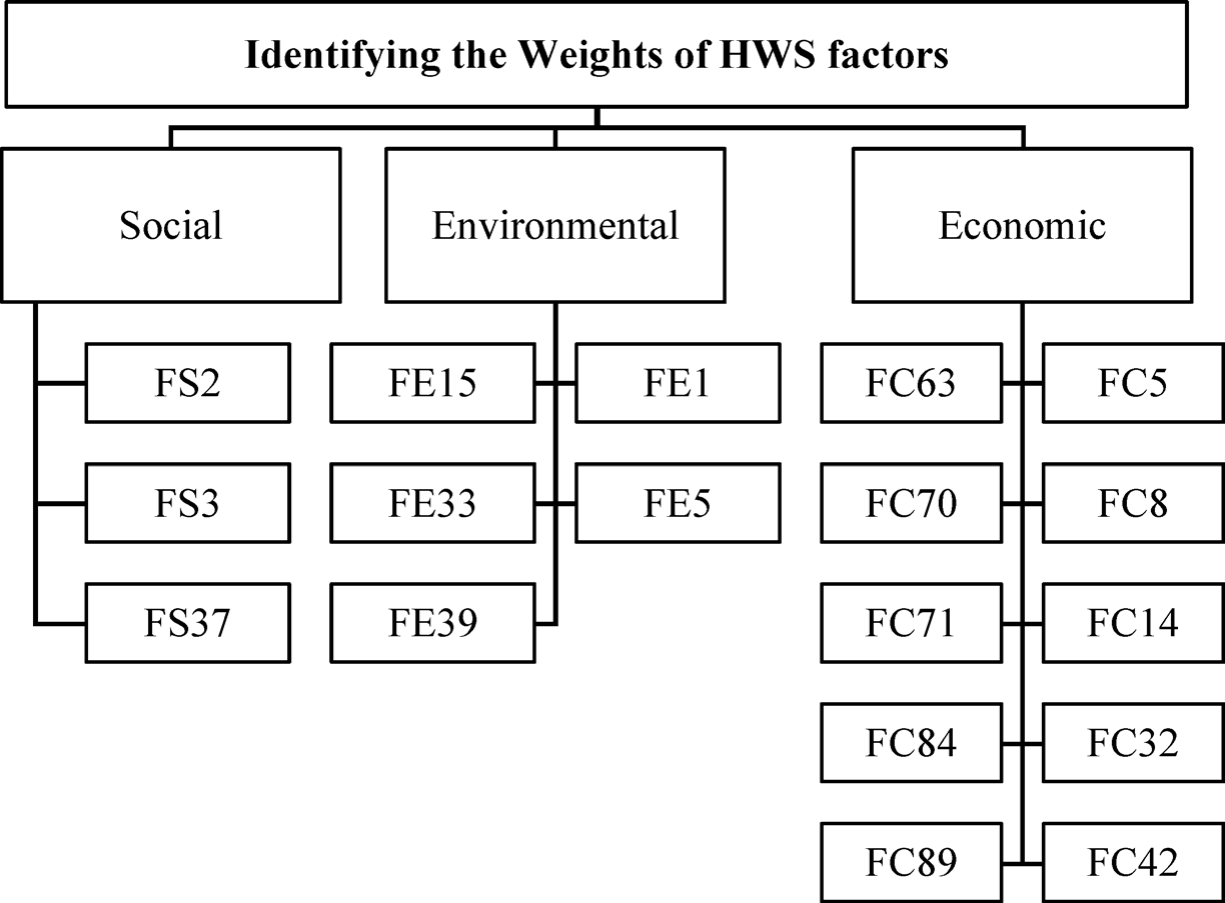
The hierarchy structure of HWS factors through the AHP survey.

This technique was used to determine the relative weights of the selected 18 factors and the relative weights of the sustainability pillars^46^. A hierarchy of 3 levels was developed (Fig. 9). The top level represents the main goal (i.e., identifying the weights of HWS factors). Level 2 consists of the sustainability pillars, while the third level refers to the HWS factors.

#### Development of pair-wise comparison matrices

In this study, three pair-wise comparison matrices were developed for social, environmental, and economic factors (Factors matrix) with sizes 3 × 3, 5 × 5, and 10 × 10, respectively. Another 3 × 3 matrix was built for the three main pillars of sustainability (Aspect matrix). The preference element is quantified using a nine-point scale shown in Table 6.

**Table 6:** Saaty’s scale for pair-wise comparison to define the relative importance of two criteria.

Importance Intensity	Definition	Explanation
1	Equal importance	Two activities contribute equally to the objective
3	Moderate importance	Experience and judgment slightly favor one factor over another
5	Strong importance	Experience and judgment strongly favor one factor over another
7	Very strong importance	A factor is favored very strongly over another
9	Extreme importance	The evidence favoring one factor over another is of the highest possible order of affirmation
2, 4, 6, 8	Intermediate values	When compromise is needed

#### Demographic data for the AHP respondents

**Fig. 10:**
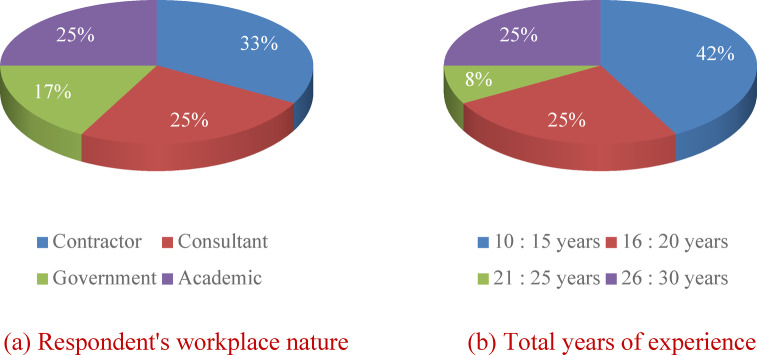
Descriptive analysis of the 12 interviewed experts.

The respondents for this study were carefully selected to include Egyptian experts with at least 10 years of experience in the pavement construction industry. These professionals were drawn from various sectors, including contracting and consulting companies, as well as governmental agencies. Their roles encompassed a broad spectrum of expertise, including design engineers, site engineers, and project managers involved in highway projects. This targeted selection ensured a comprehensive perspective on the subject matter. The AHP survey was sent by e-mail, in addition to helding face-to-face interviews. Accordingly, twenty Egyptian experts were contacted, but only fourteen agreed to participate in the survey. Moreover, two respondents were rejected due to invalid and inconsistent information. 50% of those experts have both academic and engineering expertise and have relevant research publications in the highways and sustainability scope. Six respondents work for contracting and consulting companies, as well as governmental agencies, in roles such as design engineers, site engineers, and project managers for highway projects (Fig. 10.a). More than 58% of participants had work experience of 16 years or more, which increases the questionnaire’s reliability (Fig. 10.b). Generally, the more questionnaires that are performed, the better the results that can be admitted^47^. However, in earlier relevant studies using the AHP, the number of participating experts is usually low (e.g., 9 experts)^48^. In this study, respondents were identified based on their experience (i.e., more than 10 years)^49^.

#### AHP analysis

Four pair-wise comparison matrices were developed by calculating the geometric average of the respondents’ answers and carrying out the column normalization (Tables S10 – S13 in the supplementary). The factors were grouped due to the sustainability aspect to create three pair-wise comparison matrices, and the main sustainability aspects were arranged to create the pair-wise comparison to determine the relative importance of each aspect. The analysis of the AHP survey is carried out in two steps. First, the pair-wise comparison matrices are normalized to get the column vector (Eq. 4). Then, the priority vectors *(PVs)* are calculated, which express the relative weight of each factor (Eq. 7).

4 $$\:{a}_{ij}^{\:\:*}=\:\frac{{a}_{ij}}{\sum\:_{i=1}^{n}{a}_{ij}}\:\:\:\:\:\:\:\:\:\:\:\:\:\:\:\:\mathrm{f}\mathrm{o}\mathrm{r}\:\mathrm{e}\mathrm{a}\mathrm{c}\mathrm{h}\:\mathrm{j}1,\:2,\:\dots\:\dots\:.,\:\mathrm{n}\:\:\:\:\:\:$$

5 $$\:{w}_{ij}=\:\frac{\sum\:_{j=1}^{n}{a}_{ij}^{\:\:*}}{n}\:\:\:\:\:\:\:\:\:\:\:\:\:\:\:\:\:\:\:\:\:\:\:\:\mathrm{f}\mathrm{o}\mathrm{r}\:\mathrm{e}\mathrm{a}\mathrm{c}\mathrm{h}\:\mathrm{i}=1,\:2,\:\dots\:\dots\:.,\:\mathrm{n}\:\:\:\:\:\:\:$$

where $\:{a}_{ij}^{\:\:\mathrm{*}}$= the normalized value for each pair of factors; $\:{a}_{ij}$= the relative weight for each pair of factors, and *n* = number of factors in each matrix; $\:{w}_{ij}$= the priority vector for each factor. Subsequently, a check of consistency was carried out by measuring a consistency ratio (*CR*). As a first step, each matrix is multiplied by the *PV* (*W*) to obtain *W’*. The second step is to divide *W’* by W to obtain *W”*, then (*λ*_*m*_) and (*CI*) are calculated (Eq. 6 and Eq. 7):

6 $$\:{{\uplambda\:}}_{m}=\:\frac{\sum\:\frac{w{\prime\:}}{PV}}{n}\:\:$$

7 $$\:CI=\:\frac{{({\uplambda\:}}_{m}-n)}{n-1}\:\:\:\:\:$$

The consistency ratio is calculated by dividing the consistency index (*CI*) by the random index (*RI*). The *RI* depends on the number of parameters being compared (i.e., the number of factors in each matrix) (Table 7)^50^. To accept the consistency of a given judgment, *CI* should be less than 0.1. The *CI* was calculated for each respondent to exclude the inconsistent responses (in this case, the responses of 2 participants were excluded). After that, *CI* was determined for the geometrical average matrices of the remaining respondents, which were less than 10%, providing an acceptance for the AHP survey results.

**Table 7:** Random index^50^.

No. of parameters	1	2	3	4	5	6	7	8	9	10
RI	0.00	0.00	0.58	0.9	1.12	1.24	1.32	1.41	1.45	1.49

**Fig. 11:**
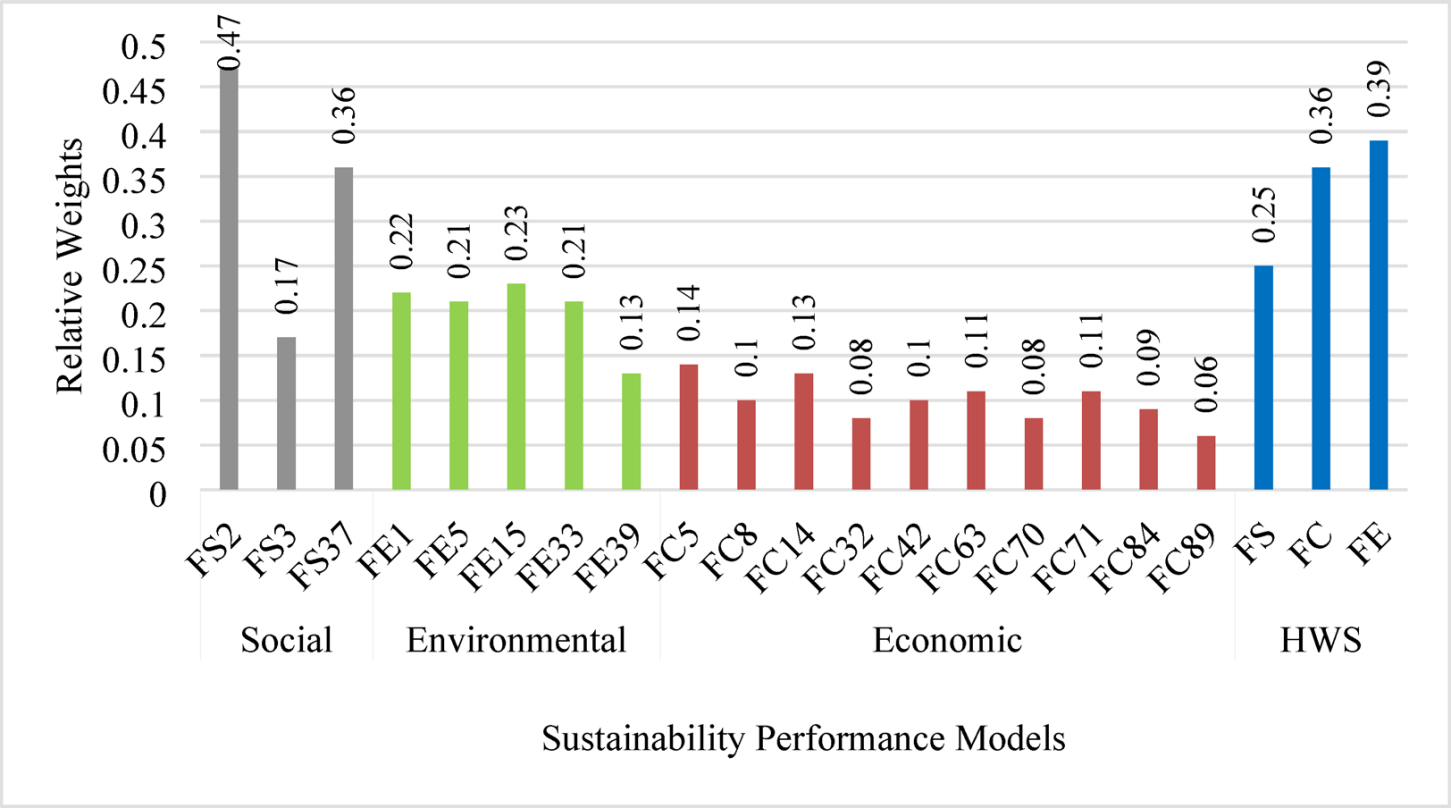
Priority values of factors and main pillars of HWS.

For social factors, Fig. 11 shows that factor FS2 “Improving the local infrastructure capacity (i.e., roads) and ensuring proper services and infrastructure for all” has the greatest *PV* of 0.47, while factor FS3 “Selecting a suitable/undeveloped site for the project, which is utilized effectively” has the lowest *PV* of 0.17. A check of consistency reveals an acceptable *CR* of 0.3%.

Subsequently, the AHP analysis was carried out for the environmental factors (Fig. 11). The findings referred to factor FE15 “Saving energy and resources consumption through the project life cycle” as the most important factor with *PV* of 0.23, while the least important factor was FE39 “Providing access to safe, green, and public spaces for all, and confront desertification, in addition to restoring degraded land and soil” with *PV* of 0.13. A consistency ratio of 1.6% was calculated, which meets the minimum threshold.

Also, the analysis was applied to the economic factors. The highest *PV* of 0.14 was given to factor FC5 “Developing a feasibility study to define the capital budget needed”, but the lowest *PV* of 0.06 was given to factor FC89 “Accommodating multi-modal transportation uses (freight vehicles, pedestrians, ridesharing, and bicyclists) including providing intermodal connections” (Fig. 11). The *PV* of the other eight factors ranges between 0.09 and 0.13. A consistency ratio of 1.3% was determined, which is smaller than the specified critical value of 10%.

Finally, the relative weights of the main pillars of sustainability were determined. They were ranked as environmental, economic, and social with *PV* values of 0.39, 0.36, and 0.25, respectively (Fig. 11). A *CR* of 5.5% provides an acceptable reliability of the resulted outcomes.

## Results and discussion

### Mapping of HWS RS with SDGs and other infrastructure RSs

After analyzing the investigated infrastructure RSs, the most significant factors of the HWS RS are compared to those of other international RSs. This comparison is summarized in Table 8. The comparison emphasizes the importance of this study, as the recommended factors are not fully included in all the published infrastructure RSs. This highlights the importance and the comprehension of the developed RS, considering the local conditions and practices of highway construction in Egypt. On the other hand, the statistical analyses carried out with the followed sequence aim to construct an RS with a holistic, summarized, and highly relevant set of factors to simplify the determination of HWS scoring. In addition, considering the SDGs and their indicators significantly distinguishes the developed RS from other initiatives reviewed in the literature.

**Table 8:** Comparison of the HWS RS, SDGs, and other infrastructure RSs.

Factor	HWS	AGIC	BE2ST	Envision	GreenLITES Design	GreenLITES O&M	Greenpave	Greenroads	I-LAST	Invest	LEED V4.0	LEED V4.1	Stars	EIA	SDGs
FS2	√	√		√				√						√	√
FS3	√			√	√				√	√		√		√	√
FS37	√		√	√					√	√			√	√	√
FE1	√		√												
FE5	√			√										√	√
FE15	√		√	√		√	√			√		√			
FE33	√		√	√			√		√	√	√				√
FE39	√		√	√		√		√	√			√		√	√
FC5	√									√					
FC8	√									√					
FC14	√							√		√		√			√
FC32	√									√					
FC42	√	√			√										
FC63	√			√				√	√	√					
FC70	√									√					√
FC71	√	√								√					
FC84	√			√						√					
FC89	√				√			√	√	√					

### Developing the assessment questions and scale

A scale was suggested for each factor to help decision-makers evaluate the sustainability performance of a given highway project. A set of questions has been specified for each factor through the sustainability pillars. These questions were obtained from the 13 analyzed infrastructure RSs that were presented in Sect. 2. For instance, the developed questionnaire survey evaluates a factor “Examining and reducing potential air and water pollution from the project and its impact on the local climate”, including evaluating air and water pollution. The aim of merging these sections is to reduce the number of evaluated factors that have the same context, making it easier for respondents to complete the survey. For this factor, it has been broken down into 3 specific questions within the model to measure the distinct value of each component.

A questionnaire survey was developed to determine the relative weights of these questions to define their scores. It was prepared on Google Forms for easy online submission and also in hard copy for interviews with highway experts in Egypt. Twelve experts with at least 10 years of experience were contacted, five of whom have over 30 years of experience. Table S14 (in the supplementary) shows the results of the questions’ weights, indicating that each question has a different weight based on its relative importance. The RII was calculated for each question (Eq. 2), and the weight for each was determined (Eq. 8).

8 $$\:{QW}_{i}=\frac{{RII}_{i}}{\sum\:_{i=1}^{n}{RII}_{i}}\:x\:100$$

Where *QW*_*i*_ = the question weight; *RII*_*i*_ = the relative importance index for question *i*, and *n* = the number of questions below each factor. Hence, each question’s weight is multiplied by 10 grades to obtain its score, which means that each question has a different score.

### HWS assessment model

Based on the previous discussion, the sustainability performance of each aspect (economic, environmental, and social sustainability) can be calculated. Each aspect could be presented by an equation, which assesses its sustainability performance. The inputs are the previously determined factors, in addition to the priority value assigned to each factor that reflects the relative importance of its impact on the expected sustainability outcomes, in addition to the weights of the factors’ questions.

The output is the expected sustainability percentage of the proposed project in each sustainability aspect. Such a percentage ranges from a minimum value of 0.0% to a maximum value of 100%, as each factor has an available score of 10 points. These scores are distributed according to the included questions’ weights, and all the factors’ scores are subjected to the weights of *priority values* to obtain the sustainability score of each aspect out of 10 points. Subsequently, the score of each aspect is subjected to their weights of *priority values* and multiplied by 10 to obtain the sustainability percentage of a given project (Eq. 9), as *s*,* t*,* and u* are the relative weights of the social, environmental, and economic sustainability aspects (i.e., 0.25, 0.36, and 0.39), respectively, and *S*,* E*,* and C* are the sustainability scores for each aspect. The low percentage indicates poor sustainability performance, and vice versa.

9 $$\:HWS=\left[s\:S+t\:E+u\:C\right]\:x\:10=\left[0.25\:S+0.39\:E+0.36\:C\right]\:x\:10$$

**Fig. 12:**
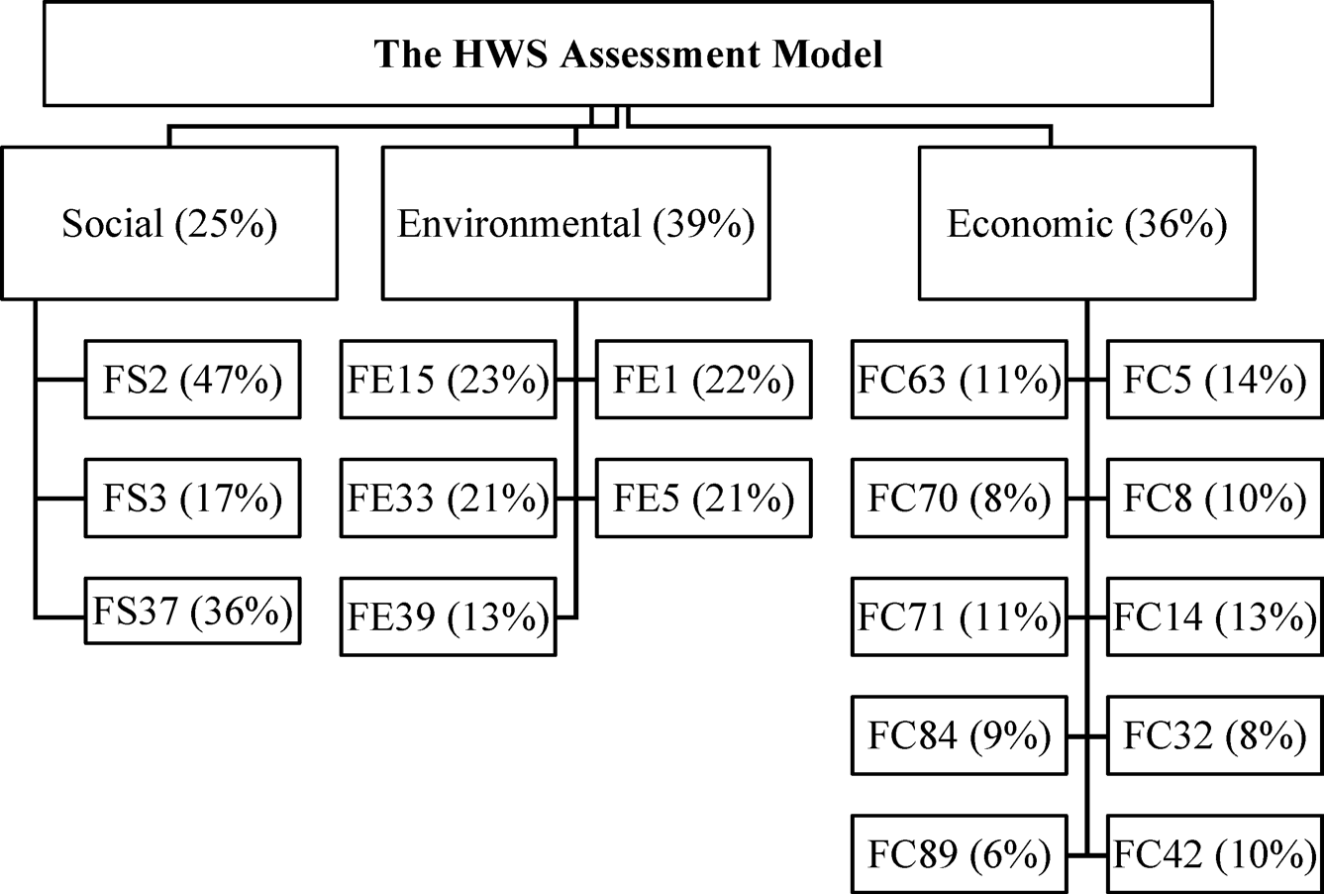
The framework of the HWS assessment model.

As a result, the developed HWS assessment model, including its factors and relative weights, is presented in Fig. 12. Moreover, the HWS is ranked according to the evaluation system used by the Envision RS, which includes five certification levels: declined (HWS < 20%), certified (HWS: 20% − 29%), silver (HWS: 30% − 39%), gold (HWS: 40% − 49%), and platinum (HWS: 50% or higher). As such, a highway project can be evaluated, ranked, and prioritized for implementation.

.

Initially, the user is prompted to specify the highway type (urban or rural) and the project development stage (construction, or maintenance and rehabilitation), as certain questions are relevant only to specific highway types and development stages. The user then answers the questions for each factor to obtain its score. Considering the road type and the project development stage, each question had its weight due to the questionnaire developed, and each factor had its weight due to the AHP analysis. The questions’ scores are summed up to calculate the score of a given factor out of 10. Then factors’ scores are multiplied by their relative weights to calculate a given aspect score. Finally, the project’s overall score is calculated by multiplying aspects’ scores by their relative weights, and the result is multiplied by 10 to represent the HWS performance as a percentage of 100.

### HWS automation and implementation

To facilitate the use of the developed HWS assessment model, it has been coded and implemented using Microsoft Excel. The application consists of three main stages: (1) the inputs, (2) the processing, and (3) the outputs. The inputs of the model are the users’ answers for each question listed for each factor. The processing stage depends on the user’s answers to each question listed for each weighted factor. The outputs are the social, environmental, and economic sustainability scores, in addition to the overall sustainability score of the proposed highway project. The developed RS model is shown in Table S15 (in the supplementary).

### Case study implementation

**Fig. 13:**
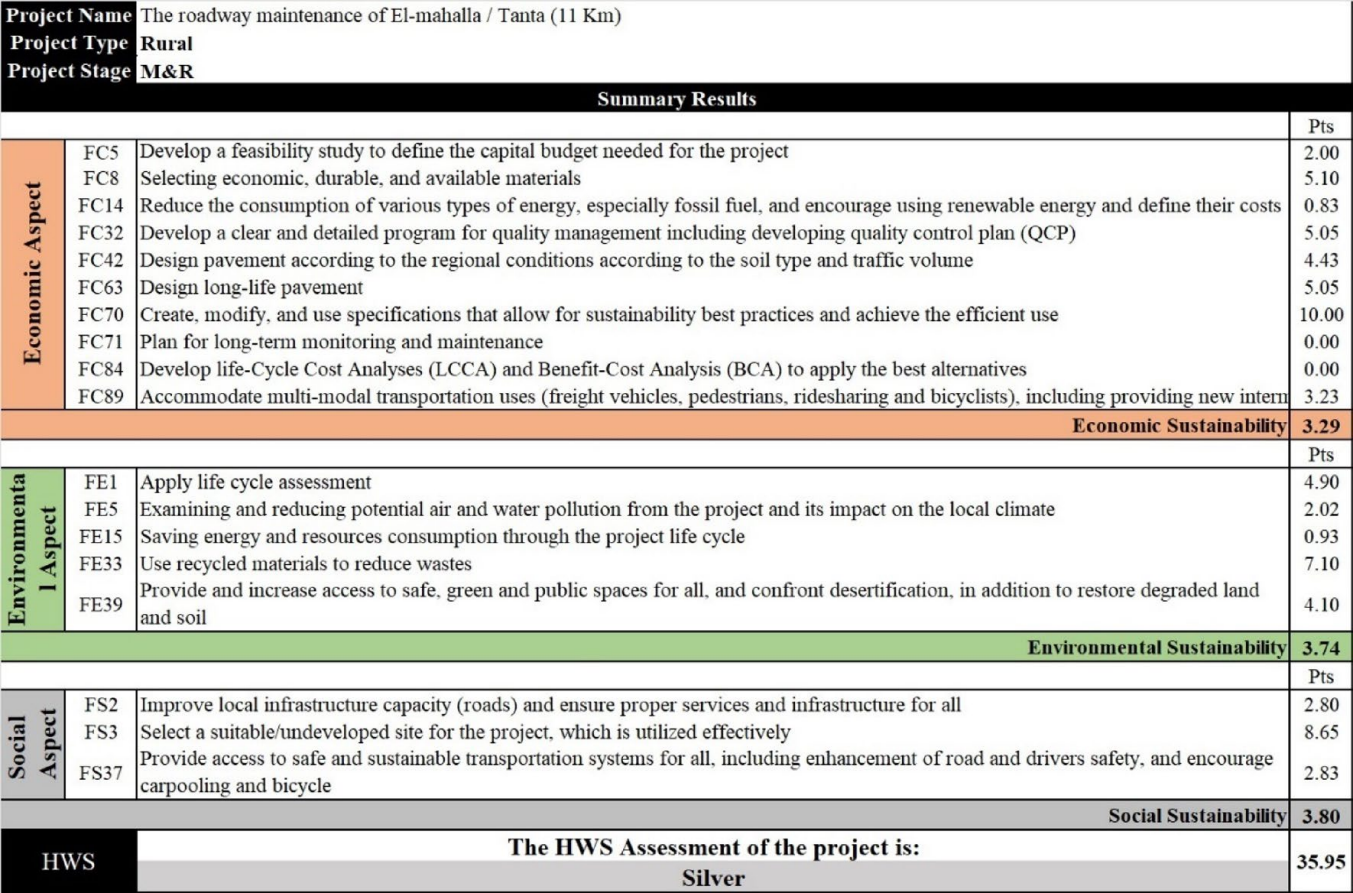
The sustainability performance for case study (1) through the HWS RS.

To ensure the applicability of the developed model, data from two real-life projects were obtained. This step aims to assess the project’s sustainability performance. The first project is an 11 km rural highway connecting El-Mahalla and Tanta cities in the Delta region of Egypt, with bidding focused solely on maintenance activities. The road suffers from major distresses, such as fatigue cracking and rutting, and it was decided to use the old asphalt layers in the rehabilitation stage by using the Full Depth Recycling technique (FDR). Hence, responses to the model questions were gathered from the project site (Table S16 in the supplementary). Figure 13 shows that the project’s sustainability performance level is 32.69% (Silver). This means that the project processes need to greatly incorporate sustainability practices during maintenance activities. To verify the developed model, the case study was applied to another international RS, Envision, which is widely recognized for evaluating sustainability in infrastructure projects. Envision was chosen for comparison due to its online checklist for automatic evaluation and its simplicity, compared to other RSs. Furthermore, Envision considers the largest number of factors existing in the developed RS, following the Invest RS, which does not have an online checklist, making the evaluation difficult. As well, it is noticeable that there are matched factors between SDGs and Envision RS in terms of sustainability principles, especially in the social and environmental aspects (FS2, FS3, FS37, FE5, FE33, and FE39). All the project data was entered through the online checklist, the project scored 226 out of 628 points (35.98%, Silver sustainability level) (Table S17 in the supplementary).

**Fig. 14:**
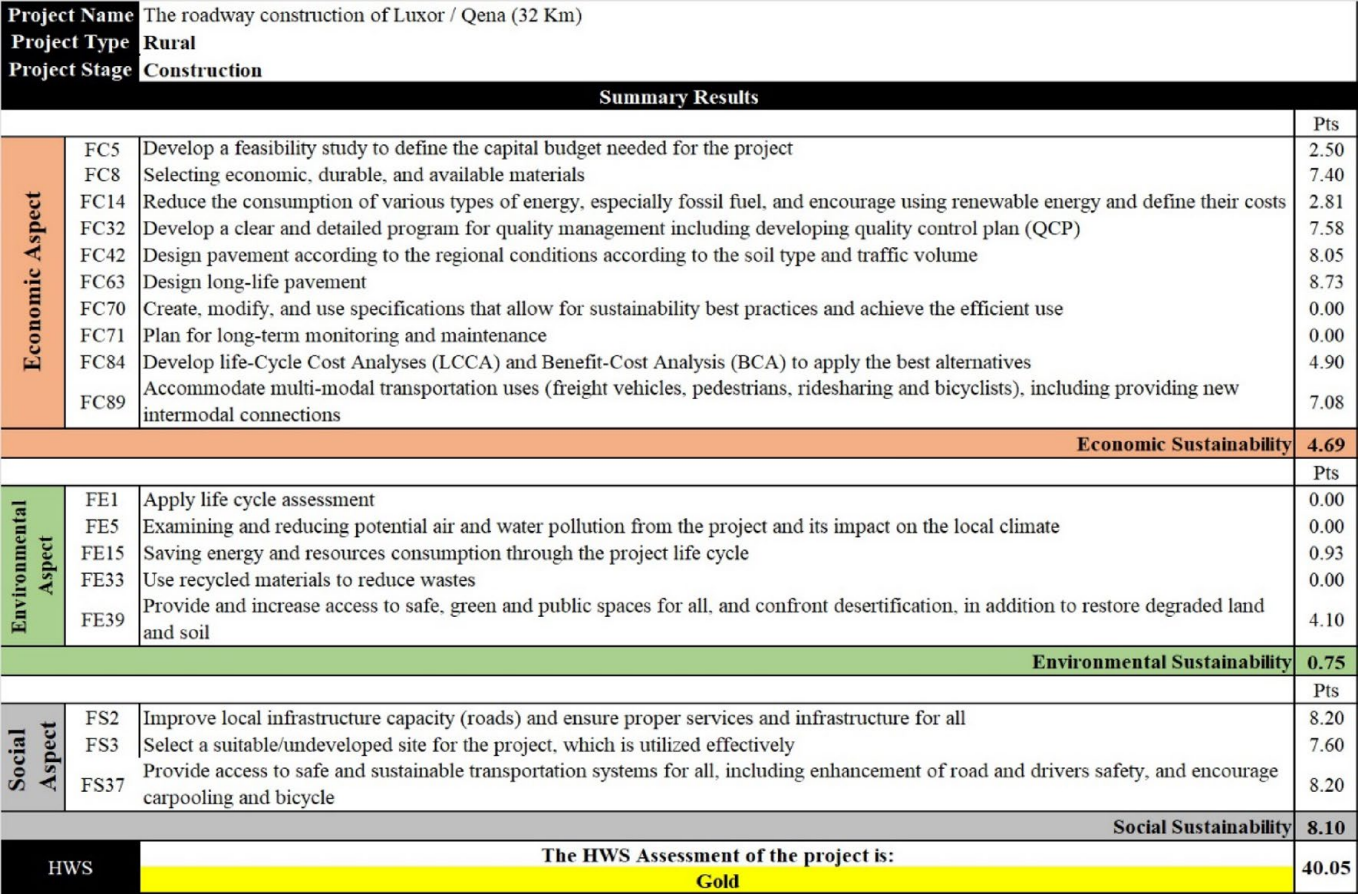
The sustainability performance for case study (2) through the HWS RS.

The second case study is for the construction of another carriageway for the existing 32 km rural highway connecting Luxor with Qena governorates in Upper Egypt (Table S18 in the supplementary). The aim of constructing a dual carriageway road for this project is to accommodate the traffic capacity and reduce the probability of accidents, providing safety and comfort for users. Figure 14 shows that the project’s sustainability performance level is 40.05% (Gold). Moreover, the case study was verified using the Envision RS, and the project achieved 280 out of 672 points (41.67%, Gold sustainability level) (Table S19 in the supplementary). This convergence with the Envision RS, used for evaluating HWS in local vision, shows that the HWS RS is valid for application in other developing countries, particularly those having social and economic conditions similar to Egypt. Furthermore, although the existence of only nine common factors in Envision RS and HWS RS (Fig. 9), the comparison results empower the feasibility and effectiveness of the developed RS, considering the most significant and representative factors.

By comparing the two case studies, it is found that the environmental score of case study (1) is higher than that of case study (2), while the economic and social scores of case study (2) are higher than those of case study (1). This is a result of using the FDR technique and Cold Mix Asphalt (CMA) in case study (1), which reduces the required energy and raw materials. On the other hand, the contractor could overcome the continuously arising of material prices in case study (2), in addition to providing a team for quality control. Furthermore, the project included a qualified system for the runoff discharge, and the dual carriageway road can reduce the accidents’ probability and convoy the population growth rate. Moreover, the project included stop amenities to provide comfort and safety for users.

As demonstrated, the developed RS with only 18 factors can evaluate highway projects as effectively as other international RSs. The selected factors encompass the most significant practices throughout the highway project life cycle. Furthermore, the small number of factors in the developed RS simplifies the evaluation process compared to the Envision RS with 59 factors, making it easier to use. It is also noteworthy that assessing a highway project with Envision takes 4 times longer than the assessment using the developed RS.

### HWS RS feedback

Before the professional usage of the developed RS, the model questions and their suggested scores were reviewed by a group of experts who were independent of those who participated in the initial surveys to ensure their relevance and credibility in identifying relevant factors. The model was reviewed by 7 highway experts and environmental consultants to evaluate its interface, ease of use, feasibility, and overall performance. Additionally, gathering feedback is a crucial step in obtaining recommendations for enhancing the model’s performance.

Depending on the experts’ evaluation, the model was modified to determine the overall expected sustainability performance for highway projects accurately and easily. One of the key outcomes of the feedback is the creation of a summary report, which provides guidelines for the factors that need consideration or improvement (Table S20 in the supplementary). These factors are ranked in ascendingly order based on their scores to indicate the enhancement priorities. Moreover, the FDR technique is considered one of the most crucial factors affecting economic sustainability, which is widely used in many highway projects. The feedback also led to modifying some questions to make them clearer or providing alternatives to simplify the evaluation process. Experts further recommended developing a certificate for the evaluated highway project, detailing aspect scores and overall sustainability performance.

## Implications

To the best of the authors’ knowledge, this is the first study providing an RS to evaluate HWS in Egypt. The factors were extracted from 150 highly related publications, SDGs, and 13 infrastructure RSs. The development of the RS required significant effort in carefully selecting and communicating with highly experienced engineers in highway projects to ensure the accuracy of the results obtained. These experts are familiar with the aspects related to highway projects and their related issues in Egypt. Most previous initiatives developed to evaluate HWS have limited the integration of all aspects of sustainability, all highway project stages, and the SDGs. Furthermore, the case studies applied to the developed RS and their comparison with Envision RS enriched the model’s validity and credibility. Lastly, this study underscores the importance of using AHP analysis for evaluating and ranking HWS factors, and it provides guidance for researchers on using statistical tools, such as RII, PCA, and the Pareto principle, to summarize data and identify the most significant factors affecting the study scope.

Although the examined literature has undertaken initiatives to evaluate HWS, they fall short compared to the developed study. For instance, the studies conducted by Montgomery and Bryce focused solely on environmental aspects, ignoring economic and social ones^23,30^, thus failing to represent the triple-bottom line of sustainability. Additionally, Zeiada’s study did not justify the criteria for factor selection and overlooked the SDGs^25^. Also, thirty factors were weighted using the AHP survey only, unlike the proposed study, which employed various statistical analyses to identify the factors of paramount influence on HWS. Moreover, the studies developed by Tsai and Inti focused on the design stage only, in addition to ignoring SDGs^21,28^.

Moreover, developing such an RS empowers developing countries, such as Egypt, to strengthen their plan towards achieving SDGs and increase their opportunities to enhance their annual overall performance among the 193 UN member states. The developed RS has effectively considered the SDGs by integrating 8 factors into the evaluation of HWS, accounting for 45% of the resulting evaluation factors (i.e., FS2, FS3, FS37, FE5, FE33, FE39, FC14, and FC70). On the other hand, the Envision RS, which has the second largest number of factors included among the extracted factors^31^, incorporates a smaller number of SDGs, as shown in Table 1. Compared to other infrastructure RSs, the developed model provides a more comprehensive consideration of the SDGs. This is achieved by analyzing all relevant targets and indicators within the study’s scope and integrating more SDGs targets than other infrastructure RSs, as illustrated in Table 6. As a result, the developed assessment model enables decision-makers to evaluate the sustainability performance of highway projects effectively, aligning with the practices of developed countries that have their own RSs. In addition, implementing highway projects that were certified by the developed RS prioritizes their contractors to be awarded for such projects according to the technical evaluation of bidding.

## Conclusions and recommendations

With the growing interest in sustainable development, it has become imperative to develop a comprehensive system for the sustainable evaluation of highway projects. Although several international RSs for highway infrastructure projects have been developed and implemented worldwide, no such RS has been tailored for highway projects in Egypt. This study aimed to develop an HWS RS that considers Egypt’s unique local conditions to evaluate the sustainability performance of highway projects throughout their life cycle, aligning with the SDGs and the triple-bottom line of sustainability.

To achieve this objective, 78 significant factors were identified from 150 highly relevant studies, the SDGs, and 13 existing RSs. These factors were analyzed and ranked using a questionnaire survey distributed to 100 experts in highway and construction management. Statistical analyses were performed to reduce the data into a smaller set of high-impact factors. This process resulted in the identification of 18 key factors (3 social, 5 environmental, and 10 economic) deemed critical to sustainable highway practices.

The AHP was applied to rank and weigh these selected factors through pairwise comparisons. These weights were then utilized to establish the proposed RS by formulating a set of weighted questions for each factor to quantify the sustainability performance of the analyzed projects.

The outcomes of this research aim to promote sustainable practices in highway construction. The developed RS offers practical guidelines that are easy to apply for designers and contractors, encouraging widespread adoption. Unlike other RSs, this model includes a summary report that identifies project deficiencies in achieving the target sustainability score for each aspect. It also provides specific recommendations for road designers and decision-makers to enhance sustainability evaluations based on their priorities and constraints.

This RS model addresses the limitations of other infrastructure RSs by providing a balanced approach to sustainability across the triple-bottom line throughout a project’s life cycle while considering the SDGs. However, some limitations were identified. The small sample size of respondents, although insightful, may limit the generalizability of the findings. Additionally, the absence of a local index integrating the three pillars of sustainability for highway projects restricted the ability to verify the case study results against other established RSs, hindering the depth of comparison with the proposed system. It is recommended that the developed RS be tested on a variety of local highway projects before being adopted for professional use. The factors and their relative weights should be continuously evaluated and updated to reflect changes in community priorities. Mandatory requirements should also be defined for evaluating HWS. Lastly, a cost-based optimization model could be developed to enhance overall project scores by improving the performance of factors with the lowest score.

## Supplementary Information

Below is the link to the electronic supplementary material.

Supplementary Material 1

## Data Availability

The datasets generated and/or analysed during the current study are not publicly available due to their large size and complexity. A representative sample is provided in the Supplementary Information, and the full dataset is available from the corresponding author on reasonable request.

## Abbreviations

AHP: Analytic Hierarchy Process
GHGs: Greenhouse gas emissions
CO_2_: Carbon dioxide
HWS: Highway Sustainability
UN: United Nations
LCCA: Life Cycle Cost Analysis
LEED: Leadership in Energy and Environmental Design
PCA: Principal Component Analysis
PVs: Priority vectors
RII: Relative Importance Index
RS: Rating System
SDGs: Sustainable Development Goals
FDR: Full Depth Recycling
CMA: Cold Mix Asphalt
