# Supplementary material for: An intertwined triple-bottom-line rating system for highway sustainability in developing countries
Source: Sci Rep. 2026 Feb 8;16:5433. doi: 10.1038/s41598-026-35183-4 (PMC12894824; doi:10.1038/s41598-026-35183-4)
Supplement: Supplementary file 1 — Supplementary Material 1 [file 41598_2026_35183_MOESM1_ESM.docx]

An Intertwined Triple-Bottom-Line Rating System for Highway Sustainability in Developing Countries

Mohamed O. Rageh ^a^^[[1]](#footnote-1)^, Emad E. Elbeltagi ^b^, Alaa R. Gabr ^c^, Sherif M. El-Badawy ^c^, Ibrahim A. Motawa ^a^

*^a^ Structural Engineering Department, Mansoura University, Egypt, (*[*eng.m_osami@mans.edu.eg*](mailto:eng.m_osami@mans.edu.eg)*, ID:* [*https://orcid.org/0000-0003-3205-1147*](https://orcid.org/0000-0003-3205-1147)*; ibrahim_a_motawa@mans.edu.eg, ID:* [*https://orcid.org/0000-0001-9664-1977*](https://orcid.org/0000-0001-9664-1977)*)
^b^ Department of Civil Engineering, College of Engineering, Qassim University, Saudi Arabia,* [*e.elbeltagi@qu.edu.sa*](mailto:e.elbeltagi@qu.edu.sa)*, ID:* *https://orcid.org/0000-0002-6568-3522*

*^c^ Public Works Engineering Department, Mansoura University, Egypt, (*[*eng-alaa1400@mans.edu.eg*](mailto:eng-alaa1400@mans.edu.eg)*, ID:* [*https://orcid.org/0000-0002-2399-2271*](https://orcid.org/0000-0002-2399-2271)*;* [*sbadawy@mans.edu.eg*](mailto:sbadawy@mans.edu.eg), *ID:* [*https://orcid.org/0000-0001-8348-1580*](https://orcid.org/0000-0001-8348-1580)*)*

**Supporting Information**

**Text S1** Detailed discussion of factors’ extraction

- In our preliminary work prior to this study, the factors were collected in a preliminary research study developed [1], which depends on analyzing previous studies, SDGs, and Infrastructure Rating systems RSs.
- The publications list used in that analysis was extracted from Scopus database, as it is one of the widely accessible sources of bibliographic data.
- The selection criterion was based on the main keywords related to the study scope. The search also included the title, keywords, and abstract of each article.
- As a result, 6,422 documents were obtained. The article titles and occasionally abstracts were manually scanned to exclude any articles that are not related to highway HWS.
- A content analysis was developed to reduce all these articles to the most relevant articles using direct citation, bibliographic coupling, and co-citation, which resulted in 150 highly-related articles.
- The 150 analyzed articles were carefully inspected to extract factors.
- SDGs with their 169 targets were analyzed to extract the relevant factors within the research scope.
- A total of 13 international rating systems and guidelines were analyzed to extract the key factors affecting the HWS.
- Three reference matrices were constructed to represent the identified factors as rows and the selected sources as columns.
- Consequently, each matrix column represents a source; either an article, SDGs, or RS, while each row is considered as an identified factor. If a related factor exists in a source, its corresponding cell has a value of 1.0; otherwise, 0.
- Based on factors’ mentions and co-occurrences with other factors, the importance of the identified factors was determined. Therefore, Degree Centrality (DC) was adopted, as it displays how many connections one factor has in comparison to the other network factors.
- After developing the reference matrices, Social Network Visualizer (SocnetV) software was used to analyze and visualize the networks of matrices. An adjacency matrix was developed to calculate the DC for the different nodes of each network (social, economic, and environmental networks) using the following equation, and the shown figure discusses how to obtain an adjacency matrix:

$$A_{nxn}=\left\{ \begin{aligned} W_{nxm} x W_{nxm}^{T} for i\neq j \\ 0 for i=j \end{aligned} \right.$$

Where *A_n×n_* is the adjacency matrix, and *n* equals all the analyzed factors; *W_n×m_* is a reference matrix, and *m* is the number of analyzed sources; $W_{nxm}^{T}$ is the transpose matrix of the corresponding reference matrix, as *j* and *i* are the matrix indices of the columns and rows, respectively.

After developing the adjacency matrix, DC is computed for each factor using the following equation:

$${DC}_{i}= \sum_{j:j\neq j} y_{i,j}$$

Where *DC_i_* is the degree centrality of factor *i*, and y*_i;j_* is the value in row *i* and column *j* of the adjacency matrix.

Therefore, normalized DC was computed as a dimensionless number that is dependent upon the size of the network. The normalized DC (ranges between 0 and 1) of factor *i* for each aspect is calculated as presented in the following equation:

$${Normalized DC}_{i}= \frac{{DC}_{i}}{{Max DC}_{i}in each aspect}$$

- All factors were revised to eliminate duplication, in addition to merging the factors with similar meaning.
- Then, the average normalized DC (ANDC) was calculated in each matrix (i.e., social, economic, and environmental), as shown in the following equation:

$$ANDC for each matrix= \frac{\sum_{1}^{n} {Normalized DC}_{i} for factors in each matrix}{n}$$

Where *n* is the number of factors in each matrix. Afterwards, the most important factors in each matrix were selected if the normalized DC is equal to or higher than the average normalized DC.

- We found that 78 factors out of 224 are of paramount influence on HWS depending on their values of degree Centrality.

**Table S1** Sustainability rating systems

- This table discusses the analyzed infrastructure RSs and provides data about the rating systems’ objectives, categories, publishing sector/university, and the launch date of the analyzed version.

| Rating System | Objectives | Categories | Publisher | Launch date |
| --- | --- | --- | --- | --- |
| Environmental Impact Assessment  (EIA) [2] | Evaluate projects affecting the environment, as it is concerned with environmental issues considered throughout the life cycle of the project, starting from the initial concept through the detailed design, construction, and operation to the demolition and land reuse | Class A (Pavement, roads < 5km) - Class B (Highway Construction till 50km + Pavement, roads > 5km) - Highway environmental studies (till 100km) - Class C (Highway Construction > 100km) | Environmental Management Sector, Ministry of State for Environmental Affairs, Egypt | April 2001 |
| Building Environmentally and Economically Transportation-Infrastructure-Highways  (BE^2^ST-in-Highways) [3] | Evaluates highway projects through some mandatory social factors. | Social Requirements, GHG Emission - Energy Use - Waste Reduction (Including Ex situ Materials, Recycling In situ Materials) - Water Consumption - Social Carbon Cost Saving - Life Cycle Cost - Traffic Noise - Hazardous Waste | Recycled Materials Resource Center, University of Wisconsin-Madison, USA | 2010 |
| Illinois Livability and Sustainable Transportation  (I-LAST)  [4] | - Minimizes the material and energy consumption. - Enhances the historic, scenic, and aesthetic context of highways. - Encourages community involvement in the transportation planning process.   Encourages non‐motorized transportation means. | Planning - Design - Environment - Water Quality - Transportation - Lighting - Materials - Innovation - Construction | Illinois Department of Transportation (IDOT), Illinois, USA. | 2010 |
| Leadership In Transportation and Environmental Sustainability  [GreenLITES (Design) V2.1]  [5] | - Conserving energy and natural resources during design stage. - Supporting the existence of alternative fuel use. - Improving access to public sites, protecting historic resources. - Multi-modal transportation.   Enhancing scenic and aesthetic characteristics of the roadside. | Sustainable Sites - Water Quality - Materials and Resources - Energy and Atmosphere – Innovation/Unlisted | New York State Department of Transportation (NYSDOT), USA | April 2011 |
| The Australian Green Infrastructure Council (AGIC) Rating  (scheme AGIC V2.1)  [6] | - Climate change adaptation. - Resilient infrastructure against storms, droughts, increased temperatures, and sea level rise.   Develops transport networks that recover from natural catastrophes, safeguarding coastal areas from rising sea levels and storm surges. | Transport and built infrastructure - Energy generation and transmission | Australian Green Infrastructure Council, Australia. | October 2011 |
| Leadership In Transportation and Environmental Sustainability  [GreenLITES (Operations and Maintenance)]  [7] | - Conserving energy and natural resources during design stage. - Supporting the existence of alternative fuel use. - Improving access to public sites, protecting historic resources. - Supporting multi-modal transportation.   Enhancing scenic and aesthetic characteristics of the roadside. | Bridges - Pavement - Drainage - Signals & Lighting (Traffic and Safety) - Snow and Ice - Facilities and Rest Areas - Roadside Environmental and Signs - Innovation/Unlisted | New York State Department of Transportation (NYSDOT), USA | February 2012 |
| GREENPAVE V2.1  [8] | - Minimizing raw materials usage. - Maximizing the use of recycled materials.   Reducing energy consumption and GHG emissions. | Pavement Technologies - Materials and Resources - Energy and Atmosphere - Innovation and Design Process. | Materials Engineering and Research Office, Canada | November 2017 |
| Envision V3.0  [9] | - Assessing sustainability and resilience of infrastructure. - Provides guidance for planning, designing, and delivery of sustainable infrastructure. - Incentivizes higher performance.   Helps stakeholders to implement cost-effective, resource-efficient, and long-term investments. | Quality of Life - Leadership - Resource Allocation - Natural World - Climate Resilience | Collaboration between Harvard University and the Institute for Sustainable Infrastructure (ISI) | 2018 |
| Infrastructure Voluntary Evaluation Sustainability Tool  (Invest V1.3)  [10] | Evaluating infrastructure through: System Planning for States modules (SPS), System Planning for Regions modules (SPR), Project Development module (PD), and Operations and Maintenance modules (OM). | Planning for states - Planning for regions - Project development - Operations and maintenance - Innovative criterion | The Federal Highway Administration (FHWA), USA. | April 2018 |
| Leadership in Energy and Environmental Design  (LEED V4.0)  [11] | Evaluating buildings and infrastructure projects. | Smart Locations - Neighborhood Design - Green Infrastructure - Innovation - Regional Priority. | [U.S. Green Building Council](https://www.usgbc.org/) (USGBC), USA | July 2018 |
| Sustainability Tracking, Assessment & Rating System​TM  (STARS V2.2)  [12] | Helps stakeholders to track and measure the environmental impacts in terms of resource consumption and pollution assessment. | Institutional Characteristics - Academics - Engagement - Operations - Planning and Administration - Innovation and Leadership | Association for Advancement of Sustainability in Higher Education, USA | June 2019 |
| Greenroads V2  [13] | - Cost reduction in the initial and maintenance stages. - Promoting health and safety - Engaging project participants. - Creating decent jobs. - Enhance durability and resilience.   Decreasing environmental issues. | Project Requirements - Environment & Water - Construction Activities - Materials & Design - Utilities & Controls - Access & Livability - Creativity & Effort | Washington University | February 2020 |
| Leadership in Energy and Environmental Design  (LEED V4.1)  [14] | Evaluating projects in terms of energy consumption and materials used | Integrative Process - Transportation - Sustainable Sites - Water and Energy Efficiency - Materials and Resources - Indoor Environmental Quality - Innovation - Regional Priority. | [U.S. Green Building Council](https://www.usgbc.org/) (USGBC), USA | October 2021 |

**Table S2** Factors of highway sustainability collected from literature review, SDGs, and RSs **(Rageh et al., 2023)**

- This table shows the 224 factors extracted from previous studies, SDGs, and analyzed RSs, which developed in the preliminary study [1]. The factors are classified through the triple-bottom line of sustainability.

| **Aspect** | **ID** | **Factors** |
| --- | --- | --- |
| Social | FS1 | Meeting the balance between community needs and transportation needs |
|  | FS2 | Improving the local infrastructure capacity (roads) and ensuring proper services and infrastructure for all |
|  | FS3 | Selecting a suitable/undeveloped site for the project, which is utilized effectively |
|  | FS4 | Protecting the natural, historical, and cultural heritage |
|  | FS5 | Creating decent job opportunities and activating the local business sector |
|  | FS6 | Direct and indirect employment and reducing the unemployment rate |
|  | FS7 | Provision of roads for rural and urban areas |
|  | FS8 | Developing risk assessment to identify and reduce any disasters or future risks to the public and road users |
|  | FS9 | Land provision after the demolition stage of the project |
|  | FS10 | Safety of labors and the public during project demolition and maintenance |
|  | FS11 | Promotion of public awareness of the demolition stage |
|  | FS12 | Ability to use the road at any time, reduce operational delays and increase safety |
|  | FS13 | Protecting community health and safety during the operation stage |
|  | FS14 | Diversity of employees and community by attracting and retaining workforce |
|  | FS15 | Achieving flexibility in work times during construction and operation stages |
|  | FS16 | Increasing social sustainability awareness using educational programs |
|  | FS17 | Providing training for workers |
|  | FS18 | Protecting labor rights |
|  | FS19 | Reducing disturbance and vibrations to surrounding neighborhoods and environments through construction and operation stages |
|  | FS20 | Upgrading local skills and capabilities through training programs |
|  | FS21 | Strengthening flexibility and adaptivity to climate-related hazards and disasters |
|  | FS22 | Methodologies for effective planning and management of climate change issues |
|  | FS23 | Reducing the global maternal death ratio due to traffic congestion |
|  | FS24 | Decreasing the number of global fatalities and injuries from road accidents |
|  | FS25 | Reducing the number of fatalities and people affected by the economic losses |
|  | FS26 | End hunger and ensure access by all people to safe and sufficient food all year |
|  | FS27 | Sustainable food production and preserving the productive farmland |
|  | FS28 | Achieving a minimum proficiency level in reading and mathematics |
|  | FS29 | Having access to qualified-early childhood development |
|  | FS30 | Achieving qualified technical and vocational, including university education |
|  | FS31 | Technology updating to supply renewable energy services and projects |
|  | FS32 | Elimination of forced and child labor, modern slavery, and human trafficking |
|  | FS33 | Implementing and accepting a global youth employment plan |
|  | FS34 | Designing highways according to traffic flows for all transportation modes and freights to reduce congestion, and enhancing freight features |
|  | FS35 | Achieving safe, regular, and responsible migration and mobility of people |
|  | FS36 | Provision of safe and affordable housing and basic services |
|  | FS37 | Provision of safe, affordable, sustainable, public and private transportation systems for all, including enhancement of road and drivers safety, and encourage carpooling and bicycle |
|  | FS38 | Participation of communities in urban planning and decision making |
|  | FS39 | Adopting sustainable practices and integrating sustainability information in reports |
|  | FS40 | Encouraging sustainable procurement through transparent and fair contracts |
|  | FS41 | Implementing programs’ framework of sustainable consumption and production |
|  | FS42 | Reducing all types of corruption and bribery |
|  | FS43 | Measurement of the population's satisfaction with public services |
|  | FS44 | Sharing knowledge, expertise, and financial resources between the project team and stakeholders |
|  | FS45 | Preserving the physical and visual character of the project site and its landscapes |
|  | FS46 | Stakeholder participation of early involvement in decision-making |
|  | FS47 | Provision of safe, comfortable, and convenient facilities for all |
|  | FS48 | Using pedestrian countdown for a safe crossing and for increasing the sidewalk use |
|  | FS49 | Scenic overlook and safety parking area enhancements |
|  | FS50 | Effective leadership and achieving project sustainability goals |
|  | FS51 | Creating a project sustainability management plan |
|  | FS52 | Intersections for special use lanes, and add bus turnouts and stops |
| Economic | FC1 | Determining the project profitability during the project operation |
|  | FC2 | The project serves the local economy and achieves higher levels of productivity for labor-intensive sectors |
|  | FC3 | Improving the living standard of the communities |
|  | FC4 | Determining the life cycle cost and providing financial and technical support |
|  | FC5 | Developing a feasibility study to define the capital budget needed |
|  | FC6 | Developing a project finance schedule to support investments |
|  | FC7 | Determining all payments of capital investments from all financial resources |
|  | FC8 | Selecting economic, durable, and available materials |
|  | FC9 | The interests for the capital cost paid for both a fixed loan and liquid capital |
|  | FC10 | Investments directed to each project that support community goals |
|  | FC11 | Provision of suitable salaries and fees for all staff during the project life cycle |
|  | FC12 | Determining costs for all types of materials during the project stages |
|  | FC13 | Indirect employment associated with project operation and maintenance |
|  | FC14 | Reducing the consumption of energy and fossil fuel, and encouraging renewable energy |
|  | FC15 | Reducing the consumption of potable surface and groundwater resources and defining their costs |
|  | FC16 | Determining costs for using and installing various tools, vehicles, and equipment |
|  | FC17 | Determining costs for purchasing and installing various equipment, such as plants |
|  | FC18 | Developing various types of protecting the public and labors during construction |
|  | FC19 | Creating a balance sheet and periodically check it with the project control team |
|  | FC20 | Determining the materials procurement and stock costs, including transportation |
|  | FC21 | Type and consumption of energy used for crushing, transporting, and relocating |
|  | FC22 | Costs for waste loading and unloading, transportation, and disposals charges |
|  | FC23 | Compensating value paid to affected parties during the demolition process |
|  | FC24 | Provision of pensions and unemployment compensation |
|  | FC25 | Compensation made for the damaged environment |
|  | FC26 | The land value after demolition for re-development |
|  | FC27 | Defining the valuable residues for reuse and recycling, such as steel, brick, and stone |
|  | FC28 | Reducing vehicle operating cost (VOC) |
|  | FC29 | Reducing travel time and distance traveled |
|  | FC30 | Determining accident costs |
|  | FC31 | Clear duties and obligations for the project parties |
|  | FC32 | Developing a detailed program for quality management including developing a quality control plan (QCP) |
|  | FC33 | Using pavement monitoring and management programs |
|  | FC34 | Efficiency of the owner’s inspection team |
|  | FC35 | Technical assessment of the contractor during the bidding process |
|  | FC36 | Selection of the lowest bidder to construct the project |
|  | FC37 | Contractor’s experience assessment |
|  | FC38 | Performing continuous evaluation of the contractor’s financial status during construction |
|  | FC39 | Determining the amount of sub-contracted work |
|  | FC40 | Availability of financial incentives to the contractor to produce higher quality |
|  | FC41 | Reducing delays in contactor progress payment |
|  | FC42 | Pavement design is executed according to the regional conditions |
|  | FC43 | Design errors arising from inadequate assumptions and inaccurate data |
|  | FC44 | Insufficient owner involvement throughout the design stage |
|  | FC45 | Accuracy of investigation performed on existed soil type |
|  | FC46 | Accuracy of traffic volume and population data, and connecting the traffic and crash data with medical databases |
|  | FC47 | Using construction materials suitable for the climate |
|  | FC48 | Specifications accuracy of the required foundation works due to soil conditions |
|  | FC49 | Limitations on materials sources, equipment type, and construction method |
|  | FC50 | Defining the asphalt mix properties (e.g., stability, durability, and workability) |
|  | FC51 | Availability of experienced staff in the owner’s and contractor’s teams |
|  | FC52 | Variation in aggregates gradation in mixing, transportation, and placement |
|  | FC53 | Determining the quantities of filling materials in the mixture |
|  | FC54 | Paver and roller mechanical condition and type |
|  | FC55 | Determining the recycling cost |
|  | FC56 | Determining general expenses and taxes |
|  | FC57 | Using by-product materials and demolition wastes |
|  | FC58 | Using local materials |
|  | FC59 | Prevention of asphalt segregation |
|  | FC60 | Lives’ extension of seal coats to reduce the impact of frequent rehabilitation |
|  | FC61 | Developing an optimum design for longitudinal joints |
|  | FC62 | Pavement performance tracking |
|  | FC63 | Durable pavement design |
|  | FC64 | Using modern techniques for the pavement design |
|  | FC65 | Reducing the quantity of materials used for rehabilitation |
|  | FC66 | Reducing lighting costs by using renewable energy sources, replacing signs with reflective signs, and converting light bulbs to LED to save electricity |
|  | FC67 | Improving efficiency and quality of construction and selected materials |
|  | FC68 | Construction acceleration to reduce congestion without additional resource consumption |
|  | FC69 | Determining the contractor warranty |
|  | FC70 | Modifying and using specifications that allow for sustainability best practices |
|  | FC71 | Long-term monitoring and maintenance plan |
|  | FC72 | Designing for emergencies (e.g., earthquakes, floods, and eco-environmental accidents) |
|  | FC73 | Improving global resource efficiency in consumption and production |
|  | FC74 | Strengthening the scientific and technological capacity to achieve sustainability |
|  | FC75 | Increasing financial resources to conserve biodiversity and ecosystems |
|  | FC76 | Increasing the exports by doubling their share of global exports |
|  | FC77 | Determining financial flows of renewable energy production and research |
|  | FC78 | Promoting the development and diffusion of environmental technologies |
|  | FC79 | Avoiding surface construction on flood-prone zones by relocating constructions in higher elevations or implementing localized flood walls |
|  | FC80 | Existing alternatives for curing concrete instead of water |
|  | FC81 | Dewatering reuse |
|  | FC82 | Greywater/industrial or wastewater reuse |
|  | FC83 | Locating projects on sites classified as brownfields which are remediated |
|  | FC84 | Developing LCCA and BCA to filter the project alternatives |
|  | FC85 | Using alternatives to dust suppression instead of water, such as dry agents |
|  | FC86 | Stormwater harvesting |
|  | FC87 | Reuse of topsoil |
|  | FC88 | Reuse of previous pavement as sub-base |
|  | FC89 | Accommodating multi-modal transportation uses (freight vehicles, pedestrians, ridesharing, and bicyclists) including providing intermodal connections |
|  | FC90 | Soil stabilization with cementitious and recycled materials |
|  | FC91 | Saving transportation facilities for older metropolitan and heritage areas |
|  | FC92 | Using transportation data within the transportation planning process |
|  | FC93 | Implementing and monitoring an effective road weather management program and Work Zone Traffic Control (WZTC) |
|  | FC94 | Provision of handling and storage areas for reused/recycled construction materials |
|  | FC95 | Cement/Asphalt production using energy and fuel-saving technologies |
|  | FC96 | In‐place asphalt pavement recycling |
|  | FC97 | Bridges preservation and retrofitting |
|  | FC98 | Utilizing technologies (e.g., V2V and V2I) to improve the system efficiency |
| Environmental | FE1 | Applying life cycle assessment (LCA) |
|  | FE2 | Avoiding the negative impacts on the environment by paying attention to air quality and waste management, and reducing fine particles |
|  | FE3 | Defining potential ecological risks and developing risk management strategies |
|  | FE4 | Tracking environmental effects |
|  | FE5 | Examining and reducing air and water pollution and its impact on local climate |
|  | FE6 | Examining and reducing nuisance during the construction and operation stages |
|  | FE7 | Examining and reducing waste generation at the construction and operation stages |
|  | FE8 | Understanding the environmental and energy-saving problems of the project |
|  | FE9 | Integrating all environmental considerations into flexible design through the LC |
|  | FE10 | Using modular and standardized components to reduce wastes |
|  | FE11 | Avoiding, reducing, and compensating the loss of natural habitat of flora and fauna |
|  | FE12 | Avoiding the generation of GHG emissions resulted from the project life cycle |
|  | FE13 | Constructing barriers and providing plants as a buffer zone to reduce nuisance |
|  | FE14 | Release of chemical waste and organic pollutants through dumping and landfills |
|  | FE15 | Saving energy and resources consumption through the project life cycle |
|  | FE16 | Existing of on-site health and safety by reducing the number of accidents, providing on-site supervision, and providing training programs to employees |
|  | FE17 | Reducing the release of emissions to protect the ozone layer |
|  | FE18 | Reducing emissions from construction equipment/vehicles by using emission reduction exhaust retrofit and alternative fuels |
|  | FE19 | Reducing on-site waste by using an off-site fabrication |
|  | FE20 | Recycling and reusing of useful materials, such as rubber, asphalt, and concrete |
|  | FE21 | Minimizing the earthwork of soil and other excavated/filling materials off-site |
|  | FE22 | Controlling environmental impacts from drainage, landscaping, and waste curing |
|  | FE23 | Provision of an environmental management team and supervision |
|  | FE24 | Existing of environmental management system |
|  | FE25 | Adoption of clean and environmentally technologies to obtain sustainable facility |
|  | FE26 | Applying environmental protection laws and regulations on construction activities |
|  | FE27 | Using non-potable water for hygiene and irrigation to reduce potable water use |
|  | FE28 | Developing a demolition plan for hazardous materials and waste reduction, and giving special treatment to toxic materials, heavy metals, and chemicals |
|  | FE29 | Provision of control and supervision for the demolition activities |
|  | FE30 | Adoption of technologies to reduce the disturbance on eco-environment systems |
|  | FE31 | Classification of wastes to enable effective treatment and disposal |
|  | FE32 | Using permeable materials |
|  | FE33 | Using recycled materials to reduce wastes |
|  | FE34 | Using recyclable materials |
|  | FE35 | Using noise reduction materials, or use quieter pavement surface types |
|  | FE36 | Using fiber materials |
|  | FE37 | Minimizing aggregate transportation to reduce congestion and emissions |
|  | FE38 | Using sustainable and qualified materials |
|  | FE39 | Providing access to safe, green, and public spaces for all, and confronting desertification, in addition to restoring degraded land and soil |
|  | FE40 | Specifications accuracy of aggregates quality and gradation |
|  | FE41 | Specifications accuracy of asphalt quality and type |
|  | FE42 | Selecting suitable materials for hot climate regions and heavy-loading traffic |
|  | FE43 | Using renewable resources/energy and reducing the use of petroleum materials |
|  | FE44 | Protecting the site soil from pollution |
|  | FE45 | Utilizing cool pavement surface types to reduce the urban heat island effect |
|  | FE46 | Utilizing Warm Mix Asphalt (WMA) instead of Hot Mix Asphalt (HMA) |
|  | FE47 | Specifying asphalt pavement mixtures containing recycled asphalt pavement (RAP) |
|  | FE48 | Specifying PCC pavement mixtures containing recycled concrete aggregate (RCA) |
|  | FE49 | Increasing pavement albedo |
|  | FE50 | Reducing virgin binder content in asphalt concrete |
|  | FE51 | Reducing the impact of aggregate extracting, manufacturing, and transportation |
|  | FE52 | Provision of documented analysis (e.g., LCA or EIA) proving that the project design reduces carbon footprint by developing |
|  | FE53 | Increasing the environmental awareness for the public through training programs |
|  | FE54 | Provision of environmentally-friendly maintenance alternatives |
|  | FE55 | Increasing pavement performance and smoothness |
|  | FE56 | Reducing light pollution resulted from excessive lighting of lampposts, advertisement boards, and transportation means |
|  | FE57 | Developing a periodic assessment of highway operation and maintenance in terms of air and water quality, emissions, and noise impacts |
|  | FE58 | Reducing the number of deaths and illnesses from hazardous pollution |
|  | FE59 | Conserving coastal and marine areas, and protecting marine from pollution |
|  | FE60 | Developing effective flood warning protocol and improving stormwater quality of runoff by treating rainwater as a resource rather than as a waste |
|  | FE61 | Specifying and monitoring all energy systems |
|  | FE62 | Including an erosion and sedimentation control plan for construction activities and using recycled materials for the maintenance and repair of banks and steep slopes |
|  | FE63 | Reducing the use of inorganic fertilizers and chemical pesticides |
|  | FE64 | Promoting sustainable site vegetation by choosing native and non‐invasive species |
|  | FE65 | Using low/zero-emission vehicles (Electric vehicles), walking and cycling |
|  | FE66 | Improved shading through vegetation at park-and-ride lots |
|  | FE67 | Cleaning bridges and disposing of sweepings to avoid drainage blockage |
|  | FE68 | Minimizing and recycling garage wastes by developing a waste management plan |
|  | FE69 | Increasing the density of asphalt concrete pavement |
|  | FE70 | Promoting LEED expertise throughout design, construction, and operation |
|  | FE71 | Selecting products from manufacturers who have verified improved environmental life-cycle impacts |
|  | FE72 | Delivering products verified to have improved life-cycle impacts |
|  | FE73 | Prevention of chemicals and liquids spill from equipment and vehicles |
|  | FE74 | Reducing emissions resulted from vehicles by using emission reduction exhaust retrofit and alternative fuels |

**Table S3** The most important HWS factors **(Rageh et al., 2023)**

- This table shows the most important factors as a result of the Social Network Analysis developed in the preliminary study [1].

| Aspect | ID | Factors |
| --- | --- | --- |
| Social | FS1 | Meeting the balance between community needs and transportation needs |
|  | FS2 | Improving the local infrastructure capacity (roads) and ensuring proper services and infrastructure for all |
|  | FS3 | Selecting a suitable/undeveloped site for the project, which is utilized effectively |
|  | FS4 | Protecting the natural, historical, and cultural heritage |
|  | FS6 | Direct and indirect employment and reducing the unemployment rate |
|  | FS7 | Provision of roads for rural and urban areas |
|  | FS8 | Developing risk assessment to identify and reduce any disasters or future risks to the public and road users |
|  | FS10 | Safety of labors and the public during project demolition and maintenance |
|  | FS13 | Protecting community health and safety during the operation stage |
|  | FS19 | Reducing disturbance and vibrations to surrounding neighborhoods and environments through construction and operation stages |
|  | FS20 | Upgrading local skills and capabilities through training programs |
|  | FS34 | Designing highways according to traffic flows for all transportation modes and freights to reduce congestion, and enhancing freight features |
|  | FS37 | Provision of safe, affordable, sustainable, public and private transportation systems for all, including enhancement of road and drivers safety, and encourage carpooling and bicycle |
|  | FS38 | Participation of communities in urban planning and decision making |
|  | FS44 | Sharing knowledge, expertise, and financial resources between the project team and stakeholders |
|  | FS45 | Preserving the physical and visual character of the project site and its landscapes |
|  | FS47 | Provision of safe, comfortable, and convenient facilities for all |
| Economic | FC1 | Determining the project profitability during the project operation |
|  | FC2 | The project serves the local economy and achieves higher levels of productivity for labor-intensive sectors |
|  | FC3 | Improving the living standard of the communities |
|  | FC4 | Determining the life cycle cost and providing financial and technical support |
|  | FC5 | Developing a feasibility study to define the capital budget needed |
|  | FC6 | Developing a project finance schedule to support investments |
|  | FC7 | Determining all payments of capital investments from all financial resources |
|  | FC8 | Selecting economic, durable, and available materials |
|  | FC10 | Investments directed to each project that support community goals |
|  | FC11 | Provision of suitable salaries and fees for all staff during the project life cycle |
|  | FC14 | Reducing the consumption of energy and fossil fuel, and encouraging renewable energy |
|  | FC15 | Reducing the consumption of potable surface and groundwater resources and defining their costs |
|  | FC16 | Determining costs for using and installing various tools, vehicles, and equipment |
|  | FC18 | Developing various types of protecting the public and labors during construction |
|  | FC32 | Developing a detailed program for quality management including developing a quality control plan (QCP) |
|  | FC37 | Contractor’s experience assessment |
|  | FC38 | Performing continuous evaluation of the contractor’s financial status during construction |
|  | FC42 | Pavement design is executed according to the regional conditions |
|  | FC56 | Determining general expenses and taxes |
|  | FC57 | Using by-product materials and demolition wastes |
|  | FC58 | Using local materials |
|  | FC63 | Durable pavement design |
|  | FC64 | Using modern techniques for the pavement design |
|  | FC66 | Reducing lighting costs by using renewable energy sources, replacing signs with reflective signs, and converting light bulbs to LED to save electricity |
|  | FC67 | Improving efficiency and quality of construction and selected materials |
|  | FC68 | Construction acceleration to reduce congestion without additional resource consumption |
|  | FC70 | Modifying and using specifications that allow for sustainability best practices |
|  | FC71 | Long-term monitoring and maintenance plan |
|  | FC78 | Promoting the development and diffusion of environmental technologies |
|  | FC84 | Developing LCCA and BCA to filter the project alternatives |
|  | FC89 | Accommodating multi-modal transportation uses (freight vehicles, pedestrians, ridesharing, and bicyclists) including providing intermodal connections |
|  | FC91 | Saving transportation facilities for older metropolitan and heritage areas |
| Environmental | FE1 | Applying life cycle assessment (LCA) |
|  | FE2 | Avoiding the negative impacts on the environment by paying attention to air quality and waste management, and reducing fine particles |
|  | FE5 | Examining and reducing air and water pollution and its impact on local climate |
|  | FE7 | Examining and reducing waste generation at the construction and operation stages |
|  | FE11 | Avoiding, reducing, and compensating the loss of natural habitat of flora and fauna |
|  | FE12 | Avoiding the generation of GHG emissions resulted from the project life cycle |
|  | FE13 | Constructing barriers and providing plants as a buffer zone to reduce nuisance |
|  | FE14 | Release of chemical waste and organic pollutants through dumping and landfills |
|  | FE15 | Saving energy and resources consumption through the project life cycle |
|  | FE18 | Reducing emissions from construction equipment/vehicles by using emission reduction exhaust retrofit and alternative fuels |
|  | FE20 | Recycling and reusing of useful materials, such as rubber, asphalt, and concrete |
|  | FE21 | Minimizing the earthwork of soil and other excavated/filling materials off-site |
|  | FE27 | Using non-potable water for hygiene and irrigation to reduce potable water use |
|  | FE28 | Developing a demolition plan for hazardous materials and waste reduction, and giving special treatment to toxic materials, heavy metals, and chemicals |
|  | FE31 | Classification of wastes to enable effective treatment and disposal |
|  | FE33 | Using recycled materials to reduce wastes |
|  | FE37 | Minimizing aggregate transportation to reduce congestion and emissions |
|  | FE39 | Providing access to safe, green, and public spaces for all, and confronting desertification, in addition to restoring degraded land and soil |
|  | FE43 | Using renewable resources/energy and reducing the use of petroleum materials |
|  | FE44 | Protecting the site soil from pollution |
|  | FE47 | Specifying asphalt pavement mixtures containing recycled asphalt pavement (RAP) |
|  | FE48 | Specifying PCC pavement mixtures containing recycled concrete aggregate (RCA) |
|  | FE51 | Reducing the impact of aggregate extracting, manufacturing, and transportation |
|  | FE53 | Increasing the environmental awareness for the public through training programs |
|  | FE56 | Reducing light pollution resulted from excessive lighting of lampposts, advertisement boards, and transportation means |
|  | FE57 | Developing a periodic assessment of highway operation and maintenance in terms of air and water quality, emissions, and noise impacts |
|  | FE60 | Developing effective flood warning protocol and improving stormwater quality of runoff by treating rainwater as a resource rather than as a waste |
|  | FE62 | Including an erosion and sedimentation control plan for construction activities and using recycled materials for the maintenance and repair of banks and steep slopes |
|  | FE64 | Promoting sustainable site vegetation by choosing native and non‐invasive species |

**Table S4** Distribution of factors in the breakdown structure

- This table shows the clustering of the analyzed factors according to the categorization of the Envision RS.

| Category | Sub-category | Factors |
| --- | --- | --- |
| Quality of Life | Well-Being | FS3 - FS19 - FS47 |
|  |  | FC3 |
|  |  | FE56 |
|  | Mobility | FC89 - FC91 |
|  | Community | FS1 - FS2 - FS4 - FS7 - FS37 - FS38 - FS45 |
| Resource Allocation | Materials | FC8 - FC12 - FC57 - FC70 |
|  |  | FE20 - FE21 - FE28 - FE31 - FE47 - FE48 |
|  | Energy | FC14 - FC68 |
|  |  | FE15 - FE43 |
|  | Water | FC15 |
|  |  | FE14 - FE27 |
| Natural World | Flora and Fauna | FE11 - FE39 - FE64 |
|  | Conservation | FS3 |
|  |  | FE60 - FE62 |
|  | Ecology | FE44 |
| Climate | Resilience | FS8 |
|  |  | FC78 |
|  |  | FE1 - FE53 |
|  | Emissions | FC58 |
|  |  | FE2 - FE5 - FE7 - FE12 - FE13 - FE18 - FE33 - FE37 - FE51 - FE57 |
| Structure | Planning | FS34 |
|  |  | FC1 - FC4 - FC37 - FC38 - FC42 - FC63 - FC64 |
|  | Construction | FC18 - FC66 - FC67 |
|  | Operation and Maintenance | FS10 |
|  |  | FC71 |
| Leadership | Collaboration | FS44 |
|  | Planning | FC32 |
|  | Economy | FS6 - FS20 |
|  |  | FC2 - FC5 - FC6 - FC10 - FC11 - FC16 - FC56 - FC84 |

**Table S5** Sample of responses

- The table provides a sample of 13 responses out of 100, which rank the analyzed factors from 1 to 5 in terms of their importance.

| Factor | Res. 1 | Res. 2 | Res. 3 | Res. 4 | Res. 5 | Res. 6 | Res. 7 | Res. 8 | Res. 9 | Res. 10 | Res. 11 | Res. 12 | Res. 13 |
| --- | --- | --- | --- | --- | --- | --- | --- | --- | --- | --- | --- | --- | --- |
| FS13 | 4 | 4 | 4 | 4 | 5 | 5 | 5 | 5 | 4 | 5 | 3 | 5 | 3 |
| FS19 | 4 | 5 | 4 | 3 | 4 | 4 | 4 | 3 | 5 | 5 | 4 | 4 | 5 |
| FS47 | 4 | 5 | 4 | 3 | 4 | 5 | 4 | 4 | 4 | 4 | 5 | 4 | 4 |
| FC3 | 4 | 5 | 3 | 4 | 4 | 5 | 5 | 4 | 4 | 5 | 4 | 3 | 5 |
| FE56 | 2 | 5 | 3 | 4 | 4 | 4 | 2 | 5 | 4 | 4 | 4 | 4 | 3 |
| FC89 | 5 | 4 | 5 | 3 | 5 | 5 | 5 | 4 | 3 | 5 | 5 | 5 | 5 |
| FC91 | 5 | 5 | 4 | 4 | 4 | 3 | 5 | 3 | 5 | 5 | 3 | 2 | 2 |
| FS1 | 5 | 4 | 4 | 5 | 5 | 5 | 5 | 5 | 3 | 5 | 4 | 4 | 5 |
| FS2 | 5 | 5 | 5 | 4 | 4 | 5 | 5 | 5 | 5 | 5 | 4 | 5 | 5 |
| FS4 | 5 | 4 | 3 | 4 | 5 | 2 | 5 | 4 | 5 | 5 | 4 | 5 | 5 |
| FS7 | 5 | 4 | 5 | 4 | 5 | 5 | 5 | 3 | 5 | 5 | 5 | 5 | 5 |
| FS37 | 5 | 4 | 5 | 2 | 4 | 5 | 5 | 4 | 4 | 5 | 5 | 4 | 4 |
| FS38 | 4 | 4 | 4 | 4 | 4 | 5 | 5 | 3 | 4 | 3 | 2 | 3 | 4 |
| FS45 | 4 | 4 | 5 | 3 | 5 | 5 | 4 | 5 | 4 | 4 | 4 | 3 | 5 |
| FC8 | 5 | 4 | 4 | 5 | 5 | 4 | 4 | 4 | 3 | 5 | 5 | 5 | 5 |
| FC12 | 5 | 4 | 2 | 4 | 4 | 5 | 4 | 4 | 3 | 5 | 4 | 4 | 4 |
| FC57 | 2 | 5 | 3 | 3 | 4 | 5 | 5 | 5 | 3 | 5 | 4 | 4 | 5 |
| FC70 | 5 | 4 | 4 | 5 | 5 | 5 | 5 | 5 | 4 | 5 | 5 | 5 | 5 |
| FE20 | 4 | 5 | 2 | 3 | 4 | 4 | 5 | 5 | 4 | 5 | 5 | 4 | 4 |
| FE21 | 5 | 5 | 3 | 5 | 5 | 1 | 2 | 4 | 4 | 5 | 4 | 5 | 5 |
| FE28 | 5 | 4 | 5 | 5 | 5 | 5 | 4 | 4 | 4 | 5 | 4 | 4 | 5 |
| FE31 | 4 | 4 | 3 | 4 | 4 | 5 | 4 | 4 | 4 | 5 | 3 | 4 | 5 |
| FE47 | 3 | 4 | 4 | 4 | 4 | 5 | 4 | 5 | 4 | 2 | 2 | 5 | 4 |
| FE48 | 4 | 5 | 3 | 3 | 4 | 3 | 4 | 5 | 4 | 2 | 2 | 5 | 3 |
| FC14 | 4 | 4 | 4 | 4 | 4 | 5 | 5 | 3 | 5 | 5 | 3 | 3 | 5 |
| FC68 | 5 | 4 | 3 | 5 | 5 | 5 | 5 | 4 | 4 | 5 | 3 | 3 | 3 |
| FE15 | 5 | 4 | 3 | 5 | 5 | 5 | 5 | 4 | 5 | 5 | 3 | 3 | 5 |
| FE43 | 3 | 4 | 3 | 3 | 5 | 4 | 4 | 4 | 5 | 5 | 2 | 3 | 5 |
| FC15 | 4 | 5 | 2 | 5 | 4 | 1 | 4 | 3 | 5 | 5 | 3 | 3 | 5 |
| FE14 | 4 | 3 | 4 | 5 | 4 | 4 | 5 | 4 | 4 | 5 | 5 | 5 | 3 |
| FE27 | 5 | 4 | 4 | 5 | 5 | 5 | 5 | 2 | 5 | 5 | 5 | 4 | 5 |
| FE11 | 3 | 3 | 4 | 3 | 5 | 5 | 4 | 5 | 4 | 5 | 5 | 4 | 5 |
| FE39 | 3 | 4 | 4 | 4 | 5 | 5 | 4 | 5 | 4 | 5 | 4 | 3 | 5 |
| FE64 | 3 | 4 | 4 | 4 | 5 | 5 | 4 | 5 | 4 | 5 | 3 | 3 | 3 |
| FS3 | 5 | 4 | 4 | 5 | 4 | 4 | 4 | 4 | 4 | 5 | 4 | 5 | 5 |
| FE60 | 4 | 5 | 5 | 4 | 5 | 5 | 4 | 5 | 5 | 5 | 2 | 4 | 4 |
| FE62 | 3 | 4 | 4 | 4 | 5 | 4 | 3 | 5 | 5 | 5 | 4 | 5 | 4 |
| FE44 | 3 | 4 | 2 | 4 | 5 | 5 | 3 | 3 | 4 | 5 | 4 | 4 | 5 |
| FS8 | 4 | 4 | 4 | 5 | 5 | 5 | 4 | 5 | 5 | 5 | 4 | 4 | 5 |
| FC78 | 4 | 4 | 3 | 4 | 5 | 4 | 4 | 5 | 4 | 5 | 4 | 4 | 3 |
| FE1 | 5 | 4 | 4 | 5 | 5 | 5 | 4 | 5 | 4 | 4 | 4 | 4 | 5 |
| FE53 | 4 | 4 | 3 | 4 | 5 | 5 | 5 | 4 | 5 | 5 | 4 | 4 | 5 |
| FC58 | 5 | 4 | 4 | 4 | 3 | 4 | 5 | 5 | 3 | 3 | 5 | 3 | 5 |
| FE2 | 4 | 4 | 5 | 3 | 5 | 5 | 4 | 5 | 4 | 5 | 3 | 3 | 4 |
| FE5 | 4 | 4 | 4 | 3 | 5 | 5 | 4 | 4 | 5 | 5 | 3 | 4 | 3 |
| FE7 | 4 | 5 | 4 | 5 | 4 | 2 | 4 | 4 | 4 | 5 | 4 | 3 | 3 |
| FE12 | 2 | 5 | 4 | 4 | 4 | 5 | 3 | 4 | 4 | 5 | 4 | 3 | 5 |
| FE13 | 5 | 5 | 2 | 3 | 4 | 3 | 4 | 5 | 4 | 5 | 4 | 3 | 5 |
| FE18 | 3 | 5 | 4 | 3 | 4 | 5 | 4 | 5 | 4 | 5 | 4 | 3 | 4 |
| FE33 | 3 | 4 | 4 | 3 | 5 | 5 | 4 | 5 | 5 | 5 | 4 | 3 | 5 |
| FE37 | 4 | 4 | 2 | 3 | 5 | 3 | 3 | 4 | 4 | 5 | 4 | 3 | 3 |
| FE51 | 4 | 4 | 4 | 3 | 4 | 3 | 3 | 4 | 4 | 5 | 4 | 3 | 5 |
| FE57 | 5 | 4 | 3 | 4 | 4 | 5 | 3 | 5 | 5 | 5 | 4 | 3 | 4 |
| FS34 | 5 | 4 | 5 | 4 | 5 | 5 | 5 | 5 | 4 | 5 | 4 | 5 | 5 |
| FC1 | 5 | 5 | 4 | 5 | 3 | 5 | 5 | 4 | 3 | 4 | 4 | 4 | 2 |
| FC4 | 5 | 4 | 4 | 5 | 4 | 5 | 5 | 5 | 4 | 5 | 5 | 4 | 1 |
| FC37 | 5 | 4 | 5 | 5 | 4 | 4 | 5 | 3 | 4 | 5 | 5 | 5 | 5 |
| FC38 | 5 | 5 | 3 | 5 | 5 | 5 | 5 | 5 | 3 | 5 | 4 | 5 | 5 |
| FC42 | 5 | 4 | 4 | 5 | 5 | 5 | 5 | 5 | 5 | 5 | 5 | 5 | 5 |
| FC63 | 5 | 4 | 4 | 4 | 4 | 5 | 4 | 5 | 4 | 5 | 5 | 4 | 5 |
| FC64 | 5 | 4 | 4 | 4 | 4 | 3 | 4 | 5 | 4 | 5 | 4 | 4 | 4 |
| FC18 | 5 | 4 | 4 | 4 | 4 | 3 | 4 | 4 | 3 | 4 | 4 | 5 | 3 |
| FC66 | 4 | 3 | 4 | 3 | 4 | 5 | 4 | 4 | 5 | 5 | 5 | 4 | 5 |
| FC67 | 5 | 4 | 4 | 3 | 4 | 5 | 3 | 5 | 4 | 5 | 4 | 5 | 5 |
| FS10 | 5 | 3 | 4 | 5 | 4 | 4 | 5 | 4 | 4 | 5 | 4 | 4 | 5 |
| FC71 | 5 | 4 | 4 | 4 | 4 | 5 | 5 | 5 | 4 | 5 | 4 | 5 | 5 |
| FS44 | 5 | 4 | 5 | 5 | 5 | 5 | 5 | 4 | 3 | 5 | 3 | 4 | 4 |
| FC32 | 5 | 4 | 5 | 4 | 5 | 5 | 5 | 4 | 3 | 5 | 4 | 4 | 5 |
| FS6 | 5 | 4 | 3 | 4 | 5 | 5 | 5 | 4 | 3 | 5 | 4 | 3 | 4 |
| FS20 | 5 | 4 | 4 | 4 | 5 | 5 | 5 | 4 | 4 | 5 | 4 | 5 | 4 |
| FC2 | 5 | 4 | 4 | 3 | 4 | 5 | 5 | 4 | 4 | 5 | 4 | 3 | 5 |
| FC5 | 5 | 4 | 4 | 5 | 4 | 5 | 5 | 5 | 4 | 5 | 5 | 5 | 5 |
| FC6 | 5 | 3 | 4 | 5 | 4 | 5 | 5 | 5 | 4 | 5 | 4 | 5 | 4 |
| FC10 | 5 | 4 | 4 | 5 | 4 | 5 | 5 | 5 | 4 | 5 | 4 | 5 | 4 |
| FC11 | 5 | 4 | 3 | 5 | 4 | 5 | 5 | 4 | 4 | 5 | 2 | 4 | 5 |
| FC16 | 5 | 4 | 3 | 5 | 5 | 5 | 5 | 4 | 4 | 4 | 3 | 4 | 5 |
| FC56 | 5 | 4 | 3 | 5 | 4 | 3 | 5 | 5 | 4 | 4 | 1 | 4 | 5 |
| FC84 | 5 | 4 | 4 | 5 | 4 | 5 | 5 | 5 | 3 | 4 | 5 | 4 | 4 |

**Table S6** Descriptive statistics for all factors

- The table shows the descriptive statistics of the questionnaire survey resulted from Minitab.

| Variable | Total | Mean | StDev | Variance | Sum | Sum of | Min. | Median | Max. | Range | Skewness | Kurtosis |
| --- | --- | --- | --- | --- | --- | --- | --- | --- | --- | --- | --- | --- |
|  | Count |  |  |  |  | Squares |  |  |  |  |  |  |
| FS13 | 100 | 4.13 | 0.8246 | 0.6799 | 413 | 1773 | 1 | 4 | 5 | 4 | -0.91 | 1.18 |
| FS19 | 100 | 4.04 | 0.803 | 0.6448 | 404 | 1696 | 2 | 4 | 5 | 3 | -0.55 | -0.1 |
| FS47 | 100 | 4.14 | 0.8043 | 0.6469 | 414 | 1778 | 1 | 4 | 5 | 4 | -1.21 | 2.36 |
| FC3 | 100 | 3.95 | 0.8454 | 0.7146 | 395 | 1631 | 1 | 4 | 5 | 4 | -0.62 | 0.49 |
| FE56 | 100 | 3.77 | 0.8022 | 0.6435 | 377 | 1485 | 1 | 4 | 5 | 4 | -0.76 | 0.99 |
| FC89 | 100 | 4.33 | 0.7255 | 0.5264 | 433 | 1927 | 3 | 4 | 5 | 2 | -0.6 | -0.88 |
| FC91 | 100 | 3.56 | 0.8913 | 0.7943 | 356 | 1346 | 2 | 3 | 5 | 3 | 0.3 | -0.8 |
| FS1 | 100 | 4.17 | 0.7528 | 0.5668 | 417 | 1795 | 2 | 4 | 5 | 3 | -0.44 | -0.64 |
| FS2 | 100 | 4.31 | 0.8127 | 0.6605 | 431 | 1923 | 2 | 4 | 5 | 3 | -1.09 | 0.71 |
| FS4 | 100 | 3.96 | 0.8399 | 0.7055 | 396 | 1638 | 2 | 4 | 5 | 3 | -0.65 | 0.08 |
| FS7 | 100 | 4 | 0.8646 | 0.7475 | 400 | 1674 | 2 | 4 | 5 | 3 | -0.38 | -0.75 |
| FS37 | 100 | 4.41 | 0.712 | 0.507 | 441 | 1995 | 2 | 5 | 5 | 3 | -1.3 | 2.1 |
| FS38 | 100 | 3.53 | 0.904 | 0.8173 | 353 | 1327 | 1 | 3.5 | 5 | 4 | -0.09 | -0.35 |
| FS45 | 100 | 3.92 | 0.7873 | 0.6198 | 392 | 1598 | 2 | 4 | 5 | 3 | -0.49 | 0.04 |
| FC8 | 100 | 4.47 | 0.6428 | 0.4132 | 447 | 2039 | 2 | 5 | 5 | 3 | -1.05 | 1.1 |
| FC12 | 100 | 3.79 | 0.868 | 0.7534 | 379 | 1511 | 1 | 4 | 5 | 4 | -0.52 | 0.22 |
| FC57 | 100 | 3.91 | 0.9222 | 0.8504 | 391 | 1613 | 1 | 4 | 5 | 4 | -0.76 | 0.65 |
| FC70 | 100 | 4.27 | 0.7086 | 0.5021 | 427 | 1873 | 3 | 4 | 5 | 2 | -0.44 | -0.91 |
| FE20 | 100 | 4.22 | 0.7328 | 0.537 | 422 | 1834 | 2 | 4 | 5 | 3 | -0.53 | -0.41 |
| FE21 | 100 | 3.86 | 0.943 | 0.8893 | 386 | 1578 | 1 | 4 | 5 | 4 | -0.6 | -0.1 |
| FE28 | 100 | 4.13 | 0.7608 | 0.5789 | 413 | 1763 | 3 | 4 | 5 | 2 | -0.22 | -1.23 |
| FE31 | 100 | 3.85 | 0.716 | 0.5126 | 385 | 1533 | 2 | 4 | 5 | 3 | -0.28 | 0.02 |
| FE47 | 100 | 3.93 | 0.7555 | 0.5708 | 393 | 1601 | 2 | 4 | 5 | 3 | -0.17 | -0.55 |
| FE48 | 100 | 3.94 | 0.7361 | 0.5418 | 394 | 1606 | 2 | 4 | 5 | 3 | -0.22 | -0.36 |
| FC14 | 100 | 4.27 | 0.8022 | 0.6435 | 427 | 1887 | 2 | 4 | 5 | 3 | -0.89 | 0.19 |
| FC68 | 100 | 4.14 | 0.7524 | 0.5661 | 414 | 1770 | 3 | 4 | 5 | 2 | -0.24 | -1.19 |
| FE15 | 100 | 4.29 | 0.7148 | 0.511 | 429 | 1891 | 3 | 4 | 5 | 2 | -0.49 | -0.91 |
| FE43 | 100 | 4.09 | 0.8539 | 0.7292 | 409 | 1745 | 2 | 4 | 5 | 3 | -0.37 | -1.04 |
| FC15 | 100 | 3.62 | 0.9404 | 0.8844 | 362 | 1398 | 1 | 4 | 5 | 4 | -0.06 | -0.56 |
| FE14 | 100 | 4.03 | 0.8221 | 0.6759 | 403 | 1691 | 2 | 4 | 5 | 3 | -0.5 | -0.32 |
| FE27 | 100 | 3.71 | 1.104 | 1.218 | 371 | 1497 | 2 | 4 | 5 | 3 | -0.14 | -1.36 |
| FE11 | 100 | 3.97 | 1.029 | 1.06 | 397 | 1681 | 1 | 4 | 5 | 4 | -0.79 | -0.01 |
| FE39 | 100 | 4.16 | 0.8005 | 0.6408 | 416 | 1794 | 2 | 4 | 5 | 3 | -0.78 | 0.28 |
| FE64 | 100 | 3.81 | 0.9287 | 0.8625 | 381 | 1537 | 1 | 4 | 5 | 4 | -0.3 | -0.43 |
| FS3 | 100 | 4.06 | 0.7762 | 0.6024 | 406 | 1708 | 2 | 4 | 5 | 3 | -0.5 | -0.13 |
| FE60 | 100 | 4.01 | 0.7849 | 0.6161 | 401 | 1669 | 2 | 4 | 5 | 3 | -0.53 | 0.02 |
| FE62 | 100 | 3.88 | 0.8678 | 0.7531 | 388 | 1580 | 1 | 4 | 5 | 4 | -0.52 | 0.19 |
| FE44 | 100 | 3.93 | 0.9018 | 0.8132 | 393 | 1625 | 1 | 4 | 5 | 4 | -0.7 | 0.29 |
| FS8 | 100 | 4.07 | 0.7143 | 0.5102 | 407 | 1707 | 3 | 4 | 5 | 2 | -0.1 | -1.01 |
| FC78 | 100 | 3.88 | 0.7821 | 0.6117 | 388 | 1566 | 2 | 4 | 5 | 3 | -0.17 | -0.54 |
| FE1 | 100 | 4.34 | 0.6547 | 0.4287 | 434 | 1926 | 3 | 4 | 5 | 2 | -0.49 | -0.69 |
| FE53 | -100 | 3.96 | 0.7236 | 0.5236 | 396 | 1620 | 2 | 4 | 5 | 3 | -0.27 | -0.2 |
| FC58 | 100 | 3.92 | 0.837 | 0.7006 | 392 | 1606 | 1 | 4 | 5 | 4 | -0.48 | 0.29 |
| FE2 | 100 | 4.08 | 0.8249 | 0.6804 | 408 | 1732 | 1 | 4 | 5 | 4 | -0.81 | 0.98 |
| FE5 | 100 | 4.15 | 0.7571 | 0.5732 | 415 | 1779 | 1 | 4 | 5 | 4 | -0.83 | 1.62 |
| FE7 | 100 | 4.03 | 0.7029 | 0.494 | 403 | 1673 | 2 | 4 | 5 | 3 | -0.4 | 0.18 |
| FE12 | 100 | 4.12 | 0.8908 | 0.7935 | 412 | 1776 | 1 | 4 | 5 | 4 | -0.76 | 0.22 |
| FE13 | 100 | 3.72 | 0.8537 | 0.7289 | 372 | 1456 | 2 | 4 | 5 | 3 | -0.12 | -0.64 |
| FE18 | 100 | 4.03 | 0.8343 | 0.6961 | 403 | 1693 | 1 | 4 | 5 | 4 | -0.48 | 0.09 |
| FE33 | 100 | 4.17 | 0.7661 | 0.587 | 417 | 1797 | 2 | 4 | 5 | 3 | -0.44 | -0.73 |
| FE37 | 100 | 3.71 | 0.868 | 0.7534 | 371 | 1451 | 2 | 4 | 5 | 3 | -0.06 | -0.74 |
| FE51 | 100 | 3.63 | 0.7997 | 0.6395 | 363 | 1381 | 2 | 4 | 5 | 3 | 0.05 | -0.51 |
| FE57 | 100 | 4.01 | 0.7177 | 0.5151 | 401 | 1659 | 2 | 4 | 5 | 3 | -0.18 | -0.54 |
| FS34 | 100 | 4.42 | 0.7545 | 0.5693 | 442 | 2010 | 1 | 5 | 5 | 4 | -1.74 | 4.49 |
| FC1 | 100 | 3.46 | 0.9473 | 0.8974 | 346 | 1286 | 1 | 3 | 5 | 4 | -0.1 | -0.27 |
| FC4 | 100 | 3.92 | 0.849 | 0.7208 | 392 | 1608 | 1 | 4 | 5 | 4 | -0.75 | 0.77 |
| FC37 | 100 | 4.07 | 0.7818 | 0.6112 | 407 | 1717 | 2 | 4 | 5 | 3 | -0.51 | -0.17 |
| FC38 | 100 | 3.87 | 0.9914 | 0.9829 | 387 | 1595 | 2 | 4 | 5 | 3 | -0.5 | -0.77 |
| FC42 | 100 | 4.48 | 0.6273 | 0.3935 | 448 | 2046 | 2 | 5 | 5 | 3 | -1.05 | 1.28 |
| FC63 | 100 | 4.53 | 0.6106 | 0.3728 | 453 | 2089 | 3 | 5 | 5 | 2 | -0.93 | -0.12 |
| FC64 | 100 | 3.93 | 0.6553 | 0.4294 | 393 | 1587 | 3 | 4 | 5 | 2 | 0.07 | -0.63 |
| FC18 | 100 | 3.98 | 0.8406 | 0.7067 | 398 | 1654 | 1 | 4 | 5 | 4 | -0.69 | 0.66 |
| FC66 | 100 | 4.18 | 0.7962 | 0.6339 | 418 | 1810 | 2 | 4 | 5 | 3 | -0.7 | -0.03 |
| FC67 | 100 | 4.34 | 0.6231 | 0.3883 | 434 | 1922 | 3 | 4 | 5 | 2 | -0.39 | -0.64 |
| FS10 | 100 | 4.23 | 0.8511 | 0.7243 | 423 | 1861 | 1 | 4 | 5 | 4 | -1.37 | 2.74 |
| FC71 | 100 | 4.43 | 0.6237 | 0.389 | 443 | 2001 | 3 | 4.5 | 5 | 2 | -0.62 | -0.54 |
| FS44 | 100 | 3.89 | 0.7507 | 0.5635 | 389 | 1569 | 2 | 4 | 5 | 3 | -0.4 | 0.07 |
| FC32 | 100 | 4.1 | 0.7177 | 0.5152 | 410 | 1732 | 2 | 4 | 5 | 3 | -0.32 | -0.44 |
| FS6 | 100 | 3.73 | 0.9195 | 0.8456 | 373 | 1475 | 1 | 4 | 5 | 4 | -0.23 | -0.4 |
| FS20 | 100 | 4.04 | 0.7236 | 0.5236 | 404 | 1684 | 2 | 4 | 5 | 3 | -0.55 | 0.46 |
| FC2 | 100 | 4.01 | 0.7849 | 0.6161 | 401 | 1669 | 1 | 4 | 5 | 4 | -0.53 | 0.69 |
| FC5 | 100 | 4.22 | 0.7464 | 0.5572 | 422 | 1836 | 2 | 4 | 5 | 3 | -0.53 | -0.51 |
| FC6 | 100 | 4.07 | 0.7555 | 0.5708 | 407 | 1713 | 2 | 4 | 5 | 3 | -0.4 | -0.32 |
| FC10 | 100 | 3.87 | 0.8122 | 0.6597 | 387 | 1563 | 1 | 4 | 5 | 4 | -0.56 | 0.68 |
| FC11 | 100 | 3.81 | 0.9287 | 0.8625 | 381 | 1537 | 1 | 4 | 5 | 4 | -0.54 | 0.24 |
| FC16 | 100 | 3.58 | 0.9554 | 0.9127 | 358 | 1372 | 1 | 4 | 5 | 4 | -0.27 | -0.22 |
| FC56 | 100 | 3.59 | 1.083 | 1.174 | 359 | 1405 | 1 | 4 | 5 | 4 | -0.53 | -0.18 |
| FC84 | 100 | 4.4 | 0.7785 | 0.6061 | 440 | 1996 | 1 | 5 | 5 | 4 | -1.49 | 3.02 |

**Table S7** Relative Importance Index (RII)

- The table shows the values of the Relative Importance Index for the analyzed factors, indicating their level of importance.

| Factor | Count of choice (1) | Count of choice (2) | Count of choice (3) | Count of choice (4) | Count of choice (5) | RII | Level of importance |
| --- | --- | --- | --- | --- | --- | --- | --- |
| FS13 | 1 | 2 | 16 | 45 | 36 | 0.826 | H |
| FS19 | 0 | 4 | 18 | 48 | 30 | 0.808 | H |
| FS47 | 1 | 4 | 8 | 54 | 33 | 0.828 | H |
| FC3 | 1 | 3 | 23 | 46 | 27 | 0.790 | H-M |
| FE56 | 1 | 6 | 22 | 57 | 14 | 0.754 | H-M |
| FC89 | 0 | 0 | 15 | 37 | 48 | 0.866 | H |
| FC91 | 0 | 8 | 47 | 26 | 19 | 0.712 | H-M |
| FS1 | 0 | 1 | 18 | 44 | 37 | 0.834 | H |
| FS2 | 0 | 4 | 10 | 37 | 49 | 0.862 | H |
| FS4 | 0 | 7 | 16 | 51 | 26 | 0.792 | H-M |
| FS7 | 0 | 4 | 25 | 38 | 33 | 0.800 | H |
| FS37 | 0 | 3 | 4 | 42 | 51 | 0.882 | H |
| FS38 | 1 | 10 | 39 | 35 | 15 | 0.706 | H-M |
| FS45 | 0 | 5 | 20 | 53 | 22 | 0.784 | H-M |
| FC8 | 0 | 1 | 5 | 40 | 54 | 0.894 | H |
| FC12 | 1 | 6 | 26 | 47 | 20 | 0.758 | H-M |
| FC57 | 2 | 4 | 23 | 43 | 28 | 0.782 | H-M |
| FC70 | 0 | 0 | 15 | 43 | 42 | 0.854 | H |
| FE20 | 0 | 1 | 15 | 45 | 39 | 0.844 | H |
| FE21 | 1 | 8 | 22 | 42 | 27 | 0.772 | H-M |
| FE28 | 0 | 0 | 23 | 41 | 36 | 0.826 | H |
| FE31 | 0 | 3 | 25 | 56 | 16 | 0.770 | H-M |
| FE47 | 0 | 2 | 26 | 49 | 23 | 0.786 | H-M |
| FE48 | 0 | 2 | 24 | 52 | 22 | 0.788 | H-M |
| FC14 | 0 | 3 | 13 | 38 | 46 | 0.854 | H |
| FC68 | 0 | 0 | 22 | 42 | 36 | 0.828 | H |
| FE15 | 0 | 0 | 15 | 41 | 44 | 0.858 | H |
| FE43 | 0 | 2 | 26 | 33 | 39 | 0.818 | H |
| FC15 | 1 | 8 | 40 | 30 | 21 | 0.724 | H-M |
| FE14 | 0 | 4 | 20 | 45 | 31 | 0.806 | H |
| FE27 | 0 | 16 | 31 | 19 | 34 | 0.742 | H-M |
| FE11 | 2 | 7 | 21 | 32 | 38 | 0.794 | H-M |
| FE39 | 0 | 4 | 13 | 46 | 37 | 0.832 | H |
| FE64 | 1 | 5 | 33 | 34 | 27 | 0.762 | H-M |
| FS3 | 0 | 3 | 18 | 49 | 30 | 0.812 | H |
| FE60 | 0 | 4 | 18 | 51 | 27 | 0.802 | H |
| FE62 | 1 | 4 | 26 | 44 | 25 | 0.776 | H-M |
| FE44 | 1 | 6 | 20 | 45 | 28 | 0.786 | H-M |
| FS8 | 0 | 0 | 22 | 49 | 29 | 0.814 | H |
| FC78 | 0 | 3 | 28 | 47 | 22 | 0.776 | H-M |
| FE1 | 0 | 0 | 10 | 46 | 44 | 0.868 | H |
| FE53 | 0 | 2 | 22 | 54 | 22 | 0.792 | H-M |
| FC58 | 1 | 2 | 27 | 44 | 26 | 0.784 | H-M |
| FE2 | 1 | 2 | 18 | 46 | 33 | 0.816 | H |
| FE5 | 1 | 0 | 16 | 49 | 34 | 0.830 | H |
| FE7 | 0 | 2 | 17 | 57 | 24 | 0.806 | H |
| FE12 | 1 | 2 | 22 | 34 | 41 | 0.824 | H |
| FE13 | 0 | 7 | 33 | 41 | 19 | 0.744 | H-M |
| FE18 | 1 | 0 | 27 | 39 | 33 | 0.806 | H |
| FE33 | 0 | 1 | 19 | 42 | 38 | 0.834 | H |
| FE37 | 0 | 7 | 35 | 38 | 20 | 0.742 | H-M |
| FE51 | 0 | 6 | 39 | 41 | 14 | 0.726 | H-M |
| FE57 | 0 | 1 | 22 | 52 | 25 | 0.802 | H |
| FS34 | 1 | 2 | 4 | 40 | 53 | 0.884 | H |
| FC1 | 2 | 11 | 41 | 31 | 15 | 0.692 | H-M |
| FC4 | 1 | 5 | 19 | 51 | 24 | 0.784 | H-M |
| FC37 | 0 | 3 | 18 | 48 | 31 | 0.814 | H |
| FC38 | 0 | 12 | 20 | 37 | 31 | 0.774 | H-M |
| FC42 | 0 | 1 | 4 | 41 | 54 | 0.896 | H |
| FC63 | 0 | 0 | 6 | 35 | 59 | 0.906 | H |
| FC64 | 0 | 0 | 25 | 57 | 18 | 0.786 | H-M |
| FC18 | 1 | 3 | 21 | 47 | 28 | 0.796 | H-M |
| FC66 | 0 | 3 | 15 | 43 | 39 | 0.836 | H |
| FC67 | 0 | 0 | 8 | 50 | 42 | 0.868 | H |
| FS10 | 2 | 1 | 12 | 42 | 43 | 0.846 | H |
| FC71 | 0 | 0 | 7 | 43 | 50 | 0.886 | H |
| FS44 | 0 | 4 | 22 | 55 | 19 | 0.778 | H-M |
| FC32 | 0 | 1 | 18 | 51 | 30 | 0.820 | H |
| FS6 | 1 | 6 | 35 | 35 | 23 | 0.746 | H-M |
| FS20 | 0 | 3 | 15 | 57 | 25 | 0.808 | H |
| FC2 | 1 | 0 | 24 | 47 | 28 | 0.802 | H |
| FC5 | 0 | 1 | 16 | 43 | 40 | 0.844 | H |
| FC6 | 0 | 2 | 19 | 49 | 30 | 0.814 | H |
| FC10 | 1 | 3 | 25 | 50 | 21 | 0.774 | H-M |
| FC11 | 2 | 4 | 30 | 39 | 25 | 0.762 | H-M |
| FC16 | 2 | 9 | 36 | 35 | 18 | 0.716 | H-M |
| FC56 | 5 | 9 | 30 | 34 | 22 | 0.718 | H-M |
| FC84 | 1 | 1 | 9 | 35 | 54 | 0.880 | H |

**Table S8** Final factors as per the correlation analysis

- The table shows the resulted factors out of the four correlations developed by applying the Principal Component Analysis. As shown, the environmental factors are the most-rated factors (i.e., 9 factors out of 11).

| Category | ID | Factor |
| --- | --- | --- |
| Economic | FC10 | Encourage investments directed to each individual project that supports community goals. |
| Environmental | FE11 | Avoid, reduce, and compensate the loss of natural (stream and terrestrial) habitat of flora and fauna caused by project construction, and protect natural habitat, and high-quality environmental and aquatic resources. |
|  | FE18 | Reduce emissions from construction equipment/vehicles by using emission reduction exhaust retrofit and alternative fuels, such as biodiesel fuel and hybrid diesel-electric engines. |
|  | FE28 | Adequate demolition plan on hazardous materials and waste reduction or recycling and give special treatment given to toxic materials, heavy metals, and radioactive chemicals. |
|  | FE39 | Provide, preserve, and increase universal access to safe, accessible, green, and public spaces for all, and confront desertification, in addition to restoring degraded land and soil. |
|  | FE44 | Protect the site soil from pollution. |
|  | FE53 | Increase the environmental awareness for the public of preserving the environment and mitigate climate change through various educational and training programs. |
|  | FE57 | Developing a periodic assessment of highway operation and maintenance in terms of air and water quality, emissions, and noise impacts. |
|  | FE60 | Improve water quality of stormwater runoff by treating rainwater as a resource rather than as a waste product and develop an effective flood warning protocol. |
|  | FE62 | Include an erosion and sedimentation control plan for construction activities and use recycled materials for maintenance and repair of banks and steep slopes to improve water quality. |
| Social | FS8 | Develop risk assessment to identify and reduce any disasters or future risks to the public and road users. |

**Table S9** Results of Pareto Principle (20/80 Rule)

- The table shows the results of applying Pareto Principle. The red-colored factors are omitted as their cumulative percentage more than 80%, as Pareto Principle states that only 20% of a phenomenon's causes (black-colored factors) may express 80% of its effects. The number of considered factors are high due to the convergence of RII values.

| Factor | RII | % | Cum. % | Factor | RII | % | Cum. % | Factor | RII | % | Cum. % |
| --- | --- | --- | --- | --- | --- | --- | --- | --- | --- | --- | --- |
| FS34 | 0.884 | 6.401 | 6.401 | FC5 | 0.844 | 3.273 | 33.920 | FE33 | 0.834 | 3.609 | 14.732 |
| FS37 | 0.882 | 6.387 | 12.788 | FC66 | 0.836 | 3.242 | 37.162 | FE39 | 0.832 | 3.601 | 18.333 |
| FS2 | 0.862 | 6.242 | 19.030 | FC68 | 0.828 | 3.211 | 40.372 | FE5 | 0.830 | 3.592 | 21.925 |
| FS10 | 0.846 | 6.126 | 25.156 | FC32 | 0.820 | 3.180 | 43.552 | FE28 | 0.826 | 3.575 | 25.500 |
| FS1 | 0.834 | 6.039 | 31.195 | FC37 | 0.814 | 3.156 | 46.708 | FE12 | 0.824 | 3.566 | 29.066 |
| FS47 | 0.828 | 5.996 | 37.190 | FC6 | 0.814 | 3.156 | 49.864 | FE43 | 0.818 | 3.540 | 32.606 |
| FS13 | 0.826 | 5.981 | 43.172 | FC2 | 0.802 | 3.110 | 52.974 | FE2 | 0.816 | 3.532 | 36.138 |
| FS8 | 0.814 | 5.894 | 49.066 | FC18 | 0.796 | 3.086 | 56.060 | FE14 | 0.806 | 3.488 | 39.626 |
| FS3 | 0.812 | 5.880 | 54.946 | FC3 | 0.790 | 3.063 | 59.124 | FE7 | 0.806 | 3.488 | 43.114 |
| FS19 | 0.808 | 5.851 | 60.797 | FC64 | 0.786 | 3.048 | 62.171 | FE18 | 0.806 | 3.488 | 46.603 |
| FS20 | 0.808 | 5.851 | 66.647 | FC58 | 0.784 | 3.040 | 65.211 | FE60 | 0.802 | 3.471 | 50.074 |
| FS7 | 0.800 | 5.793 | 72.440 | FC4 | 0.784 | 3.040 | 68.251 | FE57 | 0.802 | 3.471 | 53.545 |
| FS4 | 0.792 | 5.735 | 78.175 | FC57 | 0.782 | 3.032 | 71.283 | FE11 | 0.794 | 3.436 | 56.981 |
| FS45 | 0.784 | 5.677 | 83.852 | FC78 | 0.776 | 3.009 | 74.292 | FE53 | 0.792 | 3.428 | 60.409 |
| FS44 | 0.778 | 5.634 | 89.486 | FC38 | 0.774 | 3.001 | 77.294 | FE48 | 0.788 | 3.410 | 63.819 |
| FS6 | 0.746 | 5.402 | 94.888 | FC10 | 0.774 | 3.001 | 80.295 | FE47 | 0.786 | 3.402 | 67.221 |
| FS38 | 0.706 | 5.112 | 100.000 | FC11 | 0.762 | 2.955 | 83.249 | FE44 | 0.786 | 3.402 | 70.622 |
| Sum | 13.810 | 100 |  | FC12 | 0.758 | 2.939 | 86.188 | FE62 | 0.776 | 3.358 | 73.981 |
| FC63 | 0.906 | 3.513 | 3.513 | FC15 | 0.724 | 2.807 | 88.996 | FE21 | 0.772 | 3.341 | 77.322 |
| FC42 | 0.896 | 3.474 | 6.987 | FC56 | 0.718 | 2.784 | 91.780 | FE31 | 0.770 | 3.332 | 80.654 |
| FC8 | 0.894 | 3.466 | 10.454 | FC16 | 0.716 | 2.776 | 94.556 | FE64 | 0.762 | 3.298 | 83.952 |
| FC71 | 0.886 | 3.435 | 13.889 | FC91 | 0.712 | 2.761 | 97.317 | FE56 | 0.754 | 3.263 | 87.215 |
| FC84 | 0.880 | 3.412 | 17.301 | FC1 | 0.692 | 2.683 | 100.000 | FE13 | 0.744 | 3.220 | 90.435 |
| FC67 | 0.868 | 3.366 | 20.667 | Sum | 25.790 | 100 |  | FE27 | 0.742 | 3.211 | 93.647 |
| FC89 | 0.866 | 3.358 | 24.025 | FE1 | 0.868 | 3.757 | 3.757 | FE37 | 0.742 | 3.211 | 96.858 |
| FC70 | 0.854 | 3.311 | 27.336 | FE15 | 0.858 | 3.713 | 7.470 | FE51 | 0.726 | 3.142 | 100.000 |
| FC14 | 0.854 | 3.311 | 30.648 | FE20 | 0.844 | 3.653 | 11.123 | Sum | 23.106 | 100 |  |

**Table S10** AHP analysis for the social aspect

- This process discusses the calculation of the weights for social factors, developing the column normalization and the consistency check to ensure that the data of the responses are consistent.

| **ID** | **FS2** | **FS3** | **FS37** | **Column Vector** | **W (PV)** |
| --- | --- | --- | --- | --- | --- |
| **FS2** | 0.47 | 0.50 | 0.45 | 1.42 | 0.47 |
| **FS3** | 0.16 | 0.17 | 0.19 | 0.52 | 0.17 |
| **FS37** | 0.37 | 0.33 | 0.36 | 1.07 | 0.36 |
| **Total** | 1.00 | 1.00 | 1.00 | 3.00 | 1.00 |

| **ID** | **FS2** | **FS3** | **FS37** |  | **W** |  | **W'** |  | **W''** |  | **n = 3** | |
| --- | --- | --- | --- | --- | --- | --- | --- | --- | --- | --- | --- | --- |
| **FS2** | 1.00 | 2.92 | 1.25 | X | 0.47 | = | 1.42 |  | 3.01 |  | **λ max = 3.00361** | |
| **FS3** | 0.34 | 1.00 | 0.51 |  | 0.17 |  | 0.52 |  | 3.00 |  | **CI = 0.0018** | |
| **FS37** | 0.80 | 1.95 | 1.00 |  | 0.36 |  | 1.07 |  | 3.00 |  | **CR = 0.00311** | |
|  |  |  |  |  |  |  |  |  | 9.01 |  |  |  |

**Table S11** AHP analysis for the environmental aspect

- This process discusses the calculation of the weights for environmental factors, developing the column normalization and the consistency check to ensure that the data of the responses are consistent.

| **ID** | **FE1** | **FE5** | **FE15** | **FE33** | **FE39** | **Column Vector** | **W (PV)** |
| --- | --- | --- | --- | --- | --- | --- | --- |
| **FE1** | 0.22 | 0.30 | 0.24 | 0.16 | 0.20 | 1.12 | 0.22 |
| **FE5** | 0.15 | 0.21 | 0.26 | 0.26 | 0.19 | 1.08 | 0.22 |
| **FE15** | 0.21 | 0.18 | 0.22 | 0.25 | 0.27 | 1.13 | 0.23 |
| **FE33** | 0.28 | 0.17 | 0.18 | 0.20 | 0.21 | 1.03 | 0.21 |
| **FE39** | 0.14 | 0.14 | 0.10 | 0.13 | 0.13 | 0.64 | 0.13 |
| **Total** | 1.00 | 1.00 | 1.00 | 1.00 | 1.00 | 5.00 | 1.00 |

| **ID** | **FE1** | **FE5** | **FE15** | **FE33** | **FE39** |  | **W** |  | **W'** |  | **W''** |  | **n = 5** | |
| --- | --- | --- | --- | --- | --- | --- | --- | --- | --- | --- | --- | --- | --- | --- |
| **FE1** | 1.00 | 1.44 | 1.07 | 0.81 | 1.56 |  | 0.22 |  | 1.14 |  | 5.08 |  | **λ max = 5.07185** | |
| **FE5** | 0.69 | 1.00 | 1.17 | 1.28 | 1.52 | X | 0.22 | = | 1.09 |  | 5.07 |  | **CI = 0.01796** | |
| **FE15** | 0.94 | 0.86 | 1.00 | 1.22 | 2.14 |  | 0.23 |  | 1.14 |  | 5.07 |  | **CR = 0.01604** | |
| **FE33** | 1.24 | 0.78 | 0.82 | 1.00 | 1.63 |  | 0.21 |  | 1.05 |  | 5.07 |  |  |  |
| **FE39** | 0.64 | 0.66 | 0.47 | 0.62 | 1.00 |  | 0.13 |  | 0.65 |  | 5.07 |  |  |  |
|  |  |  |  |  |  |  |  |  |  |  | 25.36 |  |  |  |

**Table S12** AHP analysis for the economic aspect

- This process discusses the calculation of weights for economic factors, developing the column normalization and the consistency check to ensure that the data of the responses are consistent.

| **ID** | **FC5** | **FC8** | **FC14** | **FC32** | **FC42** | **FC63** | **FC70** | **FC71** | **FC84** | **FC89** | **Column Vector** | **W (PV)** |
| --- | --- | --- | --- | --- | --- | --- | --- | --- | --- | --- | --- | --- |
| **FC5** | 0.14 | 0.23 | 0.17 | 0.10 | 0.16 | 0.15 | 0.10 | 0.12 | 0.11 | 0.10 | 1.37 | 0.14 |
| **FC8** | 0.06 | 0.09 | 0.11 | 0.14 | 0.11 | 0.10 | 0.10 | 0.11 | 0.09 | 0.10 | 1.01 | 0.10 |
| **FC14** | 0.11 | 0.12 | 0.13 | 0.18 | 0.16 | 0.15 | 0.13 | 0.13 | 0.12 | 0.11 | 1.35 | 0.13 |
| **FC32** | 0.10 | 0.05 | 0.06 | 0.08 | 0.08 | 0.09 | 0.07 | 0.10 | 0.07 | 0.07 | 0.77 | 0.08 |
| **FC42** | 0.09 | 0.08 | 0.08 | 0.10 | 0.10 | 0.12 | 0.13 | 0.10 | 0.11 | 0.11 | 1.01 | 0.10 |
| **FC63** | 0.10 | 0.10 | 0.09 | 0.08 | 0.09 | 0.10 | 0.15 | 0.11 | 0.14 | 0.12 | 1.08 | 0.11 |
| **FC70** | 0.11 | 0.08 | 0.08 | 0.09 | 0.06 | 0.06 | 0.08 | 0.09 | 0.09 | 0.09 | 0.84 | 0.08 |
| **FC71** | 0.12 | 0.10 | 0.12 | 0.08 | 0.12 | 0.11 | 0.10 | 0.12 | 0.14 | 0.13 | 1.14 | 0.11 |
| **FC84** | 0.11 | 0.09 | 0.10 | 0.09 | 0.08 | 0.07 | 0.08 | 0.07 | 0.09 | 0.13 | 0.90 | 0.09 |
| **FC89** | 0.08 | 0.05 | 0.06 | 0.06 | 0.05 | 0.05 | 0.05 | 0.05 | 0.04 | 0.05 | 0.54 | 0.05 |
| **Total** | 1.00 | 1.00 | 1.00 | 1.00 | 1.00 | 1.00 | 1.00 | 1.00 | 1.00 | 1.00 | 10.00 | 1.00 |

| **ID** | **FC5** | **FC8** | **FC14** | **FC32** | **FC42** | **FC63** | **FC70** | **FC71** | **FC84** | **FC89** |  | **W** |  | **W'** |  | **W''** |  | **n = 10** | |
| --- | --- | --- | --- | --- | --- | --- | --- | --- | --- | --- | --- | --- | --- | --- | --- | --- | --- | --- | --- |
| **FC5** | 1.00 | 2.43 | 1.26 | 1.36 | 1.58 | 1.42 | 1.23 | 1.08 | 1.22 | 1.78 |  | 0.14 |  | 1.40 |  | 10.23 |  | **λ max =10.181** | |
| **FC8** | 0.41 | 1.00 | 0.79 | 1.83 | 1.16 | 0.93 | 1.19 | 0.96 | 1.03 | 1.92 |  | 0.10 |  | 1.03 |  | 10.18 |  | **CI = 0.0202** | |
| **FC14** | 0.80 | 1.27 | 1.00 | 2.36 | 1.66 | 1.45 | 1.57 | 1.13 | 1.37 | 2.07 |  | 0.13 |  | 1.37 |  | 10.19 |  | **CR = 0.0136** | |
| **FC32** | 0.73 | 0.55 | 0.42 | 1.00 | 0.77 | 0.90 | 0.81 | 0.90 | 0.83 | 1.30 |  | 0.08 |  | 0.78 |  | 10.17 |  |  |  |
| **FC42** | 0.63 | 0.86 | 0.60 | 1.30 | 1.00 | 1.11 | 1.57 | 0.84 | 1.28 | 2.08 |  | 0.10 |  | 1.03 |  | 10.18 |  |  |  |
| **FC63** | 0.71 | 1.08 | 0.69 | 1.11 | 0.90 | 1.00 | 1.79 | 0.92 | 1.60 | 2.18 | X | 0.11 | = | 1.10 |  | 10.18 |  |  |  |
| **FC70** | 0.81 | 0.84 | 0.64 | 1.24 | 0.64 | 0.56 | 1.00 | 0.82 | 1.03 | 1.61 |  | 0.08 |  | 0.86 |  | 10.17 |  |  |  |
| **FC71** | 0.92 | 1.04 | 0.89 | 1.12 | 1.20 | 1.09 | 1.23 | 1.00 | 1.58 | 2.33 |  | 0.11 |  | 1.16 |  | 10.18 |  |  |  |
| **FC84** | 0.82 | 0.97 | 0.73 | 1.20 | 0.78 | 0.63 | 0.97 | 0.63 | 1.00 | 2.33 |  | 0.09 |  | 0.92 |  | 10.18 |  |  |  |
| **FC89** | 0.56 | 0.52 | 0.48 | 0.77 | 0.48 | 0.46 | 0.62 | 0.43 | 0.43 | 1.00 |  | 0.05 |  | 0.54 |  | 10.18 |  |  |  |
|  |  |  |  |  |  |  |  |  |  |  |  |  |  |  |  | 101.82 |  |  |  |

**Table S13** AHP analysis for the main pillars of sustainability

- This process discusses the calculation of weights for the main pillars of sustainability, developing the column normalization and the consistency check to ensure that the data of the responses are consistent.

| **ID** | **FS** | **FC** | **FE** | **Column Vector** | **W (PV)** |
| --- | --- | --- | --- | --- | --- |
| **FS** | 0.24 | 0.33 | 0.19 | 0.76 | 0.25 |
| **FC** | 0.27 | 0.36 | 0.44 | 1.07 | 0.36 |
| **FE** | 0.49 | 0.31 | 0.37 | 1.17 | 0.39 |
| **Total** | 1.00 | 1.00 | 1.00 | 3.00 | 1.00 |

| **ID** | **FS** | **FC** | **FE** |  | **W** |  | **W'** |  | **W''** |  | **n = 3** | |
| --- | --- | --- | --- | --- | --- | --- | --- | --- | --- | --- | --- | --- |
| **FS** | 1.00 | 0.90 | 0.50 |  | 0.25 |  | 0.77 |  | 3.05 |  | **λ max = 3.06373** | |
| **FC** | 1.11 | 1.00 | 1.18 | X | 0.36 | = | 1.10 |  | 3.07 |  | **CI = 0.03186** | |
| **FE** | 2.01 | 0.85 | 1.00 |  | 0.39 |  | 1.20 |  | 3.08 |  | **CR =** | **0.05494** |
|  |  |  |  |  |  |  |  |  | 9.19 |  |  |  |

**Table S14**  The questions’ weights of HWS RS

- This table shows the calculation of RII values for the developed questions for each factor, which determines the weight/score for each question.

| Factor | Question | Count of choice (1) | Count of choice (2) | Count of choice (3) | Count of choice (4) | Count of choice (5) | RII | Weight |
| --- | --- | --- | --- | --- | --- | --- | --- | --- |
| FS2 | FS2.1 | 0 | 1 | 0 | 8 | 3 | 0.817 | 0.20 |
|  | FS2.2 | 1 | 1 | 4 | 1 | 5 | 0.733 | 0.19 |
|  | FS2.3 | 0 | 0 | 0 | 5 | 7 | 0.917 | 0.23 |
|  | FS2.4 | 1 | 1 | 3 | 4 | 3 | 0.717 | 0.18 |
|  | FS2.5 | 0 | 0 | 3 | 5 | 4 | 0.817 | 0.20 |
| FS3 | FS3.1 | 0 | 1 | 3 | 4 | 4 | 0.783 | 0.23 |
|  | FS3.2 | 0 | 0 | 0 | 4 | 8 | 0.933 | 0.28 |
|  | FS3.3 | 0 | 0 | 4 | 4 | 4 | 0.800 | 0.24 |
|  | FS3.4 | 0 | 0 | 1 | 7 | 4 | 0.850 | 0.25 |
| FS37 | FS37.1 | 0 | 0 | 4 | 2 | 6 | 0.833 | 0.12 |
|  | FS37.2 | 0 | 0 | 1 | 5 | 6 | 0.883 | 0.11 |
|  | FS37.3 | 0 | 0 | 3 | 4 | 5 | 0.833 | 0.12 |
|  | FS37.4 | 0 | 1 | 1 | 5 | 5 | 0.833 | 0.12 |
|  | FS37.5 | 0 | 2 | 3 | 5 | 2 | 0.717 | 0.10 |
|  | FS37.6 | 0 | 3 | 3 | 4 | 2 | 0.683 | 0.10 |
|  | FS37.7 | 0 | 0 | 5 | 6 | 1 | 0.733 | 0.10 |
|  | FS37.8 | 1 | 1 | 5 | 2 | 3 | 0.683 | 0.10 |
|  | FS37.9 | 0 | 0 | 1 | 3 | 8 | 0.917 | 0.13 |
| FE1 | FE1.1 | 0 | 0 | 0 | 7 | 5 | 0.883 | 0.51 |
|  | FE1.2 | 0 | 0 | 2 | 5 | 5 | 0.850 | 0.49 |
| FE5 | FE5.1 | 0 | 1 | 2 | 5 | 4 | 0.800 | 0.35 |
|  | FE5.2 | 0 | 2 | 2 | 4 | 4 | 0.767 | 0.33 |
|  | FE5.3 | 0 | 2 | 2 | 6 | 2 | 0.733 | 0.32 |
| FE15 | FE15.1 | 1 | 1 | 5 | 4 | 1 | 0.650 | 0.44 |
|  | FE15.2 | 0 | 0 | 2 | 6 | 4 | 0.833 | 0.56 |
| FE33 | FE33.1 | 0 | 0 | 1 | 6 | 5 | 0.867 | 0.22 |
|  | FE33.2 | 0 | 0 | 5 | 2 | 5 | 0.800 | 0.20 |
|  | FE33.3 | 0 | 1 | 1 | 8 | 2 | 0.783 | 0.20 |
|  | FE33.4 | 0 | 2 | 4 | 4 | 2 | 0.700 | 0.18 |
|  | FE33.5 | 0 | 2 | 1 | 4 | 5 | 0.800 | 0.20 |
| FE39 | FE39.1 | 0 | 0 | 3 | 5 | 4 | 0.817 | 0.21 |
|  | FE39.2 | 0 | 0 | 4 | 5 | 3 | 0.783 | 0.19 |
|  | FE39.3 | 0 | 0 | 3 | 8 | 1 | 0.767 | 0.19 |
|  | FE39.4 | 0 | 0 | 2 | 8 | 2 | 0.800 | 0.20 |
|  | FE39.5 | 0 | 0 | 2 | 7 | 3 | 0.817 | 0.21 |
| FC5 | FC5.1 | 0 | 0 | 0 | 1 | 11 | 0.983 | 0.22 |
|  | FC5.2 | 0 | 0 | 1 | 4 | 7 | 0.900 | 0.20 |
|  | FC5.3 | 0 | 0 | 0 | 7 | 5 | 0.883 | 0.20 |
|  | FC5.4 | 0 | 0 | 0 | 8 | 4 | 0.867 | 0.18 |
|  | FC5.5 | 0 | 0 | 1 | 5 | 6 | 0.883 | 0.20 |
| FC8 | FC8.1 | 0 | 0 | 0 | 6 | 6 | 0.900 | 0.26 |
|  | FC8.2 | 0 | 1 | 2 | 4 | 5 | 0.817 | 0.24 |
|  | FC8.3 | 0 | 0 | 1 | 8 | 3 | 0.833 | 0.25 |
|  | FC8.4 | 0 | 0 | 1 | 8 | 3 | 0.833 | 0.25 |
| FC14 | FC14.1 | 0 | 0 | 3 | 7 | 2 | 0.783 | 0.33 |
|  | FC14.2 | 0 | 0 | 3 | 6 | 3 | 0.800 | 0.33 |
|  | FC14.3 | 0 | 0 | 2 | 7 | 3 | 0.817 | 0.34 |
| FC32 | FC32.1 | 0 | 1 | 0 | 5 | 6 | 0.867 | 0.25 |
|  | FC32.2 | 0 | 0 | 1 | 3 | 8 | 0.917 | 0.26 |
|  | FC32.3 | 0 | 0 | 1 | 5 | 6 | 0.883 | 0.25 |
|  | FC32.4 | 0 | 0 | 3 | 5 | 4 | 0.817 | 0.24 |
| FC42 | FC42.1 | 0 | 0 | 1 | 5 | 6 | 0.883 | 0.27 |
|  | FC42.2 | 0 | 0 | 3 | 7 | 2 | 0.783 | 0.24 |
|  | FC42.3 | 0 | 0 | 3 | 7 | 2 | 0.783 | 0.24 |
|  | FC42.4 | 0 | 0 | 2 | 4 | 6 | 0.867 | 0.25 |
| FC63 | FC63.1 | 0 | 0 | 1 | 7 | 4 | 0.850 | 0.49 |
|  | FC63.2 | 0 | 0 | 0 | 6 | 6 | 0.900 | 0.51 |
| FC70 | FC70.1 | 0 | 1 | 0 | 4 | 7 | 0.883 | 1.00 |
| FC71 | FC71.1 | 0 | 0 | 0 | 4 | 8 | 0.933 | 0.26 |
|  | FC71.2 | 0 | 0 | 0 | 5 | 7 | 0.917 | 0.25 |
|  | FC71.3 | 0 | 0 | 0 | 5 | 7 | 0.917 | 0.25 |
|  | FC71.4 | 0 | 0 | 1 | 7 | 4 | 0.850 | 0.24 |
| FC84 | FC84.1 | 0 | 0 | 1 | 5 | 6 | 0.883 | 0.51 |
|  | FC84.2 | 0 | 1 | 0 | 6 | 5 | 0.850 | 0.49 |
| FC89 | FC89.1 | 0 | 1 | 5 | 5 | 1 | 0.700 | 0.23 |
|  | FC89.2 | 0 | 1 | 1 | 4 | 6 | 0.850 | 0.25 |
|  | FC89.3 | 0 | 0 | 1 | 6 | 5 | 0.867 | 0.26 |
|  | FC89.4 | 0 | 0 | 2 | 4 | 6 | 0.867 | 0.26 |

**Table S15**  The assessment questions of the HWS RS

- This table shows the developed HWS assessment model, showing each sustainability aspect, the included questions, and the available answers for each question.

| **Economic Factors** | | | | |
| --- | --- | --- | --- | --- |
| **FC5** | | | **Develop a feasibility study to define the capital budget needed for the project** | |
| FC5.1 | | | Has the project team provided a feasibility study including financial, technical, marketing, environmental, and economic data? 🖵 Yes 🖵 No | |
| FC5.2 | | | Has the owner defined the investment sources and payments available for the project?  🖵 Yes 🖵 No | |
| FC5.3 | | | Was a risk assessment incorporated for revenue forecasting? 🖵 Yes 🖵 No | |
| FC5.4 | | | Has the project team estimated the annual budget needed for the maintenance and rehabilitation processes? 🖵 Yes 🖵 No | |
| FC5.5 | | | Has the owner prioritized the suggested projects according to their importance of implementation?  🖵 Yes 🖵 No | |
| **FC8** | | | **Selecting economic, durable, and available materials** | |
| FC8.1 | | | Has the project team considered using more durable alternative materials? 🖵 Yes 🖵 No | |
| FC8.2 | | | Has the owner been informed in case of material shortage to confirm another suitable alternative?  🖵 Yes 🖵 No | |
| FC8.3 | | | Has the contractor succeeded in overcoming the continuous rise of material prices, if any?  🖵 Yes 🖵 No | |
| FC8.4 | | | Were the expected transportation distances to the site determined to be the minimum for the majority of materials? 🖵 Yes 🖵 No | |
| **FC14** | | | **Reduce the consumption of various types of energy, especially fossil fuel, and encourage using renewable energy and define their costs** | |
| FC14.1 | | | To what extent has the project considered strategies to reduce the energy use? | |
|  | | | 🖵 Using High-efficiency traffic signals and luminaries (such as LED) | |
|  | | | 🖵 Using automatic cut-off fixtures for lighting poles | |
|  | | | 🖵 Replacing the lighting signals with reflective signs | |
|  | | | 🖵 Using recycled materials during construction/maintenance (i.e., FDR) | |
| FC14.2 | | | To what extent has the contractor reduced the needed energy and lighting required for the construction site? | |
|  | | | 🖵 0 🖵 < 5% 🖵 5:15% 🖵 15:25% 🖵 25:50% 🖵 > 50% | |
| FC14.3 | | | To what extent has the project met energy needs from renewable resources? | |
|  | | | 🖵 0 🖵 < 25% 🖵 25:50% 🖵 50:75% 🖵 > 75% 🖵 100% | |
| **FC32** | | | **Develop a clear and detailed program for quality management including developing quality control plan (QCP)** | |
| FC32.1 | | | To what extent has the contractor made construction mistakes that needed to be reworked? | |
|  | | | 🖵 0 🖵 < 5% 🖵 5:15% 🖵 15:30% 🖵 30:50% 🖵 > 50% | |
| FC32.2 | | | To what extent have the specifications been written clearly? | |
|  | | | 🖵 Acceptable 🖵 Good 🖵 Very good 🖵 Excellent | |
| FC32.3 | | | To what extent has the quality control team been qualified? | |
|  | | | 🖵 Acceptable 🖵 Good 🖵 Very good 🖵 Excellent | |
| FC32.4 | | | Has the contractor presented a corrective action plan, if needed? 🖵 Yes 🖵 No | |
| **FC42** | | | **Design pavement according to the regional conditions according to the soil type and traffic volume** | |
| FC42.1 | | | To what extent has the contractor committed to the prepared soil boring report and the approved traffic studies? | |
|  | | | 🖵 Acceptable 🖵 Good 🖵 Very good 🖵 Excellent | |
| FC42.2 | | | Has the contractor prepared another soil boring report to verify the owner’s one? 🖵 Yes 🖵 No | |
| FC42.3 | | | To what extent has the project considered the nearby constraints during the planning, design, and construction stages?  🖵 Acceptable 🖵 Good 🖵 Very good 🖵 Excellent | |
| FC42.4 | | | To what extent has the project considered the runoff discharge? | |
|  | | | 🖵 Acceptable 🖵 Good 🖵 Very good 🖵 Excellent | |
| **FC63** | | | **Design long-life pavement** | |
| FC63.1 | | | To what extent has the project ensured the reliability of the developed traffic data? | |
|  | | | 🖵 Acceptable 🖵 Good 🖵 Very good 🖵 Excellent | |
| FC63.2 | | | To what extent has the contractor committed to the approved designs and specifications? | |
|  | | | 🖵 Acceptable 🖵 Good 🖵 Very good 🖵 Excellent | |
| **FC70** | | | **Create, modify, and use specifications that allow for sustainability best practices and achieve the efficient use** | |
| FC70.1 | | | Have the specifications provided the data resources and methods that explicitly address sustainability principles and allow sustainability best practices? 🖵 Yes 🖵 No | |
| **FC71** | | | **Plan for long-term monitoring and maintenance** | |
| FC71.1 | | | Is there a clear and comprehensive plan in place for long-term monitoring and maintenance of the completed project? 🖵 Yes 🖵 No | |
| FC71.2 | | | Have sufficient resources and staff been assigned for long-term monitoring and maintenance of the completed project? 🖵 Yes 🖵 No | |
| FC71.3 | | | To what extent has the monitoring and maintenance plan been considered with operations and maintenance staff? | |
|  | | | 🖵 Acceptable 🖵 Good 🖵 Very good 🖵 Excellent | |
| FC71.4 | | | Is there a plan in-place to re-evaluate and modify the maintenance plan based on the monitored data? 🖵 Yes 🖵 No | |
| **FC84** | | | **Develop life‐Cycle Cost Analyses (LCCA) and Benefit‐Cost Analysis (BCA) to apply the best alternatives** | |
| FC84.1 | | | Has the project team developed a life-cycle cost analysis to predict the project costs and perform short- and long-term budget forecasting? 🖵 Yes 🖵 No | |
| FC84.2 | | | Has the project team applied Value Engineering to compare alternatives for at least one major project component? 🖵 Yes 🖵 No | |
| **FC89** | | | **Accommodate multi-modal transportation uses (freight vehicles, pedestrians, ridesharing and bicyclists), including providing new intermodal connections** | |
| FC89.1 | | | Has the project provided a multi-modal transportation system? 🖵 Yes 🖵 No | |
| FC89.2 | | | To what extent has the project considered measures to reduce accidents? | |
|  | | | 🖵 Providing warning and traffic signals 🖵 Providing calming lanes | |
|  | | | 🖵 Providing buffers between large vehicles and light vehicle traffic 🖵 Providing shoulders | |
| FC89.3 | | | Has the project been designed for high-occupancy/carpool and articulated vehicles? 🖵 Yes 🖵 No | |
| FC89.4 | | | Has the project promoted a reduction in vehicle trips by encouraging increased use of public transit and ride-sharing to reduce the operational expenses? 🖵 Yes 🖵 No | |
| **Environmental Factors** | | | | |
| **FE1** | | | **Apply life cycle assessment** | |
| FE1.1 | | | Has the contractor submitted a certified LCA/EIA report OR developed periodically environmental measures for dust, GHG emissions, and noise? 🖵 Yes 🖵 No | |
| FE1.2 | | | Has the project team utilized products with a reduced carbon footprint? 🖵 Yes 🖵 No | |
| **FE5** | | | **Examining and reducing potential air and water pollution from the project and its impact on the local climate** | |
| FE5.1 | | | To what extent has the project contributed to reducing the potential air pollution resulted from the construction/operation stage? | |
|  | | | 🖵 0 🖵 < 25% 🖵 25:50% 🖵 50:75% 🖵 > 75% 🖵 100% | |
| FE5.2 | | | To what extent has the project contributed to reducing the water pollution resulted from the construction/operation stage? | |
|  | | | 🖵 0 🖵 < 25% 🖵 25:50% 🖵 50:75% 🖵 > 75% 🖵 100% | |
| FE5.3 | | | Has the project considered using equipment and vehicles that replace diesel engines with other clean technology to minimize the impact on the local climate? 🖵 Yes 🖵 No | |
| **FE15** | | | **Saving energy and resources consumption through the project life cycle** | |
| FE15.1 | | | Has the project considered using treated/recycled water (after sampling and analyzing) during the construction, maintenance, and operations stages? 🖵 Yes 🖵 No | |
| FE15.2 | | | To what extent has the project been designed to reduce energy consumption? | |
|  | | | 🖵 Using solar battery/biogas powered street lighting 🖵 Using LED street lighting | |
|  | | | 🖵 Replacing the traffic signs with retro-reflective sign panels | |
|  | | | 🖵 Implementing solar bus stops And/Or providing buses powered by clean energy (i.e., green hydrogen) | |
|  | | | 🖵 Using asphalt And/Or cement been produced using energy and fuel-saving technologies | |
|  | | | 🖵 Using WMA/CMA | |
| **FE33** | | | **Use recycled materials to reduce wastes** | |
| FE33.1 | | | To what extent has the design considered the optimum use of recycled material to reduce wastes and energy consumption? 🖵 0 🖵 < 5% 🖵 5:15% 🖵 15:25% 🖵 25:50% 🖵 > 50% | |
|  | | |  | |
| FE33.2 | | | Has the recycling process been carried out in-situ OR transported by a certified contractor to reduce the environmental impacts? 🖵 Yes 🖵 No | |
| FE33.3 | | | Has the project considered the use of locally produced by-products to be reused to reduce the resulting wastes? 🖵 Yes 🖵 No | |
| FE33.4 | | | To what extent has the project considered the precautions needed for the safe collection, storage, and disposal of hazardous and toxic materials?  🖵 Acceptable 🖵 Good 🖵 Very good 🖵 Excellent | |
| FE33.5 | | | Has a comprehensive waste management plan been developed to decrease project wastes and divert wastes from landfills during construction? 🖵 Yes 🖵 No | |
| **FE39** | | | **Provide and increase access to safe, green and public spaces for all, and confront desertification, in addition to restore degraded land and soil** | |
| FE39.1 | | | To what extent has the project increased the landscape area?  🖵 0 🖵 < 5% 🖵 5:15% 🖵 15:30% 🖵 > 30% | |
| FE39.2 | | | Has the project created physically accessible open space that encourages interaction with the environment, social interaction, and physical activities? 🖵 Yes 🖵 No | |
| FE39.3 | | | Have the project specifications maximized the planting (e.g., landscape or trees) on horizontal surfaces and slopes where feasible? 🖵 Yes 🖵 No | |
| FE39.4 | | | Has the project preserved the visual quality of the project area during construction/maintenance stages (i.e., natural features, signs, views, transit stations, street furniture, new/changed lighting, and free-standing sculptures)? 🖵 Yes 🖵 No | |
| FE39.5 | | | Has the project preserved/restored the quality of landscapes and structures that were disturbed by the project during the construction and maintenance stages? 🖵 Yes 🖵 No | |
| **Social Factors** | | |  |  |
| **FS2** | **Improve local infrastructure capacity (roads) and ensure proper services and infrastructure for all** | |  |  |
| FS2.1 | Has the project included new connections between communities? 🖵 Yes 🖵 No | |  |  |
| FS2.2 | Has the project negatively impacted any communities during the construction/maintenance stages? 🖵 Yes 🖵 No | |  |  |
| FS2.3 | To what extent has the project considered strategies to increase capacity, manage congestion, reduce vehicle distance traveled, or lower accident rates? | |  |  |
|  | 🖵 Acceptable 🖵 Good 🖵 Very good 🖵 Excellent | |  |  |
| FS2.4 | Has the project team obtained data from the community and key stakeholders regarding issues of mobility and access to ensure the feasibility of the project implementation and its compatibility with the growth rate? 🖵 Yes 🖵 No | |  |  |
| FS2.5 | Has the project considered permeable techniques that cope with flood prevention? 🖵 Yes 🖵 No | |  |  |
| **FS3** | **Select a suitable/undeveloped site for the project, which is utilized effectively** | |  |  |
| FS3.1 | To what extent has the project been located on previously developed land? | |  |  |
|  | 🖵 0 🖵 < 25% 🖵 25:50% 🖵 50:75% 🖵 more than 75% 🖵 100% | |  |  |
| FS3.2 | To what extent does the project protect or preserve high-ecological value lands and farmland? | |  |  |
|  | 🖵 < 15% 🖵 < 10% 🖵 5% 🖵 0% | |  |  |
| FS3.3 | Has the project provided an effective protective buffer zone around areas of high ecological value?  🖵 Yes 🖵 No | |  |  |
| FS3.4 | Has the project team adjusted the alignments to avoid/minimize impacts on social/environmental resources (i.e., avoidance of parklands, wetlands, historic sites, residential and commercial buildings, etc.)? 🖵 Yes 🖵 No | |  |  |
| **FS37** | **Provide access to safe and sustainable transportation systems for all, including enhancement of road and drivers’ safety, and encourage carpooling and bicycle** | |  |  |
| FS37.1 | Does the project encourage the use of public transit (e.g., bus shelters, Park-and-Ride) to decrease traffic congestion? 🖵 Yes 🖵 No | |  |  |
| FS37.2 | To what extent has the project provided addressed and documented access, safety, and wayfinding for accident management? | |  |  |
|  | 🖵 Emergency exits 🖵 Emergency lane 🖵 Ambulance unit 🖵 Rescue police | |  |  |
| FS37.3 | To what extent has the project considered the cyclist amenities, safety, and comfort? | |  |  |
|  | 🖵 Resurfaced and separated lanes 🖵 Bicycle signals 🖵 Bike route wayfinding | |  |  |
|  | 🖵 Pavement marking and colored pavement 🖵 Bicycle racks | |  |  |
| FS37.4 | To what extent has the project considered the pedestrian amenities, safety, and comfort? | |  |  |
|  | 🖵 Increasing the width of sidewalks 🖵 Providing sufficient lighting on all sidewalks and crosswalks | |  |  |
|  | 🖵 Improving the design of intersections and midblock crossings for pedestrians (such as countdown signals, pedestrian intervals, raised crosswalks | |  |  |
|  | 🖵 Bus stops | |  |  |
| FS37.5 | To what extent has the project been designated to include Park-and-Ride lots? | |  |  |
|  | 🖵 Not existed 🖵 Existed only 🖵 Existed with green shading | |  |  |
| FS37.6 | Has the project included bike accommodation at Park-and-Ride lots & transit stations? 🖵 Yes 🖵 No | |  |  |
| FS37.7 | Has the project included stop amenities for users and vehicles to increase the users’ comfort? 🖵 Yes 🖵 No | |  |  |
| FS37.8 | Has the project planted trees to provide a physical buffer between pedestrians and moving vehicles, and reduce traffic speeds? 🖵 Yes 🖵 No | |  |  |
| FS37.9 | Has the project aligned the roadway to facilitate the development of the future project extension?  🖵 Yes 🖵 No | |  |  |

**Table S16** The data collected from case study (1) for the questions of the HWS RS

- This table shows the data collected from case study (1) and the answers for each question included in each factor. Moreover, the black-colored questions refer to that this question is not applicable for a certain stage Or a roadway type.

| **Economic Factors** | | |
| --- | --- | --- |
| **FC5** | **Develop a feasibility study to define the capital budget needed for the project** | |
| FC5.1 | Has the project team provided a feasibility study including financial, technical, marketing, environmental, and economic data? 🖵 Yes 🖵 No | |
| FC5.2 | Has the owner defined the investment sources and payments available for the project?  🖵 Yes 🖵 No | |
| FC5.3 | Was a risk assessment incorporated for revenue forecasting? 🖵 Yes 🖵 No | |
| FC5.4 | Has the project team estimated the annual budget needed for the maintenance and rehabilitation processes? 🖵 Yes 🖵 No | |
| FC5.5 | Has the owner prioritized the suggested projects according to their importance of implementation?  🖵 Yes 🖵 No | |
| **FC8** | **Selecting economic, durable, and available materials** | |
| FC8.1 | Has the project team considered using more durable alternative materials? 🖵 Yes 🖵 No | |
| FC8.2 | Has the owner been informed in case of material shortage to confirm another suitable alternative?  🖵 Yes 🖵 No | |
| FC8.3 | Has the contractor succeeded in overcoming the continuous rise of material prices, if any?  🖵 Yes 🖵 No | |
| FC8.4 | Were the expected transportation distances to the site determined to be the minimum for the majority of materials? 🖵 Yes 🖵 No | |
| **FC14** | **Reduce the consumption of various types of energy, especially fossil fuel, and encourage using renewable energy and define their costs** | |
| FC14.1 | To what extent has the project considered strategies to reduce the energy use? | |
|  | 🖵 Using High-efficiency traffic signals and luminaries (such as LED) | |
|  | 🖵 Using automatic cut-off fixtures for lighting poles | |
|  | 🖵 Replacing the lighting signals with reflective signs | |
|  | 🖵 Using recycled materials during construction/maintenance (i.e., FDR) | |
| FC14.2 | To what extent has the contractor reduced the needed energy and lighting required for the construction site? | |
|  | 🖵 0 🖵 < 5% 🖵 5:15% 🖵 15:25% 🖵 25:50% 🖵 > 50% | |
| FC14.3 | To what extent has the project met energy needs from renewable resources? | |
|  | 🖵 0 🖵 < 25% 🖵 25:50% 🖵 50:75% 🖵 > 75% 🖵 100% | |
| **FC32** | **Develop a clear and detailed program for quality management including developing quality control plan (QCP)** | |
| FC32.1 | To what extent has the contractor made construction mistakes that needed to be reworked? | |
|  | 🖵 0 🖵 < 5% 🖵 5:15% 🖵 15:30% 🖵 30:50% 🖵 > 50% | |
| FC32.2 | To what extent have the specifications been written clearly? | |
|  | 🖵 Acceptable 🖵 Good 🖵 Very good 🖵 Excellent | |
| FC32.3 | To what extent has the quality control team been qualified? | |
|  | 🖵 Acceptable 🖵 Good 🖵 Very good 🖵 Excellent | |
| FC32.4 | Has the contractor presented a corrective action plan, if needed? 🖵 Yes 🖵 No | |
| **FC42** | **Design pavement according to the regional conditions according to the soil type and traffic volume** | |
| FC42.1 | To what extent has the contractor committed to the prepared soil boring report and the approved traffic studies? | |
|  | 🖵 Acceptable 🖵 Good 🖵 Very good 🖵 Excellent | |
| FC42.2 | Has the contractor prepared another soil boring report to verify the owner’s one? 🖵 Yes 🖵 No | |
| FC42.3 | To what extent has the project considered the nearby constraints during the planning, design, and construction stages?  🖵 Acceptable 🖵 Good 🖵 Very good 🖵 Excellent | |
| FC42.4 | To what extent has the project considered the runoff discharge? | |
|  | 🖵 Acceptable 🖵 Good 🖵 Very good 🖵 Excellent | |
| **FC63** | **Design long-life pavement** | |
| FC63.1 | To what extent has the project ensured the reliability of the developed traffic data? | |
|  | 🖵 Acceptable 🖵 Good 🖵 Very good 🖵 Excellent | |
| FC63.2 | To what extent has the contractor committed to the approved designs and specifications? | |
|  | 🖵 Acceptable 🖵 Good 🖵 Very good 🖵 Excellent | |
| **FC70** | **Create, modify, and use specifications that allow for sustainability best practices and achieve the efficient use** | |
| FC70.1 | Have the specifications provided the data resources and methods that explicitly address sustainability principles and allow sustainability best practices? 🖵 Yes 🖵 No | |
| **FC71** | **Plan for long-term monitoring and maintenance** | |
| FC71.1 | Is there a clear and comprehensive plan in place for long-term monitoring and maintenance of the completed project? 🖵 Yes 🖵 No | |
| FC71.2 | Have sufficient resources and staff been assigned for long-term monitoring and maintenance of the completed project? 🖵 Yes 🖵 No | |
| FC71.3 | To what extent has the monitoring and maintenance plan been considered with operations and maintenance staff?  **(The monitoring and maintenance plan was not prepared for this project)** | |
| FC71.4 | Is there a plan in-place to re-evaluate and modify the maintenance plan based on the monitored data? 🖵 Yes 🖵 No | |
| **FC84** | **Develop life‐Cycle Cost Analyses (LCCA) and Benefit‐Cost Analysis (BCA) to apply the best alternatives** | |
| FC84.1 | Has the project team developed a life-cycle cost analysis to predict the project costs and perform short- and long-term budget forecasting? 🖵 Yes 🖵 No | |
| FC84.2 | Has the project team applied Value Engineering to compare alternatives for at least one major project component? 🖵 Yes 🖵 No | |
| **FC89** | **Accommodate multi-modal transportation uses (freight vehicles, pedestrians, ridesharing and bicyclists), including providing new intermodal connections** | |
| FC89.1 | Has the project provided a multi-modal transportation system? 🖵 Yes 🖵 No | |
| FC89.2 | To what extent has the project considered measures to reduce accidents? | |
|  | 🖵 Providing warning and traffic signals 🖵 Providing calming lanes | |
|  | 🖵 Providing buffers between large vehicles and light vehicle traffic 🖵 Providing shoulders | |
| FC89.3 | Has the project been designed for high-occupancy/carpool and articulated vehicles? 🖵 Yes 🖵 No | |
| FC89.4 | Has the project promoted a reduction in vehicle trips by encouraging increased use of public transit and ride-sharing to reduce the operational expenses? 🖵 Yes 🖵 No | |
| **Environmental Factors** | | |
| **FE1** | **Apply life cycle assessment** | |
| FE1.1 | Has the contractor submitted a certified LCA/EIA report OR developed periodically environmental measures for dust, GHG emissions, and noise? 🖵 Yes 🖵 No | |
| FE1.2 | Has the project team utilized products with a reduced carbon footprint? 🖵 Yes 🖵 No | |
| **FE5** | **Examining and reducing potential air and water pollution from the project and its impact on the local climate** | |
| FE5.1 | To what extent has the project contributed to reducing the potential air pollution resulted from the construction/operation stage? | |
|  | 🖵 0 🖵 < 25% 🖵 25:50% 🖵 50:75% 🖵 > 75% 🖵 100% | |
| FE5.2 | To what extent has the project contributed to reducing the water pollution resulted from the construction/operation stage? | |
|  | 🖵 0 🖵 < 25% 🖵 25:50% 🖵 50:75% 🖵 > 75% 🖵 100% | |
| FE5.3 | Has the project considered using equipment and vehicles that replace diesel engines with other clean technology to minimize the impact on the local climate? 🖵 Yes 🖵 No | |
| **FE15** | **Saving energy and resources consumption through the project life cycle** | |
| FE15.1 | Has the project considered using treated/recycled water (after sampling and analyzing) during the construction, maintenance, and operations stages? 🖵 Yes 🖵 No | |
| FE15.2 | To what extent has the project been designed to reduce energy consumption? | |
|  | 🖵 Using solar battery/biogas powered street lighting 🖵 Using LED street lighting | |
|  | 🖵 Replacing the traffic signs with retro-reflective sign panels | |
|  | 🖵 Implementing solar bus stops And/Or providing buses powered by clean energy (i.e., green hydrogen) | |
|  | 🖵 Using asphalt And/Or cement been produced using energy and fuel-saving technologies | |
|  | 🖵 Using WMA/CMA | |
| **FE33** | **Use recycled materials to reduce wastes** | |
| FE33.1 | To what extent has the design considered the optimum use of recycled material to reduce wastes and energy consumption? | |
|  | 🖵 0 🖵 < 5% 🖵 5:15% 🖵 15:25% 🖵 25:50% 🖵 > 50% | |
| FE33.2 | Has the recycling process been carried out in-situ OR transported by a certified contractor to reduce the environmental impacts? 🖵 Yes 🖵 No | |
| FE33.3 | Has the project considered the use of locally produced by-products to be reused to reduce the resulting wastes? 🖵 Yes 🖵 No | |
| FE33.4 | To what extent has the project considered the precautions needed for the safe collection, storage, and disposal of hazardous and toxic materials? | |
|  | 🖵 Acceptable 🖵 Good 🖵 Very good 🖵 Excellent | |
| FE33.5 | Has a comprehensive waste management plan been developed to decrease project wastes and divert wastes from landfills during construction? 🖵 Yes 🖵 No | |
| **FE39** | **Provide and increase access to safe, green and public spaces for all, and confront desertification, in addition to restore degraded land and soil** | |
| FE39.1 | To what extent has the project increased the landscape area? | |
|  | 🖵 0 🖵 < 5% 🖵 5:15% 🖵 15:30% 🖵 > 30% | |
| FE39.2 | Has the project created physically accessible open space that encourages interaction with the environment, social interaction, and physical activities? 🖵 Yes 🖵 No | |
| FE39.3 | Have the project specifications maximized the planting (e.g., landscape or trees) on horizontal surfaces and slopes where feasible? 🖵 Yes 🖵 No | |
| FE39.4 | Has the project preserved the visual quality of the project area during construction/maintenance stages (i.e., natural features, signs, views, transit stations, street furniture, new/changed lighting, and free-standing sculptures)? 🖵 Yes 🖵 No | |
| FE39.5 | Has the project preserved/restored the quality of landscapes and structures that were disturbed by the project during the construction and maintenance stages? 🖵 Yes 🖵 No | |
| **Social Factors** | |  |
| **FS2** | **Improve local infrastructure capacity (roads) and ensure proper services and infrastructure for all** |  |
| FS2.1 | Has the project included new connections between communities?  **Not applicable for the maintenance stage** |  |
| FS2.2 | Has the project negatively impacted any communities during the construction/maintenance stages? 🖵 Yes 🖵 No |  |
| FS2.3 | To what extent has the project considered strategies to increase capacity, manage congestion, reduce vehicle distance traveled, or lower accident rates? |  |
|  | 🖵 Acceptable 🖵 Good 🖵 Very good 🖵 Excellent |  |
| FS2.4 | Has the project team obtained data from the community and key stakeholders regarding issues of mobility and access to ensure the feasibility of the project implementation and its compatibility with the growth rate? 🖵 Yes 🖵 No |  |
| FS2.5 | Has the project considered permeable techniques that cope with flood prevention? 🖵 Yes 🖵 No |  |
| **FS3** | **Select a suitable/undeveloped site for the project, which is utilized effectively** |  |
| FS3.1 | To what extent has the project been located on previously developed land? |  |
|  | 🖵 0 🖵 < 25% 🖵 25:50% 🖵 50:75% 🖵 more than 75% 🖵 100% |  |
| FS3.2 | To what extent does the project protect or preserve high-ecological value lands and farmland? |  |
|  | 🖵 < 15% 🖵 < 10% 🖵 5% 🖵 0% |  |
| FS3.3 | Has the project provided an effective protective buffer zone around areas of high ecological value?  **Not applicable for the maintenance stage** |  |
| FS3.4 | Has the project team adjusted the alignments to avoid/minimize impacts on social/environmental resources (i.e., avoidance of parklands, wetlands, historic sites, residential and commercial buildings, etc.)? **Not applicable for the maintenance stage** |  |
| **FS37** | **Provide access to safe and sustainable transportation systems for all, including enhancement of road and drivers’ safety, and encourage carpooling and bicycle** |  |
| FS37.1 | Does the project encourage the use of public transit (e.g., bus shelters, Park-and-Ride) to decrease traffic congestion? 🖵 Yes 🖵 No |  |
| FS37.2 | To what extent has the project provided addressed and documented access, safety, and wayfinding for accident management? |  |
|  | 🖵 Emergency exits 🖵 Emergency lane 🖵 Ambulance unit 🖵 Rescue police |  |
| FS37.3 | To what extent has the project considered the cyclist amenities, safety, and comfort? **Not applicable for the maintenance stage** |  |
| FS37.4 | To what extent has the project considered the pedestrian amenities, safety, and comfort?  **Not applicable for the maintenance stage** |  |
| FS37.5 | To what extent has the project been designated to include Park-and-Ride lots? |  |
|  | 🖵 Not existed 🖵 Existed only 🖵 Existed with green shading |  |
| FS37.6 | Has the project included bike accommodation at Park-and-Ride lots & transit stations?  **Not applicable for the maintenance stage** |  |
| FS37.7 | Has the project included stop amenities for users and vehicles to increase the users’ comfort? 🖵 Yes 🖵 No |  |
| FS37.8 | Has the project planted trees to provide a physical buffer between pedestrians and moving vehicles, and reduce traffic speeds?  **Not applicable for the maintenance stage** |  |
| FS37.9 | Has the project aligned the roadway to facilitate the development of the future project extension?  🖵 Yes 🖵 No |  |

**Table S17.** The sustainability performance for case study (1) through Envision RS

| **Summary Results** | | |  |  |  |  |  |
| --- | --- | --- | --- | --- | --- | --- | --- |
|  |  |  |  |  |  |  |  |
|  |  |  | **Credit Assessment Status** | **Evaluation Questions Assessed** | | **Assessed Maximum Points Available** | **Total Maximum Points** |
|  |  |  |  | **Yes** | **No** |  |  |
|  | **Wellbeing** | QL1.1 Improve Community Quality of Life | Assessed | 4 | 3 | 26 | 26 |
|  |  | QL1.2 Enhance Public Health & Safety | Assessed | 3 | 3 | 20 | 20 |
|  |  | QL1.3 Improve Construction Safety | Assessed | 2 | 3 | 14 | 14 |
|  |  | QL1.4 Minimize Noise & Vibration | Assessed | 1 | 4 | 12 | 12 |
| 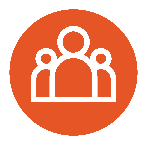   \|  \| \| --- \| |  | QL1.5 Minimize Light Pollution | Not Applicable | 0 | 0 | 0 | 12 |
|  |  | QL1.6 Minimize Construction Impacts | Not Applicable | 0 | 0 | 0 | 8 |
|  | **Mobility** | QL2.1 Improve Community Mobility Access | Assessed | 5 | 1 | 14 | 14 |
| **Quality of Life** |  | QL2.2 Encourage Sustainable Transportation | Assessed | 3 | 1 | 16 | 16 |
|  |  | QL2.3 Improve Access & Wayfinding | Assessed | 2 | 2 | 14 | 14 |
|  | **Community** | QL3.1 Advance Equity & Social Justice | Assessed | 5 | 2 | 18 | 18 |
|  |  | QL3.2 Preserve Historic & Cultural Resources | Not Applicable | 0 | 0 | 0 | 18 |
|  |  | QL3.3 Enhance Views & Local Character | Assessed | 5 | 1 | 14 | 14 |
|  |  | QL3.4 Enhance Public Space & Amenities | Not Applicable | 0 | 0 | 0 | 14 |
|  |  |  |  |  |  |  |  |
|  |  |  | **Credit Assessment Status** | **Evaluation Questions Assessed** | | **Assessed Maximum Points Available** | **Total Maximum Points** |
|  |  |  |  | **Yes** | **No** |  |  |
|  | **Collaboration** | LD1.1 Provide Effective Leadership & Commitment | Not Applicable | 0 | 0 | 0 | 18 |
| 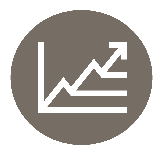 |  | LD1.2 Foster Collaboration & Teamwork | Not Applicable | 0 | 0 | 0 | 18 |
| \|  \| \| --- \| |  | LD1.3 Provide for Stakeholder Involvement | Assessed | 0 | 6 | 18 | 18 |
|  |  | LD1.4 Pursue Byproduct Synergies | Assessed | 5 | 0 | 18 | 18 |
|  | **Planning** | LD2.1 Establish a Sustainability Management Plan | Not Applicable | 0 | 0 | 0 | 18 |
|  |  | LD2.2 Plan for Sustainable Communities | Not Applicable | 0 | 0 | 0 | 16 |
| **Leadership** |  | LD2.3 Plan for Long-Term Monitoring & Maintenance | Not Applicable | 0 | 0 | 0 | 12 |
|  |  | LD2.4 Plan for End-of-Life | Not Applicable | 0 | 0 | 0 | 14 |
|  | **Economy** | LD3.1 Stimulate Economic Prosperity & Development | Assessed | 5 | 0 | 20 | 20 |
|  |  | LD3.2 Develop Local Skills & Capabilities | Assessed | 2 | 2 | 16 | 16 |
|  |  | LD3.3 Conduct a Life-Cycle Economic Evaluation | Not Applicable | 0 | 0 | 0 | 14 |
|  |  |  |  |  |  |  |  |
|  |  |  | **Credit Assessment Status** | **Evaluation Questions Assessed** | | **Assessed Maximum Points Available** | **Total Maximum Points** |
|  |  |  |  | **Yes** | **No** |  |  |
|  | **Materials** | RA1.1 Support Sustainable Procurement Practices | Assessed | 2 | 0 | 12 | 12 |
|  |  | RA1.2 Use Recycled Materials | Assessed | 1 | 0 | 16 | 16 |
|  |  | RA1.3 Reduce Operational Waste | Assessed | 1 | 1 | 14 | 14 |
|  |  | RA1.4 Reduce Construction Waste | Assessed | 2 | 0 | 16 | 16 |
| 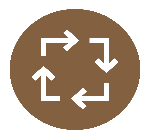   \|  \| \| --- \| |  | RA1.5 Balance Earthwork On Site | Assessed | 1 | 0 | 8 | 8 |
|  | **Energy** | RA2.1 Reduce Operational Energy Consumption | Not Applicable | 0 | 0 | 0 | 26 |
|  |  | RA2.2 Reduce Construction Energy Consumption | Not Applicable | 0 | 0 | 0 | 12 |
| **Resource** |  | RA2.3 Use Renewable Energy | Not Applicable | 0 | 0 | 0 | 24 |
| **Allocation** |  | RA2.4 Commission & Monitor Energy Systems | Not Applicable | 0 | 0 | 0 | 14 |
|  | **Water** | RA3.1 Preserve Water Resources | Assessed | 0 | 6 | 12 | 12 |
|  |  | RA3.2 Reduce Operational Water Consumption | Not Applicable | 0 | 0 | 0 | 22 |
|  |  | RA3.3 Reduce Construction Water Consumption | Assessed | 0 | 2 | 8 | 8 |
|  |  | RA3.4 Monitor Water Systems | Assessed | 0 | 2 | 12 | 12 |
|  |  |  |  |  |  |  |  |
|  |  |  | **Credit Assessment Status** | **Evaluation Questions Assessed** | | **Assessed Maximum Points Available** | **Total Maximum Points** |
|  |  |  |  | **Yes** | **No** |  |  |
|  | **Siting** | NW1.1 Preserve Sites of High Ecological Value | Assessed | 4 | 2 | 22 | 22 |
|  |  | NW1.2 Provide Wetland & Surface Water Buffers | Not Applicable | 0 | 0 | 0 | 20 |
|  |  | NW1.3 Preserve Prime Farmland | Assessed | 5 | 0 | 16 | 16 |
|  |  | NW1.4 Preserve Undeveloped Land | Assessed | 1 | 1 | 24 | 24 |
| 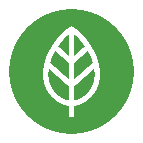   \|  \| \| --- \| | **Conservation** | NW2.1 Reclaim Brownfields | Not Applicable | 0 | 0 | 0 | 22 |
|  |  | NW2.2 Manage Stormwater | Assessed | 1 | 3 | 24 | 24 |
|  |  | NW2.3 Reduce Pesticide & Fertilizer Impacts | Not Assessed | 0 | 0 | 12 | 12 |
| **Natural** |  | NW2.4 Protect Surface & Groundwater Quality | Not Applicable | 0 | 0 | 0 | 20 |
| **World** | **Ecology** | NW3.1 Enhance Functional Habitats | Not Applicable | 0 | 0 | 0 | 18 |
|  |  | NW3.2 Enhance Wetland & Surface Water Functions | Not Assessed | 0 | 0 | 20 | 20 |
|  |  | NW3.3 Maintain Floodplain Functions | Not Applicable | 0 | 0 | 0 | 14 |
|  |  | NW3.4 Control Invasive Species | Not Assessed | 0 | 0 | 12 | 12 |
|  |  | NW3.5 Protect Soil Health | Assessed | 2 | 2 | 8 | 8 |
|  |  |  |  |  |  |  |  |
|  |  |  | **Credit Assessment Status** | **Evaluation Questions Assessed** | | **Assessed Maximum Points Available** | **Total Maximum Points** |
|  |  |  |  | **Yes** | **No** |  |  |
|  | **Emissions** | CR1.1 Reduce Net Embodied Carbon | Assessed | 1 | 2 | 20 | 20 |
|  |  | CR1.2 Reduce Greenhouse Gas Emissions | Assessed | 1 | 1 | 26 | 26 |
| 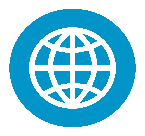   \|  \| \| --- \| |  | CR1.3 Reduce Air Pollutant Emissions | Not Applicable | 0 | 0 | 0 | 18 |
|  | **Resilience** | CR2.1 Avoid Unsuitable Development | Assessed | 3 | 3 | 16 | 16 |
|  |  | CR2.2 Assess Climate Change Vulnerability | Assessed | 3 | 2 | 20 | 20 |
| **Climate and** |  | CR2.3 Evaluate Risk and Resilience | Assessed | 6 | 0 | 26 | 26 |
| **Resilience** |  | CR2.4 Establish Resilience Goals and Strategies | Assessed | 2 | 2 | 20 | 20 |
|  |  | CR2.5 Maximize Resilience | Assessed | 3 | 2 | 26 | 26 |
|  |  | CR2.6 Improve Infrastructure Integration | Assessed | 3 | 2 | 18 | 18 |
|  |  |  |  |  |  |  |  |
|  |  |  | **Credit Assessment Status** | **Evaluation Questions Assessed** | | **Assessed Maximum Points Available** | **Total Maximum Points** |
|  |  |  |  | **Yes** | **No** |  |  |
|  |  | **Total Points** | **3 Not Assessed** | **84** | **61** | **628** | **1000** |
|  |  |  |  |  |  |  |  |
|  |  | **Possible Award Level:** | **Silver** | | | |  |

**Table S18** The data collected from case study (2) for the questions of the HWS RS

- This table shows the data collected from case study (2) and the answers for each question included in each factor. Moreover, the black-colored questions refer to that this question is not applicable for a certain stage or roadway type.

| **Economic Factors** | |  |
| --- | --- | --- |
| **FC5** | **Develop a feasibility study to define the capital budget needed for the project** |  |
| FC5.1 | Has the project team provided a feasibility study including financial, technical, marketing, environmental, and economic data? 🖵 Yes 🖵 No |  |
| FC5.2 | Has the owner defined the investment sources and payments available for the project?  🖵 Yes 🖵 No |  |
| FC5.3 | Was a risk assessment incorporated for revenue forecasting? 🖵 Yes 🖵 No |  |
| FC5.4 | Has the project team estimated the annual budget needed for the maintenance and rehabilitation processes? **Not applicable for the construction stage** |  |
| FC5.5 | Has the owner prioritized the suggested projects according to their importance of implementation? 🖵 Yes 🖵 No |  |
| **FC8** | **Selecting economic, durable, and available materials** |  |
| FC8.1 | Has the project team considered using more durable alternative materials?  🖵 Yes 🖵 No |  |
| FC8.2 | Has the owner been informed in case of material shortage to confirm another suitable alternative?  🖵 Yes 🖵 No |  |
| FC8.3 | Has the contractor succeeded in overcoming the continuous rise of material prices, if any?  🖵 Yes 🖵 No |  |
| FC8.4 | Were the expected transportation distances to the site determined to be the minimum for the majority of materials? 🖵 Yes 🖵 No |  |
| **FC14** | **Reduce the consumption of various types of energy, especially fossil fuel, and encourage using renewable energy and define their costs** |  |
| FC14.1 | To what extent has the project considered strategies to reduce the energy use? |  |
|  | 🖵 Using High-efficiency traffic signals and luminaries (such as LED) |  |
|  | 🖵 Using automatic cut-off fixtures for lighting poles |  |
|  | 🖵 Replacing the lighting signals with reflective signs |  |
|  | 🖵 Using recycled materials during construction/maintenance (i.e., FDR) |  |
| FC14.2 | To what extent has the contractor reduced the needed energy and lighting required for the construction site? |  |
|  | 🖵 0 🖵 < 5% 🖵 5:15% 🖵 15:25% 🖵 25:50% 🖵 > 50% |  |
| FC14.3 | To what extent has the project met energy needs from renewable resources? |  |
|  | 🖵 0 🖵 < 25% 🖵 25:50% 🖵 50:75% 🖵 > 75% 🖵 100% |  |
| **FC32** | **Develop a clear and detailed program for quality management including developing quality control plan (QCP)** |  |
| FC32.1 | To what extent has the contractor made construction mistakes that needed to be reworked? |  |
|  | 🖵 0 🖵 < 5% 🖵 5:15% 🖵 15:30% 🖵 30:50% 🖵 > 50% |  |
| FC32.2 | To what extent have the specifications been written clearly? |  |
|  | 🖵 Acceptable 🖵 Good 🖵 Very good 🖵 Excellent |  |
| FC32.3 | To what extent has the quality control team been qualified? |  |
|  | 🖵 Acceptable 🖵 Good 🖵 Very good 🖵 Excellent |  |
| FC32.4 | Has the contractor presented a corrective action plan, if needed? 🖵 Yes 🖵 No |  |
| **FC42** | **Design pavement according to the regional conditions according to the soil type and traffic volume** |  |
| FC42.1 | To what extent has the contractor committed to the prepared soil boring report and the approved traffic studies? |  |
|  | 🖵 Acceptable 🖵 Good 🖵 Very good 🖵 Excellent |  |
| FC42.2 | Has the contractor prepared another soil boring report to verify the owner’s one?  🖵 Yes 🖵 No |  |
| FC42.3 | To what extent has the project considered the nearby constraints during the planning, design, and construction stages?  🖵 Acceptable 🖵 Good 🖵 Very good 🖵 Excellent |  |
| FC42.4 | To what extent has the project considered the runoff discharge? |  |
|  | 🖵 Acceptable 🖵 Good 🖵 Very good 🖵 Excellent |  |
| **FC63** | **Design long-life pavement** |  |
| FC63.1 | To what extent has the project ensured the reliability of the developed traffic data? |  |
|  | 🖵 Acceptable 🖵 Good 🖵 Very good 🖵 Excellent |  |
| FC63.2 | To what extent has the contractor committed to the approved designs and specifications? |  |
|  | 🖵 Acceptable 🖵 Good 🖵 Very good 🖵 Excellent |  |
| **FC70** | **Create, modify, and use specifications that allow for sustainability best practices and achieve the efficient use** |  |
| FC70.1 | Have the specifications provided the data resources and methods that explicitly address sustainability principles and allow sustainability best practices?  🖵 Yes 🖵 No |  |
| **FC71** | **Plan for long-term monitoring and maintenance** |  |
| FC71.1 | Is there a clear and comprehensive plan in place for long-term monitoring and maintenance of the completed project? 🖵 Yes 🖵 No |  |
| FC71.2 | Have sufficient resources and staff been assigned for long-term monitoring and maintenance of the completed project? 🖵 Yes 🖵 No |  |
| FC71.3 | To what extent has the monitoring and maintenance plan been considered with operations and maintenance staff? **Not applicable for the construction stage** |  |
| FC71.4 | Is there a plan in-place to re-evaluate and modify the maintenance plan based on the monitored data? **Not applicable for the construction stage** |  |
| **FC84** | **Develop life‐Cycle Cost Analyses (LCCA) and Benefit‐Cost Analysis (BCA) to apply the best alternatives** |  |
| FC84.1 | Has the project team developed a life-cycle cost analysis to predict the project costs and perform short- and long-term budget forecasting? 🖵 Yes 🖵 No |  |
| FC84.2 | Has the project team applied Value Engineering to compare alternatives for at least one major project component? 🖵 Yes 🖵 No |  |
| **FC89** | **Accommodate multi-modal transportation uses (freight vehicles, pedestrians, ridesharing and bicyclists), including providing new intermodal connections** |  |
| FC89.1 | Has the project provided a multi-modal transportation system? 🖵 Yes 🖵 No |  |
| FC89.2 | To what extent has the project considered measures to reduce accidents? |  |
|  | 🖵 Providing warning and traffic signals  🖵 Providing calming lanes |  |
|  | 🖵 Providing buffers between large vehicles and light vehicle traffic  🖵 Providing shoulders |  |
| FC89.3 | Has the project been designed for high-occupancy/carpool and articulated vehicles? 🖵 Yes 🖵 No |  |
| FC89.4 | Has the project promoted a reduction in vehicle trips by encouraging increased use of public transit and ride-sharing to reduce the operational expenses?  🖵 Yes 🖵 No |  |
| **Environmental Factors** | |  |
| **FE1** | **Apply life cycle assessment** |  |
| FE1.1 | Has the contractor submitted a certified LCA/EIA report OR developed periodically environmental measures for dust, GHG emissions, and noise? 🖵 Yes 🖵 No |  |
| FE1.2 | Has the project team utilized products with a reduced carbon footprint? 🖵 Yes 🖵 No |  |
| **FE5** | **Examining and reducing potential air and water pollution from the project and its impact on the local climate** |  |
| FE5.1 | To what extent has the project contributed to reducing the potential air pollution resulted from the construction/operation stage? |  |
|  | 🖵 0 🖵 < 25% 🖵 25:50% 🖵 50:75% 🖵 > 75% 🖵 100% |  |
| FE5.2 | To what extent has the project contributed to reducing the water pollution resulted from the construction/operation stage? |  |
|  | 🖵 0 🖵 < 25% 🖵 25:50% 🖵 50:75% 🖵 > 75% 🖵 100% |  |
| FE5.3 | Has the project considered using equipment and vehicles that replace diesel engines with other clean technology to minimize the impact on the local climate? 🖵 Yes 🖵 No |  |
| **FE15** | **Saving energy and resources consumption through the project life cycle** |  |
| FE15.1 | Has the project considered using treated/recycled water (after sampling and analyzing) during the construction, maintenance, and operations stages? 🖵 Yes 🖵 No |  |
| FE15.2 | To what extent has the project been designed to reduce energy consumption? |  |
|  | 🖵 Using solar battery/biogas powered street lighting  🖵 Using LED street lighting |  |
|  | 🖵 Replacing the traffic signs with retro-reflective sign panels  🖵 Implementing solar bus stops And/Or providing buses powered by clean energy (i.e., green hydrogen) |  |
|  | 🖵 Using asphalt And/Or cement been produced using energy and fuel-saving technologies |  |
|  | 🖵 Using WMA/CMA |  |
| **FE33** | **Use recycled materials to reduce wastes** |  |
| FE33.1 | To what extent has the design considered the optimum use of recycled material to reduce wastes and energy consumption? |  |
|  | 🖵 0 🖵 < 5% 🖵 5:15% 🖵 15:25% 🖵 25:50% 🖵 > 50% |  |
| FE33.2 | Has the recycling process been carried out in-situ OR transported by a certified contractor to reduce the environmental impacts? 🖵 Yes 🖵 No |  |
| FE33.3 | Has the project considered the use of locally produced by-products to be reused to reduce the resulting wastes? 🖵 Yes 🖵 No |  |
| FE33.4 | Has the project considered the precautions needed for the safe collection, storage, and disposal of hazardous and toxic materials? 🖵 Yes 🖵 No |  |
| FE33.5 | Has a comprehensive waste management plan been developed to decrease project wastes and divert wastes from landfills during construction? 🖵 Yes 🖵 No |  |
| **FE39** | **Provide and increase access to safe, green and public spaces for all, and confront desertification, in addition to restore degraded land and soil** |  |
| FE39.1 | To what extent has the project increased the landscape area? |  |
|  | 🖵 0 🖵 < 5% 🖵 5:15% 🖵 15:30% 🖵 > 30% |  |
| FE39.2 | Has the project created physically accessible open space that encourages interaction with the environment, social interaction, and physical activities? 🖵 Yes 🖵 No |  |
| FE39.3 | Have the project specifications maximized the planting (e.g., landscape or trees) on horizontal surfaces and slopes where feasible? 🖵 Yes 🖵 No |  |
| FE39.4 | Has the project preserved the visual quality of the project area during construction/maintenance stages (i.e., natural features, signs, views, transit stations, street furniture, new/changed lighting, and free-standing sculptures)? 🖵 Yes 🖵 No |  |
| FE39.5 | Has the project preserved/restored the quality of landscapes and structures that were disturbed by the project during the construction and maintenance stages? 🖵 Yes 🖵 No |  |
| **Social Factors** | | |
| **FS2** | **Improve local infrastructure capacity (roads) and ensure proper services and infrastructure for all** | |
| FS2.1 | Has the project included new connections between communities? 🖵 Yes 🖵 No | |
| FS2.2 | Has the project negatively impacted any communities during the construction/maintenance stages? 🖵 Yes 🖵 No | |
| FS2.3 | To what extent has the project considered strategies to increase capacity, manage congestion, reduce vehicle distance traveled, or lower accident rates? | |
|  | 🖵 Acceptable 🖵 Good 🖵 Very good 🖵 Excellent | |
| FS2.4 | Has the project team obtained data from the community and key stakeholders regarding issues of mobility and access to ensure the feasibility of the project implementation and its compatibility with the growth rate? 🖵 Yes 🖵 No | |
| FS2.5 | Has the project considered permeable techniques that cope with flood prevention? 🖵 Yes 🖵 No | |
| **FS3** | **Select a suitable/undeveloped site for the project, which is utilized effectively** | |
| FS3.1 | To what extent has the project been located on previously developed land? | |
|  | 🖵 0 🖵 < 25% 🖵 25:50% 🖵 50:75% 🖵 more than 75% 🖵 100% | |
| FS3.2 | To what extent does the project protect or preserve high-ecological value lands and farmland? | |
|  | 🖵 < 15% 🖵 < 10% 🖵 5% 🖵 0% | |
| FS3.3 | Has the project provided an effective protective buffer zone around areas of high ecological value?  🖵 Yes 🖵 No | |
| FS3.4 | Has the project team adjusted the alignments to avoid/minimize impacts on social/environmental resources (i.e., avoidance of parklands, wetlands, historic sites, residential and commercial buildings, etc.)? 🖵 Yes 🖵 No | |
| **FS37** | **Provide access to safe and sustainable transportation systems for all, including enhancement of road and drivers’ safety, and encourage carpooling and bicycle** | |
| FS37.1 | Does the project encourage the use of public transit (e.g., bus shelters, Park-and-Ride) to decrease traffic congestion? 🖵 Yes 🖵 No | |
| FS37.2 | To what extent has the project provided addressed and documented access, safety, and wayfinding for accident management? | |
|  | 🖵 Emergency exits 🖵 Emergency lane  🖵 Ambulance unit 🖵 Rescue police | |
| FS37.3 | To what extent has the project considered the cyclist amenities, safety, and comfort? | |
|  | **Not applicable for the rural project** | |
| FS37.4 | To what extent has the project considered the pedestrian amenities, safety, and comfort? | |
|  | **Not applicable for the rural project** | |
| FS37.5 | To what extent has the project been designated to include Park-and-Ride lots? | |
|  | 🖵 Not existed 🖵 Existed only 🖵 Existed with green shading | |
| FS37.6 | Has the project included bike accommodation at Park-and-Ride lots & transit stations? **Not applicable for the rural project** | |
| FS37.7 | Has the project included stop amenities for users and vehicles to increase the users’ comfort?  🖵 Yes 🖵 No | |
| FS37.8 | Has the project planted trees to provide a physical buffer between pedestrians and moving vehicles, and reduce traffic speeds? **Not applicable for the rural project** | |
| FS37.9 | Has the project aligned the roadway to facilitate the development of the future project extension?  🖵 Yes 🖵 No | |

**Table S19.** The sustainability performance for case study (2) through Envision RS

| **Summary Results** | | |  |  |  |  |  |
| --- | --- | --- | --- | --- | --- | --- | --- |
|  |  |  |  |  |  |  |  |
|  |  |  | **Credit Assessment Status** | **Evaluation Questions Assessed** | | **Assessed Maximum Points Available** | **Total Maximum Points** |
|  |  |  |  | **Yes** | **No** |  |  |
|  | **Wellbeing** | QL1.1 Improve Community Quality of Life | Assessed | 6 | 1 | 26 | 26 |
|  |  | QL1.2 Enhance Public Health & Safety | Assessed | 4 | 2 | 20 | 20 |
|  |  | QL1.3 Improve Construction Safety | Assessed | 3 | 2 | 14 | 14 |
|  |  | QL1.4 Minimize Noise & Vibration | Assessed | 1 | 4 | 12 | 12 |
| 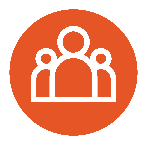   \|  \| \| --- \| |  | QL1.5 Minimize Light Pollution | Assessed | 4 | 2 | 12 | 12 |
|  |  | QL1.6 Minimize Construction Impacts | Assessed | 3 | 3 | 8 | 8 |
|  | **Mobility** | QL2.1 Improve Community Mobility Access | Assessed | 6 | 0 | 14 | 14 |
| **Quality of Life** |  | QL2.2 Encourage Sustainable Transportation | Assessed | 3 | 1 | 16 | 16 |
|  |  | QL2.3 Improve Access & Wayfinding | Assessed | 4 | 0 | 14 | 14 |
|  | **Community** | QL3.1 Advance Equity & Social Justice | Assessed | 6 | 1 | 18 | 18 |
|  |  | QL3.2 Preserve Historic & Cultural Resources | Not Applicable | 0 | 0 | 0 | 18 |
|  |  | QL3.3 Enhance Views & Local Character | Assessed | 5 | 1 | 14 | 14 |
|  |  | QL3.4 Enhance Public Space & Amenities | Assessed | 4 | 0 | 14 | 14 |
|  |  |  |  |  |  |  |  |
|  |  |  | **Credit Assessment Status** | **Evaluation Questions Assessed** | | **Assessed Maximum Points Available** | **Total Maximum Points** |
|  |  |  |  | **Yes** | **No** |  |  |
|  | **Collaboration** | LD1.1 Provide Effective Leadership & Commitment | Not Applicable | 0 | 0 | 0 | 18 |
| 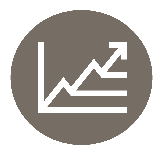 |  | LD1.2 Foster Collaboration & Teamwork | Assessed | 2 | 2 | 18 | 18 |
| \|  \| \| --- \| |  | LD1.3 Provide for Stakeholder Involvement | Assessed | 1 | 5 | 18 | 18 |
|  |  | LD1.4 Pursue Byproduct Synergies | Assessed | 2 | 3 | 18 | 18 |
|  | **Planning** | LD2.1 Establish a Sustainability Management Plan | Not Applicable | 0 | 0 | 0 | 18 |
|  |  | LD2.2 Plan for Sustainable Communities | Assessed | 5 | 0 | 16 | 16 |
| **Leadership** |  | LD2.3 Plan for Long-Term Monitoring & Maintenance | Not Applicable | 0 | 0 | 0 | 12 |
|  |  | LD2.4 Plan for End-of-Life | Not Applicable | 0 | 0 | 0 | 14 |
|  | **Economy** | LD3.1 Stimulate Economic Prosperity & Development | Assessed | 5 | 0 | 20 | 20 |
|  |  | LD3.2 Develop Local Skills & Capabilities | Assessed | 3 | 1 | 16 | 16 |
|  |  | LD3.3 Conduct a Life-Cycle Economic Evaluation | Assessed | 1 | 4 | 14 | 14 |
|  |  |  |  |  |  |  |  |
|  |  |  | **Credit Assessment Status** | **Evaluation Questions Assessed** | | **Assessed Maximum Points Available** | **Total Maximum Points** |
|  |  |  |  | **Yes** | **No** |  |  |
|  | **Materials** | RA1.1 Support Sustainable Procurement Practices | Assessed | 0 | 2 | 12 | 12 |
|  |  | RA1.2 Use Recycled Materials | Not Applicable | 0 | 0 | 0 | 16 |
|  |  | RA1.3 Reduce Operational Waste | Not Applicable | 0 | 0 | 0 | 14 |
|  |  | RA1.4 Reduce Construction Waste | Not Applicable | 0 | 0 | 0 | 16 |
| 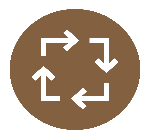   \|  \| \| --- \| |  | RA1.5 Balance Earthwork On Site | Not Applicable | 0 | 0 | 0 | 8 |
|  | **Energy** | RA2.1 Reduce Operational Energy Consumption | Assessed | 2 | 0 | 26 | 26 |
|  |  | RA2.2 Reduce Construction Energy Consumption | Not Applicable | 0 | 0 | 0 | 12 |
| **Resource** |  | RA2.3 Use Renewable Energy | Not Applicable | 0 | 0 | 0 | 24 |
| **Allocation** |  | RA2.4 Commission & Monitor Energy Systems | Not Applicable | 0 | 0 | 0 | 14 |
|  | **Water** | RA3.1 Preserve Water Resources | Not Applicable | 0 | 0 | 0 | 12 |
|  |  | RA3.2 Reduce Operational Water Consumption | Not Applicable | 0 | 0 | 0 | 22 |
|  |  | RA3.3 Reduce Construction Water Consumption | Assessed | 0 | 2 | 8 | 8 |
|  |  | RA3.4 Monitor Water Systems | Not Applicable | 0 | 0 | 0 | 12 |
|  |  |  |  |  |  |  |  |
|  |  |  | **Credit Assessment Status** | **Evaluation Questions Assessed** | | **Assessed Maximum Points Available** | **Total Maximum Points** |
|  |  |  |  | **Yes** | **No** |  |  |
|  | **Siting** | NW1.1 Preserve Sites of High Ecological Value | Assessed | 4 | 2 | 22 | 22 |
|  |  | NW1.2 Provide Wetland & Surface Water Buffers | Not Applicable | 0 | 0 | 0 | 20 |
|  |  | NW1.3 Preserve Prime Farmland | Assessed | 4 | 1 | 16 | 16 |
|  |  | NW1.4 Preserve Undeveloped Land | Assessed | 1 | 1 | 24 | 24 |
| 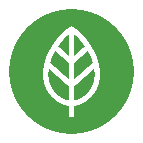   \|  \| \| --- \| | **Conservation** | NW2.1 Reclaim Brownfields | Not Applicable | 0 | 0 | 0 | 22 |
|  |  | NW2.2 Manage Stormwater | Assessed | 0 | 4 | 24 | 24 |
|  |  | NW2.3 Reduce Pesticide & Fertilizer Impacts | Not Assessed | 0 | 0 | 12 | 12 |
| **Natural** |  | NW2.4 Protect Surface & Groundwater Quality | Not Applicable | 0 | 0 | 0 | 20 |
| **World** | **Ecology** | NW3.1 Enhance Functional Habitats | Not Applicable | 0 | 0 | 0 | 18 |
|  |  | NW3.2 Enhance Wetland & Surface Water Functions | Not Assessed | 0 | 0 | 20 | 20 |
|  |  | NW3.3 Maintain Floodplain Functions | Assessed | 3 | 2 | 14 | 14 |
|  |  | NW3.4 Control Invasive Species | Not Assessed | 0 | 0 | 12 | 12 |
|  |  | NW3.5 Protect Soil Health | Assessed | 2 | 2 | 8 | 8 |
|  |  |  |  |  |  |  |  |
|  |  |  | **Credit Assessment Status** | **Evaluation Questions Assessed** | | **Assessed Maximum Points Available** | **Total Maximum Points** |
|  |  |  |  | **Yes** | **No** |  |  |
|  | **Emissions** | CR1.1 Reduce Net Embodied Carbon | Assessed | 3 | 0 | 20 | 20 |
|  |  | CR1.2 Reduce Greenhouse Gas Emissions | Assessed | 0 | 2 | 26 | 26 |
| 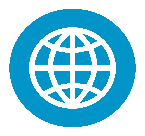   \|  \| \| --- \| |  | CR1.3 Reduce Air Pollutant Emissions | Not Applicable | 0 | 0 | 0 | 18 |
|  | **Resilience** | CR2.1 Avoid Unsuitable Development | Assessed | 5 | 1 | 16 | 16 |
|  |  | CR2.2 Assess Climate Change Vulnerability | Assessed | 3 | 2 | 20 | 20 |
| **Climate and** |  | CR2.3 Evaluate Risk and Resilience | Assessed | 5 | 1 | 26 | 26 |
| **Resilience** |  | CR2.4 Establish Resilience Goals and Strategies | Assessed | 1 | 3 | 20 | 20 |
|  |  | CR2.5 Maximize Resilience | Assessed | 1 | 4 | 26 | 26 |
|  |  | CR2.6 Improve Infrastructure Integration | Assessed | 4 | 1 | 18 | 18 |
|  |  |  |  |  |  |  |  |
|  |  |  | **Credit Assessment Status** | **Evaluation Questions Assessed** | | **Assessed Maximum Points Available** | **Total Maximum Points** |
|  |  |  |  | **Yes** | **No** |  |  |
|  |  | **Total Points** | **3 Not Assessed** | **106** | **62** | **672** | **1000** |
|  |  |  |  |  |  |  |  |
|  |  | **Possible Award Level:** | **Gold** | | | |  |

**Table S20.** The summary report developed for case study (1)

- The figure shows the summary report resulted from applying case study (1). The score for each factor is shown with a color, which indicate the status of this factor. As shown, the red color indicates that the factor needs more enhancement to achieve a more sustainability score, and the brown and green colors show that the factor meets the required sustainability score.

| **Summary Report** | | |
| --- | --- | --- |
| **Note:** The summary report presents guidelines for the factors which need to be considered/improved | | |
| **Economic Aspects** | | |
| 1 | Plan for long-term monitoring and maintenance | **0.00** |
| 2 | Develop life‐Cycle Cost Analyses (LCCA) and Benefit‐Cost Analysis (BCA) to apply the best alternatives | **0.00** |
| 3 | Reduce the consumption of various types of energy, especially fossil fuel, and encourage using renewable energy and define their costs | **0.83** |
| 4 | Develop a feasibility study to define the capital budget needed for the project | **2.00** |
| 5 | Accommodate multi-modal transportation uses (freight vehicles, pedestrians, ridesharing and bicyclists), including providing new intermodal connections | **3.23** |
| 6 | Design pavement according to the regional conditions according to the soil type and traffic volume | **4.43** |
| 7 | Approved | **5.05** |
| 8 | Approved | **5.05** |
| 9 | Approved | **5.10** |
| 10 | Approved | **10.00** |
| **Environmental Aspects** | | |
| 1 | Saving energy and resources consumption through the project life cycle | **0.93** |
| 2 | Examining and reducing potential air and water pollution from the project and its impact on the local climate | **2.02** |
| 3 | Provide and increase access to safe, green and public spaces for all, and confront desertification, in addition to restore degraded land and soil | **4.10** |
| 4 | Apply life cycle assessment | **4.90** |
| 5 | Approved | **7.10** |
| **Social Aspects** | | |
| 1 | Improve local infrastructure capacity (roads) and ensure proper services and infrastructure for all | **2.80** |
| 2 | Provide access to safe and sustainable transportation systems for all, including enhancement of road and drivers safety, and encourage carpooling and bicycle | **2.83** |
| 3 | Approved | **8.65** |

**References**

[1] M. O. Rageh, A. R. Gabr, S. M. El-Badawy, and E. E. Elbeltagi, “Highway Sustainability Factors: Holistic and Social Network Analyses,” KSCE Journal of Civil Engineering, vol. 27, no. 11, pp. 1–18, 2023, doi: 10.1007/s12205-023-0343-2.

[2] EIA Rating Guide, 2001. Environmental Impact Assessment. Environmental Management Sector, Egyptian Environmental Affairs Agency (EEAA), Ministry of State for Environmental Affairs, Egypt.

[3] BE^2^ST-in-Highways Rating System, 2010. Building Environmentally and Economically Transportation-Infrastructure-Highways. Recycled Materials Resource Center, University of Wisconsin-Madison, USA. https://rmrc.wisc.edu/be2st-in-highways.

[4] I-Last Rating System, 2010. Illinois Livability and Sustainable Transportation. Illinois Department of Transportation (IDOT), Illinois, USA.

[5] GreenLITES Rating System (D) V2.1, 2011. GreenLITES Project Environmental Sustainability Rating System. New York State Department of Transportation (NYSDOT), USA. <https://www.dot.ny.gov/programs/greenlites>.

[6] AGIC Rating Scheme V2.1, 2011. Australian Green Infrastructure Council Rating Scheme. Australia Green Infrastructure Council. Australia.

[7] GreenLITES Measures (O&M), 2012. Leadership In Transportation and Environmental Sustainability. New York State Department of Transportation (NYSDOT), USA. <https://www.dot.ny.gov/programs/greenlites>.

[8] GREENPAVE Rating System V2.1, 2017. GREENPAVE. Materials Engineering and Research Office (MERO), Ontario, Canada. <https://www.tac-atc.ca/en/conference/papers/greenpave-ontarios-pavements-sustainability-rating-system>.

[9] Envision V3.0, 2018. Envision. the Zofnass Program for Sustainable Infrastructure at the Harvard University Graduate School of Design and the Institute for Sustainable Infrastructure (ISI). <https://sustainable-infrastructure-tools.org/tools/envision-rating-system/>

[10] Invest Rating System V1.3, 2018. Infrastructure Voluntary Evaluation Sustainability Tool. The Federal Highway Administration (FHWA), USA. <https://www.sustainablehighways.org/>.

[11] LEED Rating System V4.0, 2018. Leadership in Energy and Environmental Design. U.S. Green Building Council (USGBC), USA. <https://www.usgbc.org/leed>.

[12] STARS Rating System V2.2, 2019. Sustainability Tracking, Assessment & Rating System. [The](https://www.usgbc.org/) Association for the Advancement of Sustainability in Higher Education (AASHE), USA. [https://stars.aashe.org/](file:///D:\Mohamed\Ph.D\Publication\2.0\0%20edition\%20https:\stars.aashe.org\)

[13] Greenroads Rating System V2, 2020. Greenroads. Washington University. https://www.greenroads.org/.

[14] LEED Rating System V4.1, 2021. Leadership in Energy and Environmental Design. U.S. Green Building Council (USGBC), USA. <https://www.usgbc.org/leed>.

1. Corresponding Author:

   Email: [eng.m_osami@mans.edu.eg](mailto:eng.m_osami@mans.edu.eg) [↑](#footnote-ref-1)
